# Supplementary material for: Reproducible safety and efficacy of durvalumab with or without tremelimumab for hepatocellular carcinoma in clinical practice: Results of the DT-real study
Source: JHEP Rep. 2025 Nov 20;8(3):101685. doi: 10.1016/j.jhepr.2025.101685 (PMC12886069; doi:10.1016/j.jhepr.2025.101685)
Supplement: Multimedia component 1 [file mmc1.pdf]

Reproducible safety and efficacy of durvalumab with or without tremelimumab for hepatocellular carcinoma in clinical practice: Results of the DT-real study

Ciro Celsa, Tiziana Pressiani, Naoshi Nishida, Shadi Mohamad Chamseddine, Ashwini Arvind , Michael Li, Marta Fortuny-, Najib Ben Khaled, Massimo Iavarone, Hidenori Toyoda, Ilario Giovanni Rapposelli, Andrea Casadei-Gardini, Caterina Vivaldi, Susanna Ulahannan, Haripriya Andanamala, Bernhard Scheiner, Matthias Pinter, Elena Orlandi, Claudia A. M. Fulgenzi, Giulia F. Manfredi, Pasquale Lombardi, Antonio D'Alessio, Bernardo Stefanini, Rosanna Villani, Francesca Romana Ponziani, Leonardo Stella, Ornella Carminati, Angela Dalia Ricci, Melina Gonzalez, Alba Sparacino, Gabriele Di Maria, Marco Vaccaro, Giuseppe Cabibbo, Calogero Cammà, Maria Reig-, Robin K. Kelley, Amit G. Singal, Ahmed O. Kaseb, Masatoshi Kudo, Lorenza Rimassa, David James Pinato

Table of contents

Fig. S1 ... 3
Fig. S2 ... 4
Fig. S3 ... 4
Fig. S4 ... 5
Fig. S5 ... 6
Fig. S6 ... 7
Fig. S7 ... 8
Fig. S8 ... 9

Fig. S9…… 10
Fig. S10…… 11
Fig. S11…… 12
Fig. S12…… 13
Table S1…… 14
Table S2…… 15
Table S3…… 16
Table S4…… 17
Table S5…… 18
Table S6…… 18
Table S7…… 18
Table S8…… 19
Table S9…… 20
Table S10…… 21
Table S11…… 22
Table S12…… 22
Table S13…… 22
Table S14…… 23
Table S15…… 24
Table S16…… 25
Table S17…… 26
Table S18…… 26
Table S19…… 26
Table S20…… 27
Table S21…… 28

Fig. S1. Study flow-chart.

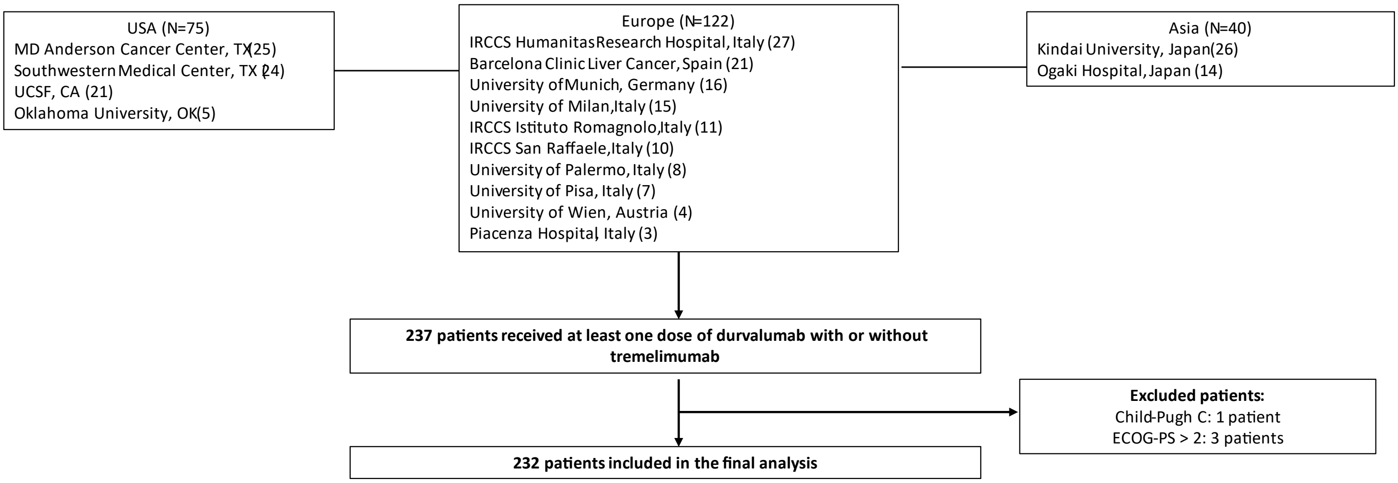

Fig. S2. Directed acyclic graph for overall survival in the whole cohort.

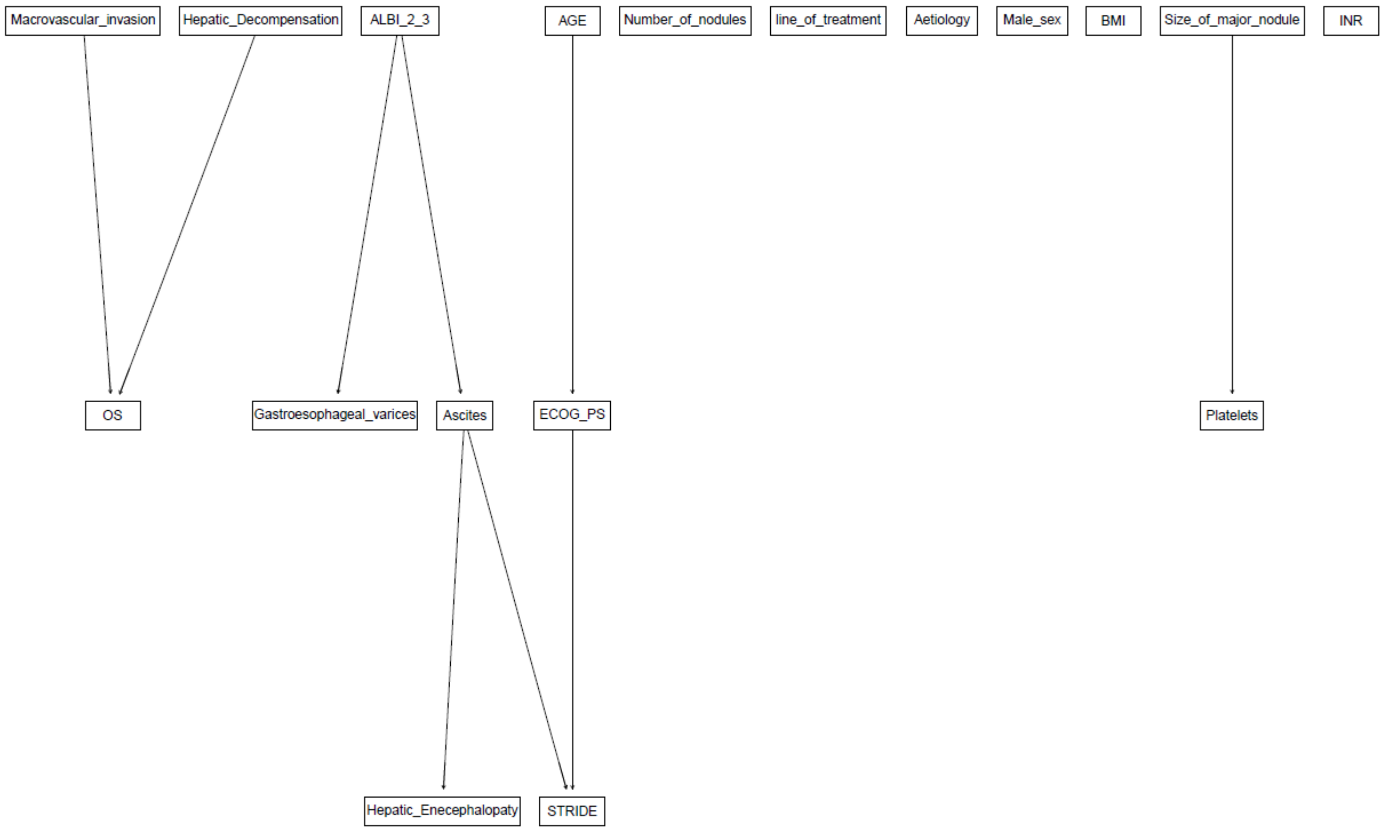

Fig. S3. Directed acyclic graph for overall survival in HIMALAYA-IN patients.

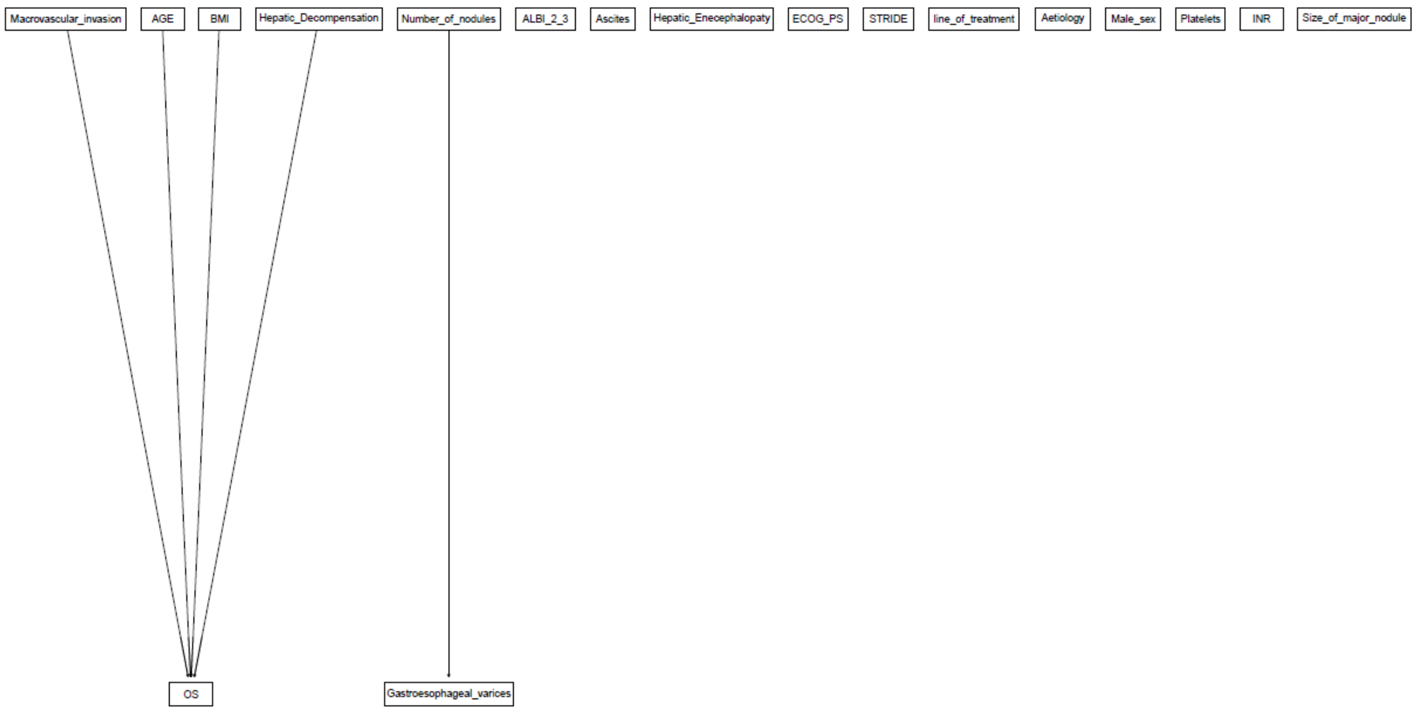

Fig. S4. Cumulative incidence function of hepatic decompensation in the whole cohort.

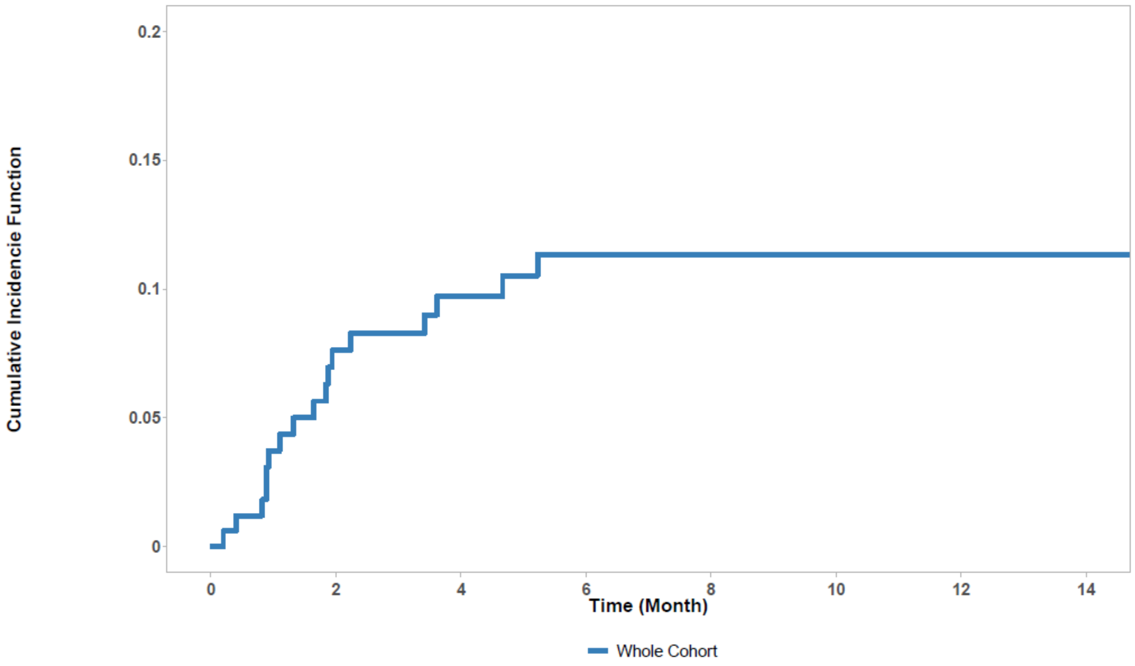

**N patients at risk**	185	133	92	73	58	49	40	39
**Whole Cohort**	0	12	15	17	17	17	17	17

Fig. S5. Simon-Makuch plot of overall survival by best radiological response (ORR vs SD vs PD) in the whole cohort.

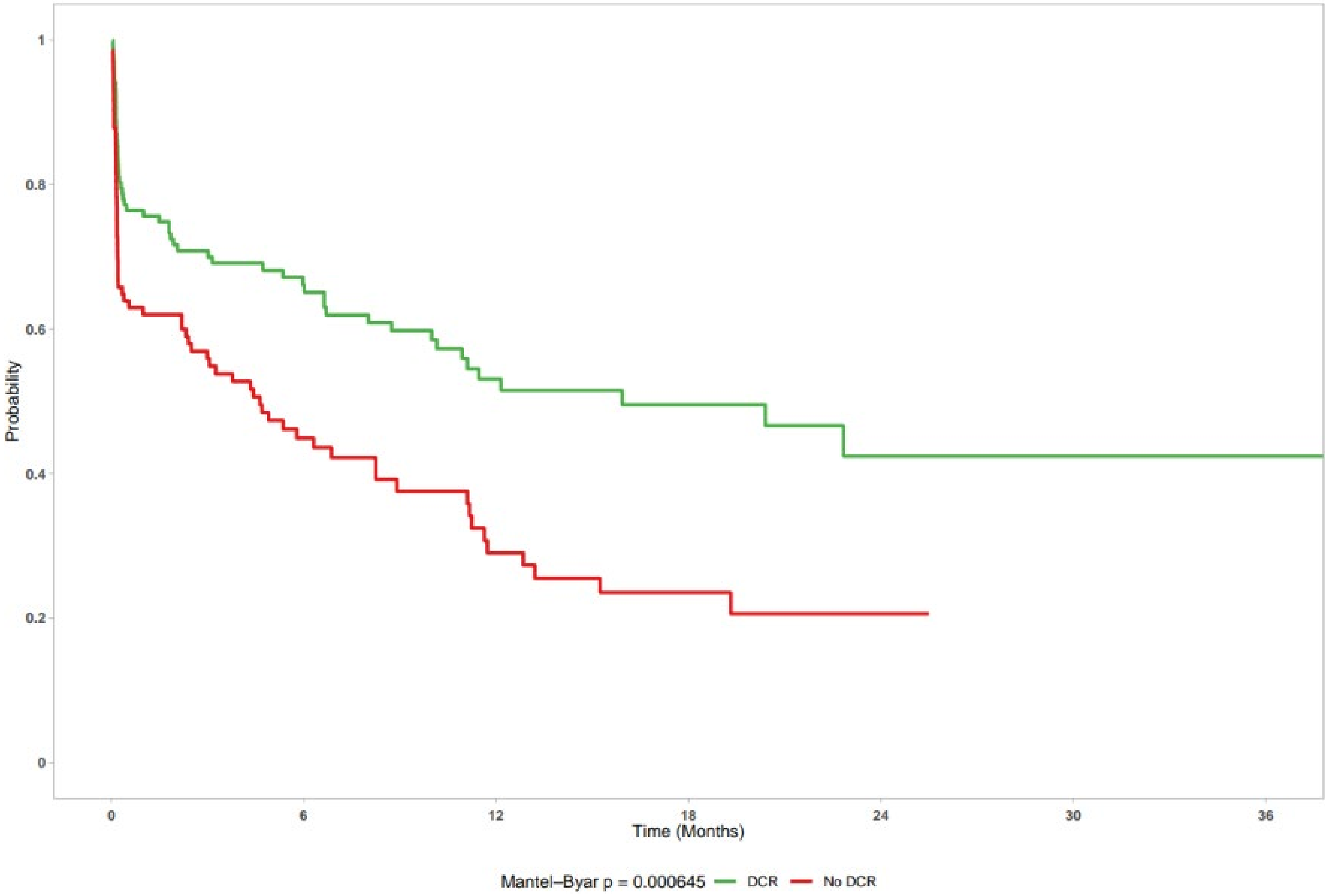

Fig. S6. Simon-Makuch plot of overall survival by best radiological response (ORR vs SD vs PD) in HIMALAYA-IN patients.

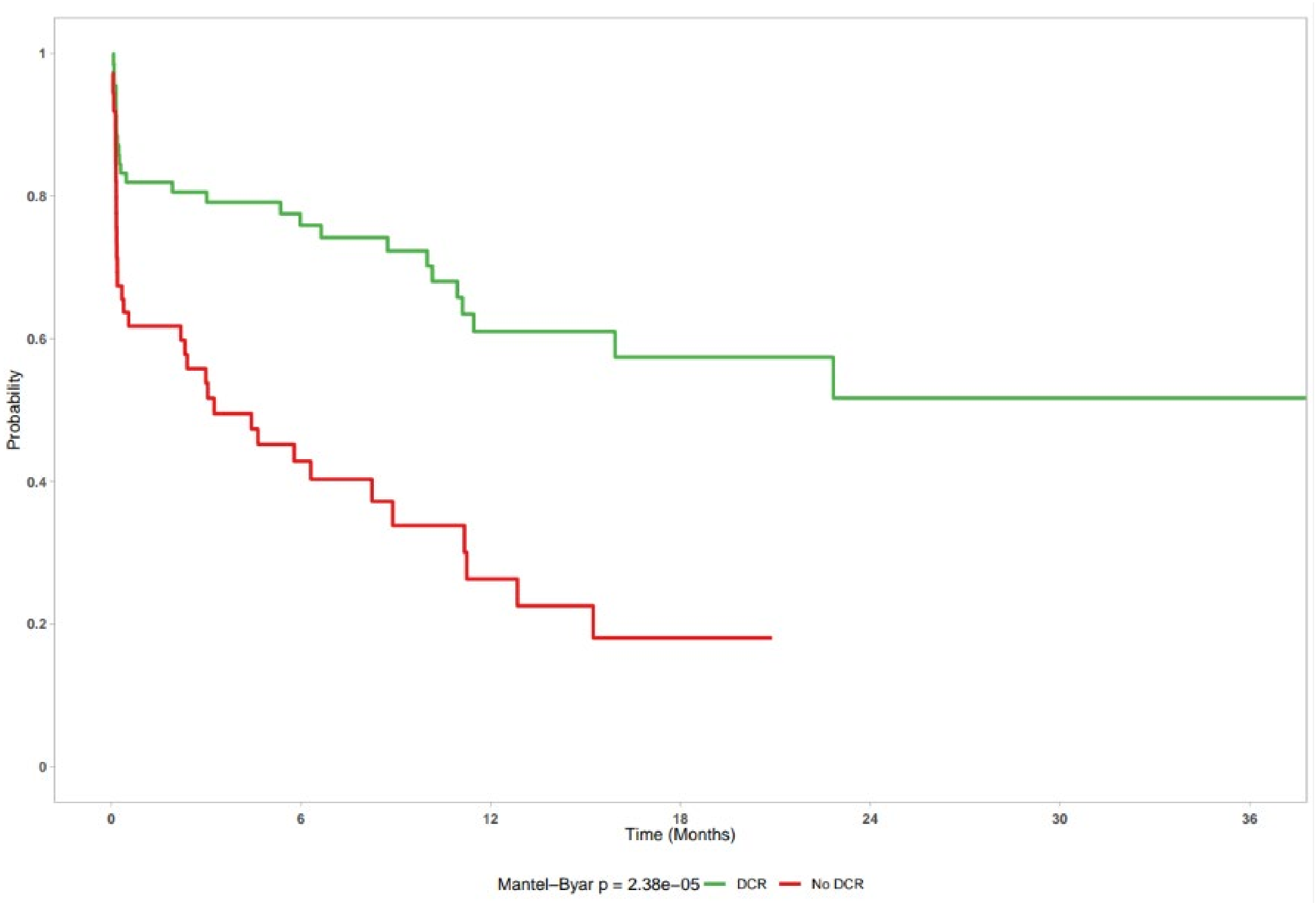

Fig. S7. Simon-Makuch plot of overall survival by best radiological response (ORR vs SD vs PD) in HIMALAYA-IN patients treated with STRIDE.

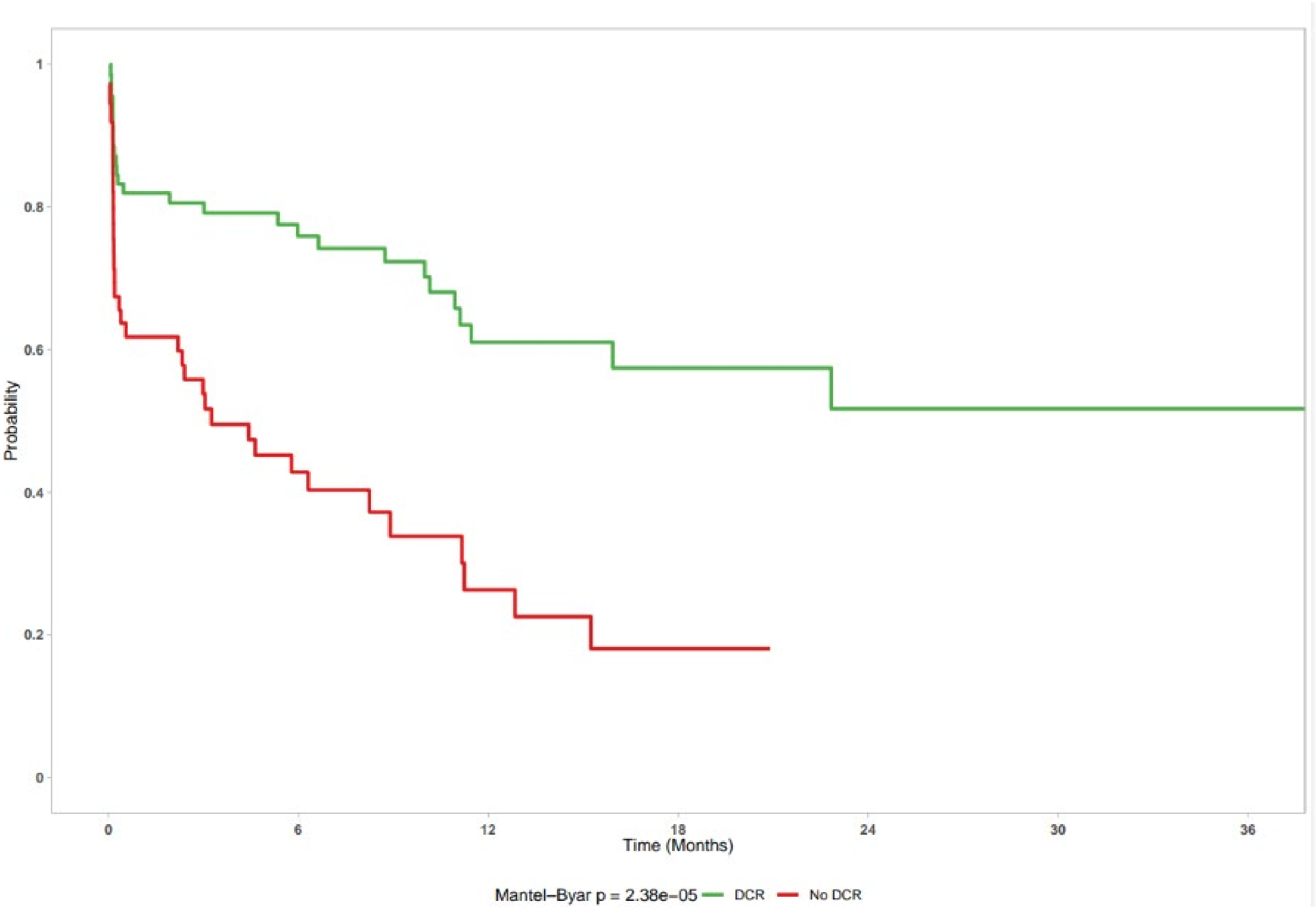

Fig. S8. Simon-Makuch plot of overall survival by best radiological response (ORR vs SD vs PD) in HIMALAYA-OUT patients.

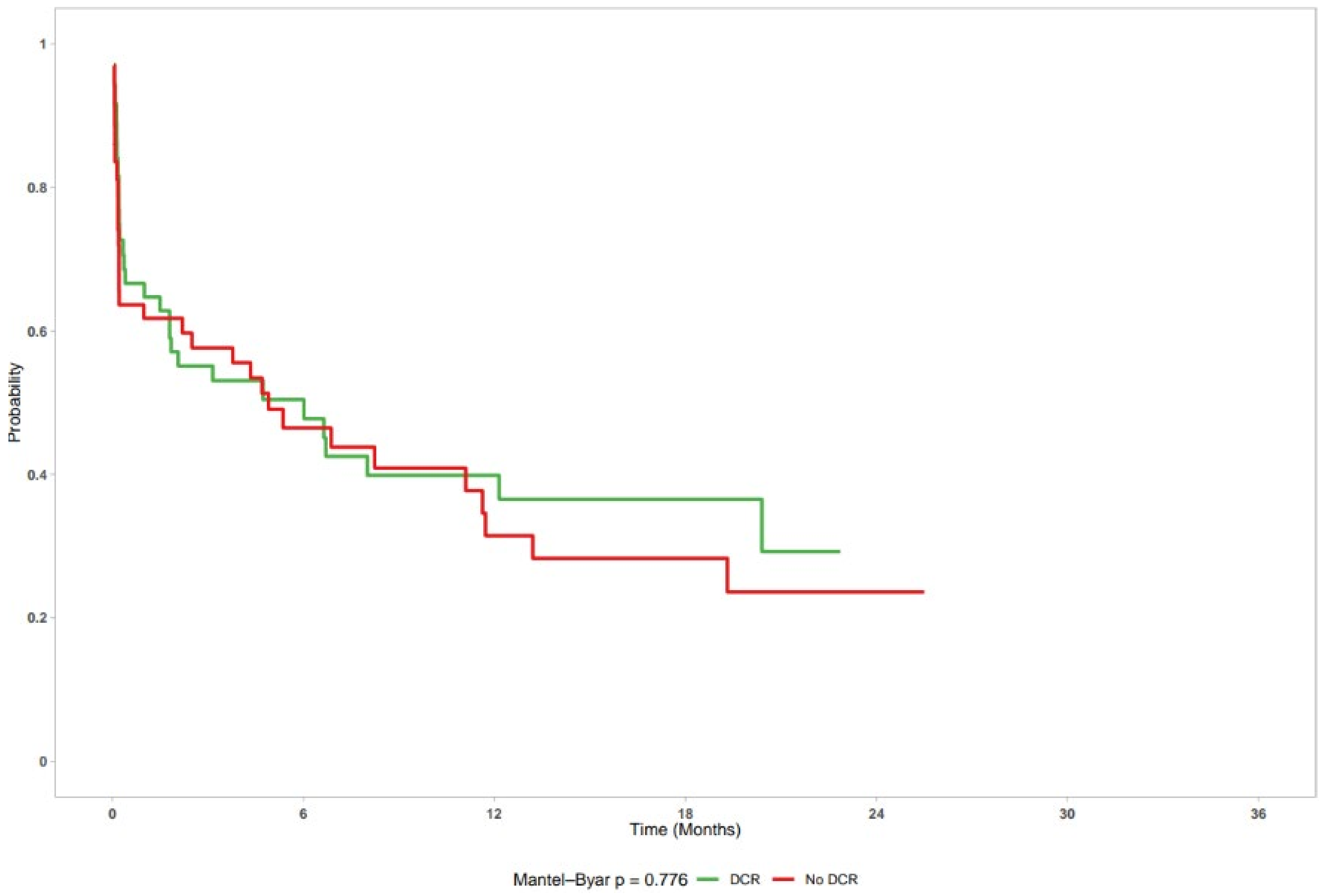

Fig. S9. Overall survival by best radiological response (ORR vs SD vs PD) in the whole cohort.

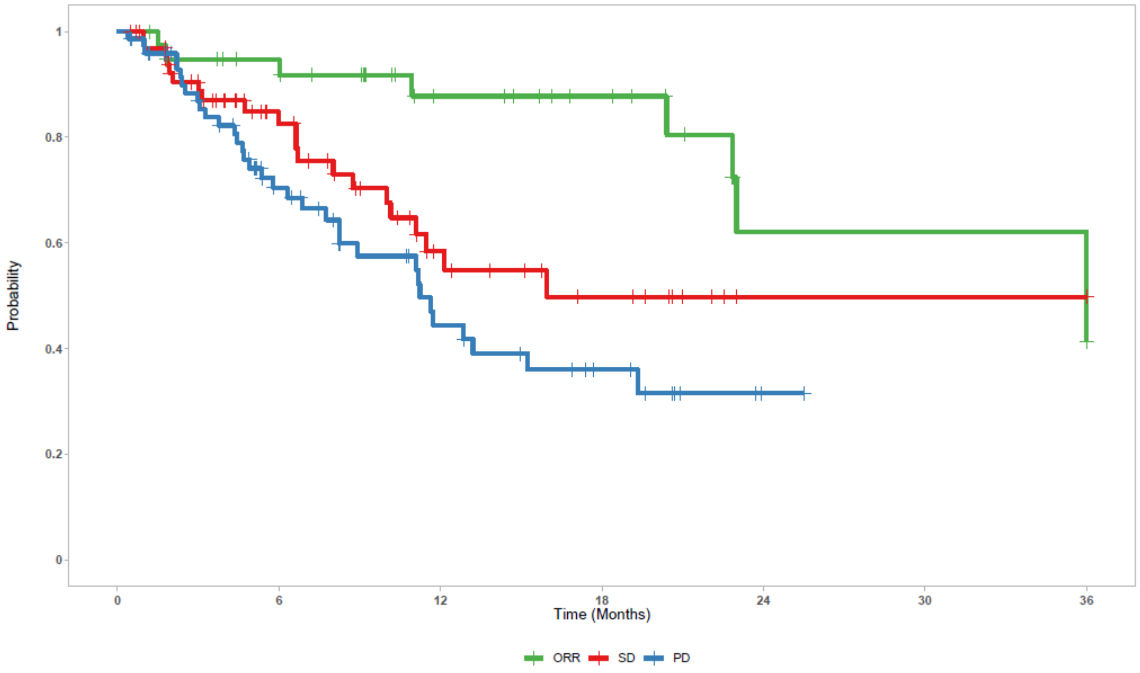

ORR	40	33	21	16	7	7	6
SD	71	37	17	10	2	2	1
PD	76	38	18	10	2	0	0

Fig. S10. Overall survival by best radiological response (ORR vs SD vs PD) in HIMALAYA IN patients.

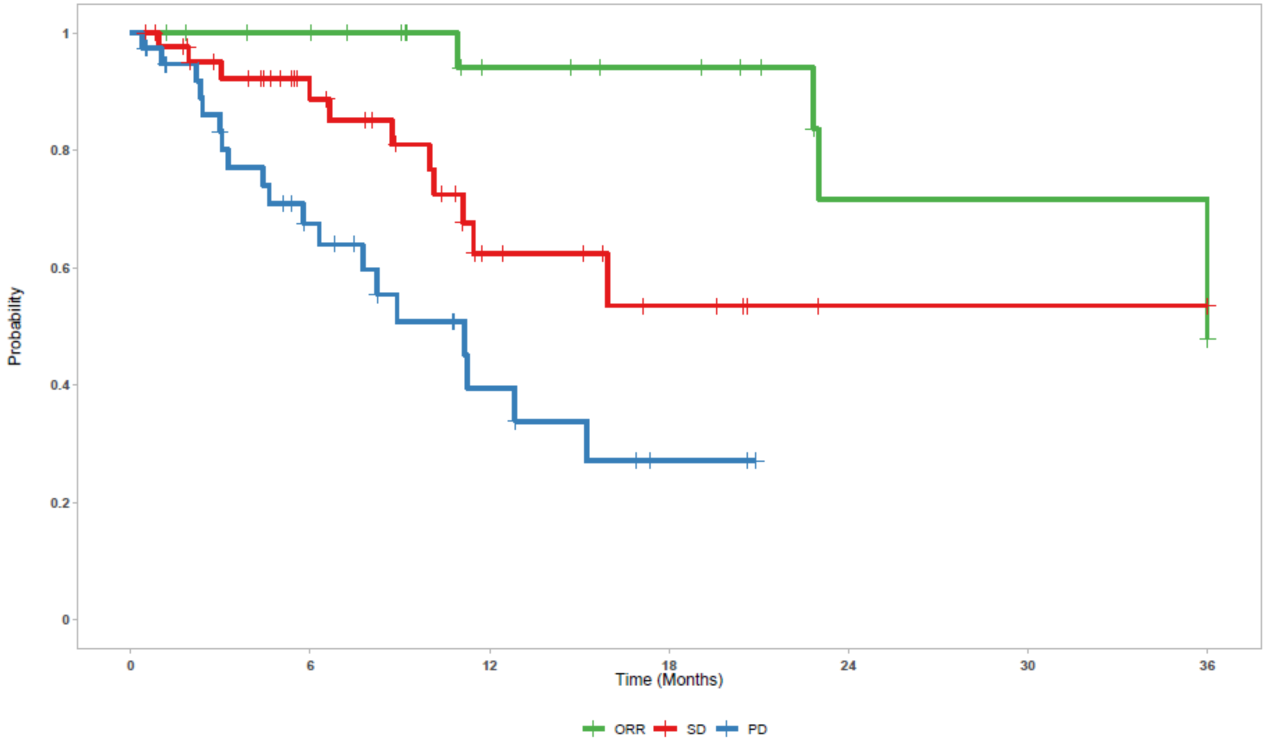

	0	6	12	18	24	30	36
ORR	27	24	15	13	7	7	6
SD	47	26	11	6	2	2	1
PD	40	20	8	3	0	0	0

<u>Fig. S11.</u> <u>Overall survival by best radiological response (ORR vs SD vs PD) in HIMALAYA IN patients treated with STRIDE.</u>

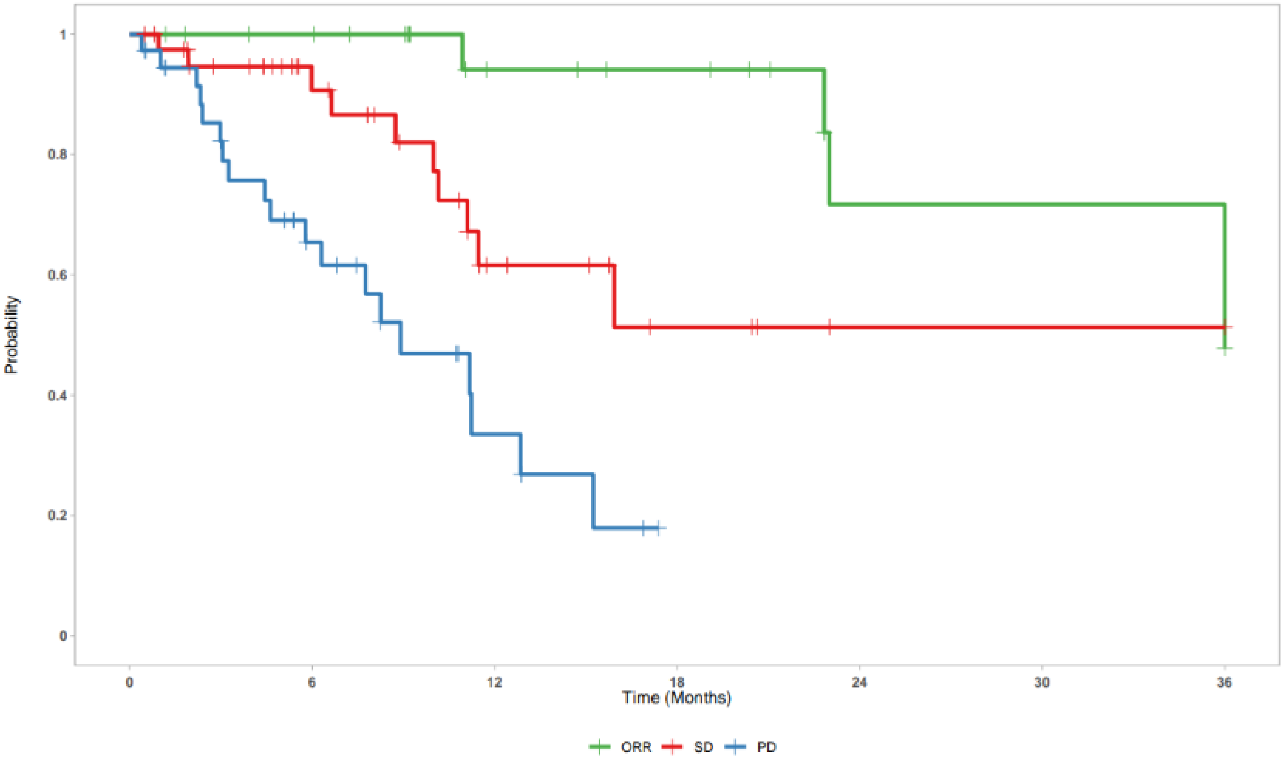

ORR	27	24	15	13	7	7	6
SD	44	24	10	5	2	2	1
PD	38	18	6	0	0	0	0

Fig. S12. Overall survival by best radiological response (ORR vs SD vs PD) in HIMALAYA OUT patients.

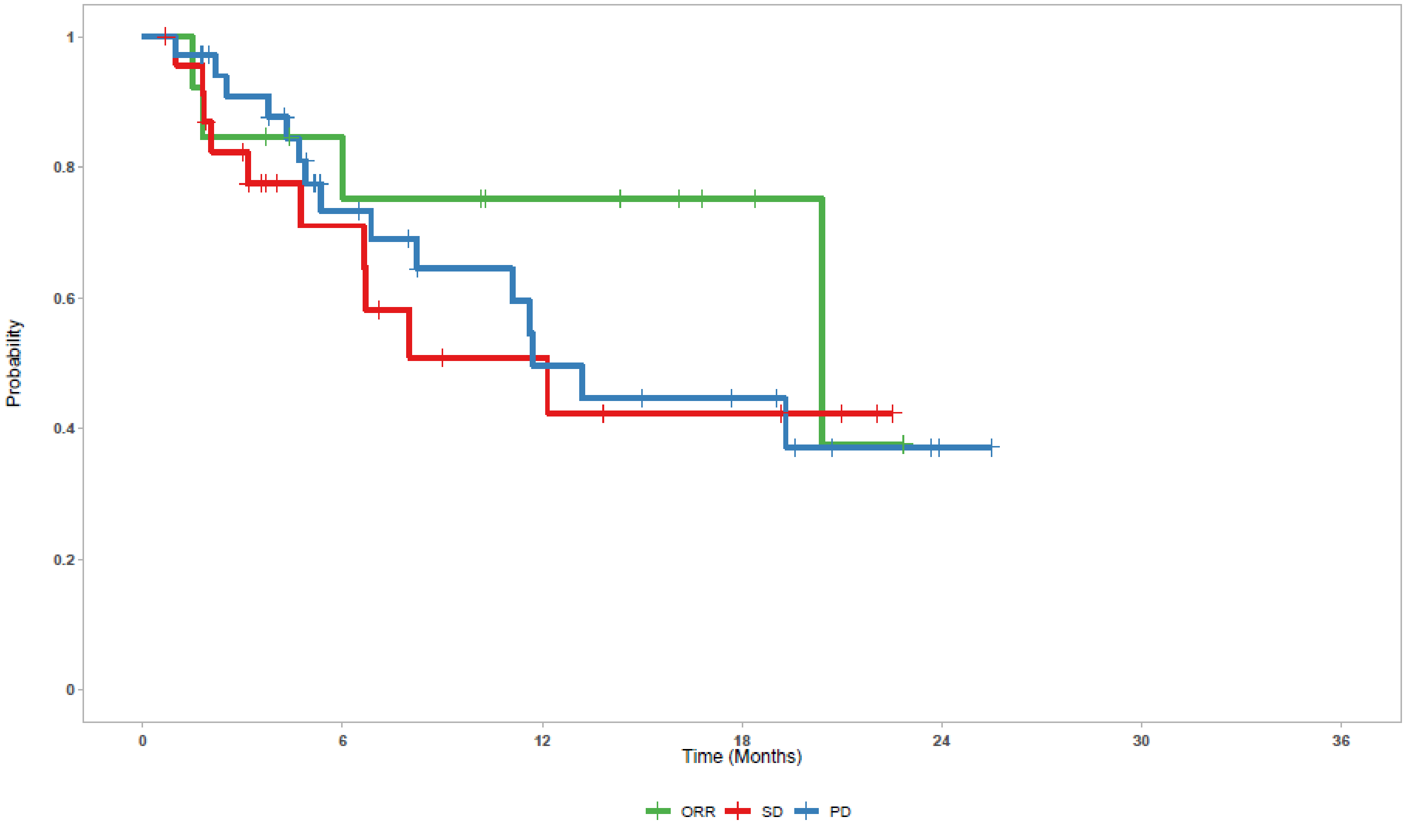

ORR	13	10	7	4	0	0	0
SD	24	12	7	5	0	0	0
PD	36	19	11	8	2	0	0

Table S1. Proportions of missing variables.

	**N (%)**
Age (years)	0 (0.0)
Sex	0 (0.0)
BMI (kg/m^2)	5 (2.1)
Type 2 diabetes	21 (9.0)
Cirrhosis	2 (0.9)
Aetiology	4 (1.7)
Previous portal hypertensive bleeding	20 (8.6)
Ascites	1 (0.4)
Hepatic encephalopathy	14 (6.0)
Platelet count (*10^9/L)	20 (8.6)
Albumin (g/dL)	21 (9.0)
Bilirubin (mg/dL)	20 (8.6)
INR	22 (9.4)
Child-Pugh class	0 (0.0)
ALBI score	21 (9.0)
ECOG-PS	0 (0)
AFP	5 (2.1)
Size of major nodule (cm)	22 (9.4)
Number of nodules > 3	20 (8.6)
Extrahepatic spread	22 (9.4)
Macrovascular invasion	1 (0.4)
BCLC stage C	23 (9.9)
STRIDE	0 (0.0)
First-line treatment	0 (0.0)

Table S2. Univariate analysis for overall survival in the whole cohort.

	Hazard ratio	95% Confidence interval	p-value
Age (years)	1.01	0.99 – 1.04	0.332
Male sex	1.26	0.69 – 2.32	0.454
BMI (kg/m^2)	0.96	0.91 – 1.01	0.111
Type 2 diabetes	1.17	0.65 – 2.08	0.598
Cirrhosis	1.24	0.71 – 2.18	0.447
Viral aetiology	1.01	0.65 – 1.56	0.963
Previous portal hypertensive bleeding	0.87	0.19 – 4.05	0.865
Gastroesophageal varices	1.30	0.78 – 2.16	0.313
Ascites	1.41	0.81 – 2.47	0.226
Hepatic encephalopathy	1.17	0.38 – 3.53	0.786
Platelet count (*10^9/L)	1.00	1.00 – 1.00	0.622
Albumin (g/dL)	0.60	0.41 – 0.88	0.001
Bilirubin (mg/dL)	1.20	0.94 – 1.53	0.148
INR	0.84	0.36 – 1.93	0.679
Child-Pugh class B	1.75	1.10 – 2.81	0.019
Child-Pugh score	1.26	1.08 – 1.46	0.003
ALBI grade 2/3	1.33	0.83 – 2.16	0.238
ALBI score	1.65	1.13 – 2.43	0.010
ECOG-PS (2 vs 0-1)	1.74	1.01-2.98	0.044
AFP $\geq$ 400 ng/mL	1.27	0.81 – 1.99	0.290
Size of major nodule (cm)	1.03	1.00 – 1.06	0.071
Number of nodules > 3	1.11	0.68 – 1.81	0.670
Extrahepatic spread	1.17	0.73 – 1.87	0.507
Macrovascular invasion	2.56	1.65 – 3.97	0.000
Vp4 portal invasion	1.17	0.59 – 2.34	0.651
BCLC stage C	1.97	1.17 – 3.29	0.010
STRIDE	1.15	0.61 – 2.16	0.658
First-line treatment	0.99	0.60 – 1.63	0.958
HIMALAYA-IN	0.61	0.39 – 0.96	0.032
HCC progression*	1.07	0.77 – 1.47	0.696
Hepatic decompensation	3.58	2.23-5.76	<0.001

*Considered as time-dependent covariates

Table S3. Univariate analysis for overall survival in HIMALAYA-IN patients.

	**Hazard ratio**	**95% Confidence interval**	**p-value**
Age (years)	1.07	1.03 – 1.11	0.001
Male sex	0.84	0.37 – 1.90	0.684
BMI (kg/m^2)	0.89	0.84 – 0.95	0.000
Type 2 diabetes	1.26	0.57 – 2.79	0.560
Cirrhosis	1.13	0.57 – 2.22	0.722
Viral aetiology	0.86	0.45 – 1.62	0.638
Previous portal hypertensive bleeding	2.17	0.32 – 14.74	0.426
Gastroesophageal varices	1.11	0.46 – 2.65	0.820
Ascites	0.46	0.09 – 2.27	0.338
Platelet count (*10^9/L)	1.00	1.00 – 1.00	0.142
Albumin (g/dL)	0.80	0.41 – 1.58	0.524
Bilirubin (mg/dL)	0.89	0.51 – 1.54	0.678
INR	0.65	0.13 – 3.25	0.602
Child-Pugh score 6	1.07	0.53 – 2.18	0.84
ALBI grade 2/3	1.00	0.54 – 1.88	0.989
ALBI score	1.26	0.69 – 2.32	0.452
ECOG-PS 1	0.79	0.40 – 1.55	0.502
AFP $\geq$ 400 ng/mL	0.79	0.40 – 1.54	0.485
Size of major nodule (cm)	1.01	0.97 – 1.05	0.482
Number of nodules > 3	1.21	0.60 – 2.45	0.600
Extrahepatic spread	0.79	0.39 – 1.61	0.512
Macrovascular invasion	3.02	1.60 – 5.73	0.001
BCLC stage C	1.48	0.76 – 2.87	0.25
STRIDE	5.25	0.67 – 41.14	0.114
HCC progression*	1.00	0.67 – 1.48	0.993
Hepatic decompensation*	3.29	1.46 – 7.39	<0.001

*Considered as time-dependent covariates

Table S4. Univariate and multivariate analysis for overall survival after imputation of missing data.

	**Univariate analysis**			**Multivariate analysis**		
	**Hazard ratio**	**95% Confidence interval**	**p-value**	**Hazard ratio**	**95% Confidence interval**	**p-value**
Age (years)	1.01	0.99 – 1.04	0.332			
Male sex	1.26	0.69 – 2.32	0.454			
BMI (kg/m^2)	1.05	0.95-1.15	0.376			
Type 2 diabetes	1.01	0.63-1.61	0.975			
Cirrhosis	1.39	0.81-2.39	0.233			
Viral aetiology	1.03	0.67-1.59	0.894			
Previous portal hypertensive bleeding	0.62	0.15-2.51	0.499			
Ascites	1.50	0.89-2.52	0.125			
Hepatic encephalopathy	0.94	0.31-2.90	0.919			
Platelet count (*10^9/L)	1.00	0.99-1.01	0.731			
INR	1.14	0.49-2.64	0.759			
Child-Pugh class B	1.75	1.10 – 2.81	0.019			
Child-Pugh score	1.26	1.08 – 1.46	0.003			
ALBI grade 2/3	1.41	0.87-2.27	0.159			
ALBI score	1.67	1.11-2.50	0.013	1.42	0.93-2.14	0.097
ECOG-PS (2 vs 0-1)	1.74	1.01-2.98	0.044	1.23	0.71-2.10	0.457
AFP $\geq$ 400 ng/mL	1.26	0.81-1.96	0.298			
Size of major nodule (cm)	1.04	0.96-1.13	0.316			
Number of nodules > 3	1.21	0.79-1.86	0.376			
Extrahepatic spread	1.22	0.78-1.93	0.383			
Macrovascular invasion	2.41	1.55-3.73	<0.001	2.13	1.35-3.35	0.001
Vp4 portal invasion	1.17	0.59 – 2.34	0.651			
BCLC stage C	1.92	1.18-3.13	0.009			
STRIDE	1.15	0.61 – 2.16	0.658			
First-line treatment	0.99	0.60 – 1.63	0.958			
HIMALAYA-IN	0.61	0.39 – 0.96	0.032			
HCC progression*	1.07	0.77 – 1.47	0.696			
Hepatic decompensation	3.58	2.23-5.76	<0.001	3.54	1.89-6.63	<0.001

Table S5. Progression-free survival of HIMALAYA-OUT patients stratified according to exclusion criteria.

	Child-Pugh B	Child-Pugh A	HR (95%CI)	ECOG-PS > 1	ECOG-PS 0-1	HR (95%CI)	Vp4	No Vp4	HR (95%CI)	>1° Line	1° Line	HR (95%CI)
Median progression-free survival (months)(95% CI)	3.8 (2.5-5.2)	5.0 (2.5-9.9)	1.18, (0.70-1.98, p=0.53)	4.4 (3.5-20.4)	3.6 (2.4-5.0)	0.69, (0.41-1.15, p=0.16)	5.2 (2.1-20.4)	3.7 (2.6-5.0)	0.77, (0.42-1.43, p=0.41)	4.3 (2.4-6.7)	3.7 (2.5-9.9)	0.92, (0.55-1.54, p=0.77)

Table S6. Progression-free survival of STRIDE and Durvalumab monotherapy in HIMALAYA-IN and HIMALAYA-OUT patients.

	HIMALAYA-IN			HIMALAYA-OUT		
	STRIDE	Durvalumab monotherapy	HR (95%CI, p-value)	STRIDE	Durvalumab monotherapy	HR (95%CI, p-value)
Median progression-free survival (months)(95%CI)	6.6 (6.0-11.1)	5.3 (1.9-11.3)	0.75 (0.27-2.08, p=0.58)	3.9 (2.9-6.7)	3.7 (2.5-20.4)	0.92 (0.54-1.59, p=0.77)

Table S7. Progression-free survival according to aetiology of liver disease in the whole cohort, in HIMALAYA-IN and HIMALAYA-OUT patients.

	Whole cohort			HIMALAYA-IN			HIMALAYA-OUT		
	Viral	Non-viral	HR (95%CI, p-value)	Viral	Non-viral	HR (95%CI, p-value)	Viral (n=32)	Non-viral (n=41)	HR (95%CI, p-value)
Median progression-free survival (months)(95%CI)	6.3 (4.3-9.1)	5.3 (3.7-6.6)	0.93 (0.66-1.32, p=0.70)	9.1 (6.0-15.2)	6.2 (3.3-8.4)	0.82. (0.51-1.32, p=0.41)	3.7 (2.5-5.2)	4.4 (2.4-9.9)	1.15, (0.69-1.92, p=0.59)

Table S8. Univariate analysis for progression-free survival in the whole cohort.

	**Hazard ratio**	**95% Confidence interval**	**p-value**
Age (years)	1.02	1.00 – 1.04	0.109
Male sex	1.00	0.64 – 1.54	0.985
BMI (kg/m^2)	0.97	0.93 – 1.01	0.115
Type 2 diabetes	1.01	0.64 – 1.60	0.958
Cirrhosis	1.17	0.78 – 1.76	0.449
Viral aetiology	0.93	0.66 – 1.32	0.702
Previous portal hypertensive bleeding	0.81	0.30 – 2.22	0.690
Gastroesophageal varices	1.36	0.93 – 2.00	0.114
Ascites	1.26	0.81 – 1.97	0.305
Hepatic encephalopathy	0.68	0.25 – 1.85	0.454
Platelet count (*10^9/L)	1.00	1.00 – 1.00	0.531
Albumin (g/dL)	0.73	0.53 - 1.00	0.051
Bilirubin (mg/dL)	1.24	1.01 - 1.52	0.035
INR	0.94	0.46 - 1.95	0.877
Child-Pugh class B	1.44	0.98 - 2.12	0.066
Child-Pugh score	1.14	1.00 - 1.31	0.047
ALBI grade 2/3	1.05	0.73 - 1.50	0.797
ALBI score	1.34	0.98 - 1.82	0.062
ECOG-PS (2 vs 0-1)	0.97	0.61 - 1.52	0.885
AFP ≥ 400 ng/mL	0.98	0.69 - 1.40	0.908
Size of major nodule (cm)	1.01	0.98 - 1.04	0.488
Number of nodules > 3	1.38	0.95 - 2.01	0.091
Extrahepatic spread	1.25	0.87 - 1.81	0.226
Macrovascular invasion	1.42	0.99 - 2.04	0.060
Vp4 portal invasion	0.91	0.49 - 1.70	0.776
BCLC stage C	1.41	0.96 - 2.09	0.082
STRIDE	0.88	0.57 - 1.39	0.603
First-line treatment	0.78	0.51 - 1.18	0.243
HIMALAYA-IN	0.74	0.52 - 1.05	0.091

Table S9. Univariate analysis for progression-free survival in HIMALAYA-IN patients.

	**Hazard ratio**	**95% Confidence interval**	**p-value**
Age (years)	1.07	1.03 - 1.10	<0.001
Male sex	0.86	0.48 - 1.53	0.607
BMI (kg/m^2)	0.95	0.89 - 1.00	0.064
Type 2 diabetes	1.15	0.63 - 2.09	0.646
Cirrhosis	1.06	0.64 - 1.74	0.821
Viral aetiology	0.82	0.51 - 1.32	0.413
Previous portal hypertensive bleeding	1.33	0.32 - 5.52	0.689
Gastroesophageal varices	1.41	0.79 - 2.51	0.246
Ascites	0.89	0.32 - 2.45	0.822
Platelet count (*10^9/L)	1.00	1.00 - 1.00	0.155
Albumin (g/dL)	1.18	0.72 - 1.95	0.512
Bilirubin (mg/dL)	0.95	0.58 - 1.57	0.856
INR	1.19	0.41 - 3.46	0.748
Child-Pugh score	0.79	0.46 - 1.38	0.415
ALBI grade 2/3	0.79	0.50 - 1.26	0.325
ALBI score	0.83	0.51 - 1.36	0.467
ECOG-PS 1	1.14	0.70 - 1.85	0.591
AFP $\geq$ 400 ng/mL	0.66	0.40 - 1.09	0.107
Size of major nodule (cm)	1.00	0.96 - 1.05	0.853
Number of nodules > 3	1.06	0.64 - 1.78	0.813
Extrahepatic spread	1.17	0.72 - 1.92	0.526
Macrovascular invasion	1.52	0.89 - 2.58	0.123
BCLC stage C	1.35	0.82 - 2.22	0.240
STRIDE	0.77	0.31 - 1.93	0.585

Table S10. Univariate competing risks analysis for hepatic decompensation in the whole cohort.

	Univariate analysis			Multivariable analysis		
	**Subdistribution Hazard ratio**	**95% Confidence interval**	**p-value**	**Subdistribution Hazard ratio**	**95% Confidence interval**	**p-value**
Age (years)	0.98	0.95 - 1.02	0.319			
Male sex	1.22	0.32 - 4.62	0.767			
BMI (kg/m^2)	1.01	0.93 - 1.10	0.775			
Type 2 diabetes	0.68	0.16 - 2.96	0.609			
Cirrhosis	1.66	0.50 - 5.52	0.406			
Viral aetiology	1.58	0.61 - 4.09	0.347			
Previous portal hypertensive bleeding	2.09	0.61 - 4.09	0.392			
Gastroesophageal varices	0.53	0.15 - 1.83	0.318			
Platelet count (*10^9/L)	0.99	0.98 - 1.01	0.655			
Albumin (g/dL)	0.62	0.30 - 1.25	0.180			
Bilirubin (mg/dL)	1.34	0.90 - 1.99	0.145			
INR	0.73	0.15 - 3.34	0.684			
Child-Pugh score	1.37	0.93 - 2.02	0.110			
ALBI grade 2/3	1.39	0.49 - 3.89	0.559			
ALBI score	1.83	0.95 - 3.54	0.072	1.67	0.85-3.28	0.135
ECOG-PS 2 (vs 0-1)	1.84	0.58 - 5.80	0.296			
AFP $\geq$ 400 ng/mL	0.99	0.98 - 1.01	0.611			
Size of major nodule (cm)	1.04	0.99 - 1.08	0.056	1.03	0.99-1.08	0.111
Number of nodules > 3	2.66	0.89 - 7.97	0.080	2.50	0.83-7.51	0.102
Extrahepatic spread	1.52	0.53 - 4.32	0.435			
Macrovascular invasion	1.77	0.69 - 4.54	0.237			
BCLC stage C	2.20	0.64 - 7.61	0.213			
STRIDE	0.97	0.37 - 2.53	0.949			
First-line treatment	1.58	0.36-6.94	0.541			

Table S11. Objective response rate of HIMALAYA-OUT patients stratified according to exclusion criteria.

	Child-Pugh B (n=38)	Child-Pugh A (n=35)	p-value	ECOG-PS > 1 (n=22)	ECOG-PS 0-1 (n=51)	p-value	Vp4 (n=14)	No Vp4 (n=59)	p-value	>1° Line (n=31)	1° Line (n=42)	p-value
CR	1 (2.6)	1 (2.9)	0.994	0 (0.0)	2 (3.9)	0.468	0 (0.0)	2 (3.4)	0.272	1 (3.2)	1 (2.4)	0.373
PR	6 (15.8)	5 (14.3)		5 (22.7)	6 (11.8)		5 (35.7)	6 (10.2)		3 (9.7)	8 (19.0)	
SD	12 (31.6)	12 (34.3)		8 (36.4)	16 (31.4)		3 (31.4)	21 (35.6)		10 (32.3)	14 (33.3)	
PD	19 (50.0)	17 (48.6)		9 (40.9)	27 (52.9)		6 (42.9)	30 (50.8)		17 (54.8)	19 (45.2)	
ORR	7 (18.4)	6 (17.1)	0.887	5 (22.7)	8 (15.7)	0.474	5 (35.7)	8 (13.5)	0.053	4 (12.9)	9 (21.4)	0.350
DCR	19 (50.0)	18 (51.4)	0.904	13 (59.1)	24 (47.1)	0.349	8 (57.1)	29 (49.1)	0.593	14 (45.2)	25 (59.5)	0.421

Table S12. Objective response rate of STRIDE and Durvalumab monotherapy in HIMALAYA-IN and HIMALAYA-OUT patients.

	Whole cohort (n=187)			HIMALAYA-IN (n=114)			HIMALAYA-OUT (n=73)		
	STRIDE (n=155)	Durvalumab monotherapy (n=32)	p-value	STRIDE (n=109)	Durvalumab monotherapy (n=5)	p-value	STRIDE (n=46)	Durvalumab monotherapy (n=27)	p-value
CR	9 (5.8)	0 (0.0)	0.226	7 (6.4)	0 (0.0)	0.365	2 (4.3)	0 (0.0)	0.945
PR	26 (16.8)	5 (15.6)		20 (18.3)	0 (0.0)		6 (13.0)	5 (18.5)	
SD	59 (38.1)	12 (37.5)		44 (40.4)	3 (60.0)		15 (32.6)	9 (33.3)	
PD	61 (39.4)	15 (46.9)		38 (34.9)	2 (40.0)		23 (50.0)	13 (48.1)	
ORR	35 (22.6)	5 (15.6)	0.383	27 (24.8)	0 (0.0)	0.205	8 (17.4)	5 (18.5)	0.904
DCR	94 (60.6)	17 (53.1)	0.432	71 (65.1)	3 (60.0)	0.815	23 (50.0)	14 (51.9)	0.879

Table S13. Objective response rate according to aetiology of liver disease in the whole cohort, in HIMALAYA-IN and HIMALAYA-OUT patients.

	Whole cohort (n=187)			HIMALAYA-IN (n=114)			HIMALAYA-OUT (n=73)		
	Viral (n=84)	Non-viral (n=103)	p-value	Viral (n=52)	Non-viral (n=62)	p-value	Viral (n=32)	Non-viral (n=41)	p-value
CR	3 (3.6)	6 (5.8)	0.625	2 (3.8)	5 (8.1)	0.786	1 (3.1)	1 (2.4)	0.605
PR	15 (17.9)	16 (15.5)		11 (21.2)	9 (14.5)		4 (12.5)	7 (17.1)	
SD	30 (35.7)	41 (39.8)		20 (38.5)	27 (43.5)		10 (31.2)	14 (34.1)	
PD	36 (42.9)	40 (38.8)		19 (36.5)	21 (33.9)		17 (53.1)	19 (46.3)	

ORR	18 (21.4)	22 (21.4)	0.991	13 (25.0)	14 (22.6)	0.763	5 (15.6)	8 (19.5)	0.667
DCR	48 (57.1)	63 (61.2)	0.578	33 (63.5)	41 (66.1)	0.767	15 (46.9)	22 (53.7)	0.568

Table S14. Univariate and multivariate logistic regression analysis for objective response rate in the whole cohort.

	**Univariate analysis**			**Multivariable analysis**		
	**Odds ratio**	**95% Confidence interval**	**p-value**	**Odds ratio**	**95% Confidence interval**	**p-value**
Age (years)	0.97	0.94-1.01	0.130			
Male sex	0.58	0.23-1.46	0.248			
BMI (kg/m^2)	1.02	0.94-1.09	0.649			
Type 2 diabetes	1.03	0.44-2.42	0.950			
Cirrhosis	0.78	0.34-1.80	0.568			
Viral aetiology	1.02	0.50-2.08	0.955			
Gastroesophageal varices	0.87	0.39-1.96	0.746			
Ascites	1.78	0.71.4.48	0.222			
Hepatic encephalopathy	0.51	0.06-4.35	0.545			
Platelet count (*10^9/L)	1.00	0.99-1.01	0.884			
Albumin (g/dL)	0.53	0.29-0.98	0.042			
Bilirubin (mg/dL)	0.99	0.591-67	0.975			
INR	2.20	0.68-7.14	0.189			
Child-Pugh class B	0.86	0.34-2.15	0.746			
Child-Pugh score	1.04	0.77-1.40	0.816			
ALBI grade 2/3	1.85	0.85-4.00	0.121			
ALBI score	1.73	0.95-3.14	0.073	1.59	0.86-3.00	0.145
ECOG-PS 2 (vs 0-1)	1.12	0.38-3.28	0.834			
AFP ≥ 400 ng/mL	2.36	1.14-4.89	0.021	2.17	1.01-4.64	0.047
Size of major nodule (cm)	0.99	0.94-1.06	0.937			
Number of nodules > 3	0.57	0.26-1.23	0.153			
Extrahepatic spread	1.32	0.60-2.89	0.486			
Macrovascular invasion	2.08	0.97-4.43	0.059	1.45	0.65-3.23	0.364
Vp4 portal invasion	2.11	0.66-6.70	0.206			
BCLC stage C	2.24	0.97-5.16	0.058			
STRIDE	1.58	0.56-4.43	0.385			
First-line treatment	2.13	0.70-6.50	0.185			
HIMALAYA-IN	1.41	0.67-2.97	0.370			

Table S15. Univariate and multivariable logistic regression analysis for objective response rate in HIMALAYA-IN patients.

	Univariate analysis			Multivariable analysis		
	**Odds ratio**	**95% Confidence interval**	**p-value**	**Odds ratio**	**95% Confidence interval**	**p-value**
Age (years)	0.94	0.89-1.00	0.051	0.93	0.85-0.99	0.029
Male sex	0.36	0.11-1.17	0.091	0.87	0.09-2.81	0.440
BMI (kg/m^2)	1.02	0.92-1.13	0.740			
Type 2 diabetes	1.13	0.40-3.18	0.811			
Cirrhosis	1.17	0.45-30.03	0.750			
Viral aetiology	1.19	0.49-2.88	0.700			
Gastroesophageal varices	0.68	0.21-2.25	0.533			
Ascites	1.67	0.29-9.73	0.566			
Platelet count (*10^9/L)	1.00	0.99-1.01	0.292			
Albumin (g/dL)	0.30	0.12-0.78	0.013			
Bilirubin (mg/dL)	0.72	0.26-1.99	0.528			
INR	0.19	0.01-8.98	0.396			
Child-Pugh score	1.42	0.55-3.65	0.467			
ALBI grade 2/3	2.21	0.89-5.47	0.085			
ALBI score	2.88	1.07-7.72	0.035	3.24	0.96-10.86	0.058
AFP $\geq$ 400 ng/mL	2.67	1.06-6.68	0.036	2.00	0.66-6.15	0.221
Size of major nodule (cm)	0.99	0.92-1.06	0.767			
Number of nodules > 3	0.55	0.21-1.42	0.216			
Extrahepatic spread	1.46	0.56-3.78	0.439			
Macrovascular invasion	1.65	0.61-4.43	0.323			
BCLC stage C	2.31	0.86-6.18	0.096	2.63	0.80-8.72	0.113

Table S16. Treatment-related adverse events in the whole cohort and in HIMALAYA-IN patients.

	Whole cohort		HIMALAYA-IN patients	
**Toxicities**	**Any-grade TRAEs**	**Grade 3-4 TRAEs**	**Any-grade TRAEs**	**Grade 3-4 TRAEs**
Skin	35 (15.0%)	4 (1.7%)	25 (20.3%)	4 (3.2%)
Diarrhoea/Colitis	27 (11.6%)	11 (4.7%)	20 (16.3%)	8 (6.5%)
Fatigue	23 (9.9%)	0 (0.0%)	12 (9.7%)	0 (0.0%)
Thyroid toxicity	15 (6.4%)	3 (1.3%)	14 (11.4%)	3 (2.4%)
Hepatotoxicity	10 (4.3%)	9 (3.9%)	5 (4.1%)	5 (4.1%)
Pneumonitis	6 (2.6%)	5 (2.1%)	4 (3.2%)	3 (2.4%)
Pituitary	5 (2.1%)	1 (0.4%)	2 (1.6%)	0 (0.0%)
Rheumatological Toxicities	3 (3.1%)	2 (0.8%)	3 (2.4%)	2 (1.6%)
Others	16 (6.9%)	5 (2.1%)	14 (11.4%)	4 (3.2%)

Table S17. Safety results of HIMALAYA-OUT patients stratified according to exclusion criteria.

	Child-Pugh B (n=70)	Child-Pugh A (n=40)	p-value	ECOG-PS > 1 (n=54)	ECOG-PS 0-1 (n=56)	p-value	Vp4 (n=22)	No Vp4 (n=88)	p-value	>1° Line (n=47)	1° Line (n=63)	p-value
Grade 3-4 TRAEs	6 (8.6)	7 (17.5)	0.165	5 (9.3)	8 (14.3)	0.416	6 (27.3)	7 (8.0)	0.013	9 (19.1)	4 (6.3)	0.041

Table S18. Safety results of STRIDE and durvalumab monotherapy in HIMALAYA-IN and -OUT patients.

	HIMALAYA-IN			HIMALAYA-OUT		
	STRIDE (n=117)	Durvalumab monotherapy (n=6)	p-value	STRIDE (n=75)	Durvalumab monotherapy (n=35)	p-value
Grade 3-4 TRAEs	23 (19.7)	2 (33.3)	0.419	8 (10.7)	5 (14.3)	0.059

Table S19. Safety according to aetiology of liver disease in the whole cohort, in HIMALAYA-IN and HIMALAYA-OUT patients.

	Whole cohort			HIMALAYA-IN			HIMALAYA-OUT		
	Viral (n=108)	Non-viral (n=125)	p-value	Viral (n=57)	Non-viral (n=66)	p-value	Viral (n=51)	Non-viral (n=59)	p-value
Grade 3-4 TRAEs	22 (20.4)	16 (12.8)	0.120	12 (21.1)	13 (19.7)	0.853	10 (19.6)	3 (5.1)	0.019

Table S20. Univariate logistic regression analysis for grade 3-4 treatment-related adverse events (TRAEs) in the whole cohort.

	**Univariate analysis**		
	**Odds ratio**	**95% Confidence interval**	**p-value**
Age (years)	0.98	0.94 - 1.01	0.180
Male sex	1.05	0.40 - 2.71	0.924
BMI (kg/m^2)	1.00	0.93 - 1.08	0.894
Type 2 diabetes	0.74	0.32 - 1.74	0.490
Cirrhosis	0.66	0.30 - 1.45	0.301
Viral aetiology	1.74	0.86 - 3.52	0.122
Previous portal hypertensive bleeding	0.40	0.05 - 3.18	0.384
Gastroesophageal varices	0.53	0.21 - 1.34	0.179
Ascites	0.44	0.15 - 1.32	0.142
Hepatic encephalopathy	0.48	0.06 - 3.85	0.488
Platelet count (*10^9/L)	1.00	1.00 - 1.00	0.755
Albumin (g/dL)	0.92	0.51 - 1.67	0.794
Bilirubin (mg/dL)	0.70	0.39 - 1.27	0.246
INR	0.40	0.05 - 2.84	0.357
Child-Pugh class B	0.38	0.15 - 0.96	0.042
Child-Pugh score	0.78	0.58 - 1.05	0.101
ALBI grade 2/3	1.37	0.64 - 2.94	0.412
ALBI score	0.98	0.54 - 1.76	0.937
ECOG-PS 2 (vs 0-1)	0.45	0.17 - 1.22	0.117
AFP $\geq$ 400 ng/mL	0.98	0.47 - 2.01	0.951
Size of major nodule (cm)	1.00	0.94 - 1.06	0.919
Number of nodules > 3	1.03	0.49 - 2.15	0.934
Extrahepatic spread	1.02	0.49 - 2.14	0.954
Macrovascular invasion	1.37	0.66 - 2.83	0.399
Vp4 portal invasion	2.10	0.76 - 5.76	0.151
BCLC stage C	0.98	0.47 - 2.05	0.954
STRIDE	0.93	0.38 - 2.30	0.884
First-line treatment	0.78	0.34 - 1.78	0.556
HIMALAYA-IN	1.90	0.92 - 3.94	0.082

Table S21. Univariate and multivariable logistic regression analysis for grade 3-4 treatment-related adverse events (TRAEs) in HIMALAYA-IN patients.

	Univariate analysis		
	**Odds ratio**	**95% Confidence interval**	**p-value**
Age (years)	0.94	0.89 - 0.99	0.026
Male sex	1.32	0.35 - 4.99	0.677
BMI (kg/m^2)	1.03	0.93 - 1.14	0.549
Type 2 diabetes	1.00	0.36 - 2.75	0.996
Cirrhosis	0.81	0.32 - 2.03	0.651
Viral aetiology	1.09	0.45 - 2.62	0.852
Previous portal hypertensive bleeding	1.23	0.12 - 12.40	0.859
Gastroesophageal varices	0.74	0.23 - 2.41	0.620
Ascites	0.56	0.07 - 4.83	0.602
Platelet count (*10^9/L)	1.00	1.00 - 1.00	0.523
Albumin (g/dL)	0.70	0.30 - 1.66	0.420
Bilirubin (mg/dL)	0.49	0.16 - 1.46	0.203
INR	0.65	0.06 - 6.73	0.715
Child-Pugh score	1.24	0.48 - 3.20	0.660
ALBI grade 2/3	2.00	0.82 - 4.89	0.129
ALBI score	1.27	0.51 - 3.15	0.608
ECOG-PS	0.59	0.22 - 1.54	0.280
AFP $\geq$ 400 ng/mL	1.35	0.55 - 3.36	0.512
Size of major nodule (cm)	0.99	0.93 - 1.06	0.877
Number of nodules > 3	0.79	0.31 - 1.99	0.614
Extrahepatic spread	1.07	0.41 - 2.76	0.887
Macrovascular invasion	1.51	0.58 - 3.96	0.398
BCLC stage C	0.92	0.38 - 2.24	0.853
STRIDE	0.49	0.08 - 2.84	0.425
