# Supplementary material for: Reproducible safety and efficacy of durvalumab with or without tremelimumab for hepatocellular carcinoma in clinical practice: Results of the DT-real study
Source: JHEP Rep. 2025 Nov 20;8(3):101685. doi: 10.1016/j.jhepr.2025.101685 (PMC12886069; doi:10.1016/j.jhepr.2025.101685)
Supplement: Multimedia component 4 [file mmc4.pdf]

Reproducible safety and efficacy of durvalumab with or without tremelimumab for hepatocellular carcinoma in clinical practice: Results of the DT-real study

Ciro Celsa[1,2], Tiziana Pressiani[3], Naoshi Nishida[4], Shadi Mohamad Chamseddine[5], Ashwini Arvind[6], Michael Li[7], Marta Fortuny[8,9,10], Najib Ben Khaled[11], Massimo Iavarone[12,13], Hidenori Toyoda[14], Ilario Giovanni Rapposelli[15], Andrea Casadei-Gardini[16], Caterina Vivaldi[17], Susanna Ulahannan[18], Haripriya Andanamala[18], Bernhard Scheiner[19], Matthias Pinter[19], Elena Orlandi[20], Claudia A.M. Fulgenzi[1], Giulia F. Manfredi[1,21], Pasquale Lombardi[1,22], Antonio D'Alessio[1], Bernardo Stefanini[23], Rosanna Villani[24], Francesca Romana Ponziani[25,26], Leonardo Stella[25], Ornella Carminati[27], Angela Dalia Ricci[28], Melina Gonzalez[8], Alba Sparacino[2], Gabriele Di Maria[2], Marco Vaccaro[2], Giuseppe Cabibbo[2], Calogero Cammà[2], Maria Reig[8,9,10,29], Robin K. Kelley[7], Amit G. Singal[6], Ahmed O. Kaseb[5], Masatoshi Kudo[4], Lorenza Rimassa[3,30], David James Pinato[1,21,*]

JHEP Reports **2026**. vol. 8 | 1–13

Check for updates

Background & Aims: Durvalumab plus tremelimumab (STRIDE) has emerged as a first-line systemic treatment option for unresectable hepatocellular carcinoma (HCC). This international multicentre study aimed to evaluate the efficacy and tolerability of STRIDE or durvalumab monotherapy in routine clinical practice, comparing outcomes between patients within and outside key eligibility criteria for the HIMALAYA trial.

Methods: From a database of 1,423 patients with advanced/unresectable HCC treated with immunotherapy across 35 centres, we analysed 233 patients receiving STRIDE or durvalumab monotherapy. Patients were categorized as HIMALAYA-IN or HIMALAYA-OUT based on key trial eligibility criteria (no prior systemic therapy, ECOG-PS 0–1, Child-Pugh class A, no Vp4 thrombosis). Baseline characteristics were assessed for overall survival (OS) and hepatic decompensation using a multivariable Cox model and competing-risk analysis, respectively. Objective response rates and treatment-related adverse events were recorded.

Results: Of the 233 patients, 123 (53%) were HIMALAYA-IN and 110 (47%) were HIMALAYA-OUT. STRIDE was given in 95% of HIMALAYA-IN patients. After median follow-up of 6.0 months, median OS was 20.4 months (95% CI 11.7-NR) in the overall population. HIMALAYA-IN patients achieved significantly longer OS than HIMALAYA-OUT patients (23.0 *vs.* 12.2 months; hazard ratio 0.61; 95% CI 0.39-0.96; p = 0.03). Macrovascular invasion and hepatic decompensation were independent negative prognostic factors in the whole cohort. Hepatic decompensation occurred in 10.5% of patients within 12 months from treatment start. Objective response rate was 23.7% and 17.8% of HIMALAYA-IN and -OUT patients, respectively. Patients achieving disease control (whole cohort: 59.4%) demonstrated 24-month OS of 58.2% in HIMALAYA-IN and 44.8% in HIMALAYA-OUT groups. Grade 3-4 treatment-related adverse events occurred in 16.3% of patients.

Conclusions: STRIDE shows reproducible effectiveness and an acceptable safety profile in real-world practice. Achieving disease control and maintaining liver function emerged as key determinants of long-term survival benefit.

© 2025 The Author(s). Published by Elsevier B.V. on behalf of European Association for the Study of the Liver (EASL). This is an open access article under the CC BY license (http://creativecommons.org/licenses/by/4.0/).

Introduction

In the landscape of unresectable/advanced hepatocellular carcinoma (HCC), combination immunotherapy has emerged as a significant therapeutic advancement over the past few years.[1] Following the publication of the IMbrave150 trial results,[2,3] which established atezolizumab plus bevacizumab as a new standard of care for first-line treatment of unresectable HCC, a number of randomised phase III trials have reported positive results *vs.* sorafenib or lenvatinib,[4,5] ushering in multiple immunotherapy-based regimens for frontline treatment of unresectable or metastatic disease.

The phase III HIMALAYA trial was the first to demonstrate the superiority of an anti-angiogenic-free regimen consisting of the anti PD-L1 antibody durvalumab plus a single priming dose of tremelimumab (an anti-CTLA-4 monoclonal antibody) (STRIDE regimen) over sorafenib in patients with unresectable HCC.[4] The STRIDE regimen led to a significant improvement in overall survival (OS), with a median OS of 16.4 months *vs.* 13.8 months for sorafenib, while maintaining a favourable toxicity profile. HIMALAYA is the first study to prospectively collect long-term OS data at clinically meaningful landmarks in a randomised controlled design. Exploratory analyses of the

* Corresponding author. Address: Imperial College London Hammersmith Campus, Du Cane Road, W12 0HS, London (UK); Tel: +44 0207 5942799.
E-mail address: david.pinato@imperial.ac.uk (D.J. Pinato).
https://doi.org/10.1016/j.jhepr.2025.101685

clinical trial dataset demonstrated a 60-month OS rate of approximately 20% for the STRIDE combination, suggesting an unprecedented long-term survival benefit in a significant proportion of patients.[6]

Whilst the readout of HIMALAYA led to a positive recommendation for STRIDE as a standard first-line option for patients with advanced HCC,[7] post-registration data on the tolerability and efficacy of this regimen are not yet available. As with any pivotal phase III trial meeting their primary endpoint, the stringent inclusion/exclusion criteria of HIMALAYA may limit the generalizability of its findings to the broader population encountered in routine clinical practice.

In the case of HIMALAYA, the study enrolled patients with preserved liver function, good performance status, and no neoplastic invasion of the main portal vein trunk (Vp4). Clinical trials often under-represent patients with more advanced liver dysfunction, comorbidities, or other characteristics commonly seen in routine practice.[8] This is of major importance in HCC, a tumour that arises in most of cases on the background of chronic liver disease or cirrhosis, where survival is bidirectionally influenced by the progression of cancer and underlying liver dysfunction.

Little evidence exists to suggest whether the adoption of durvalumab plus tremelimumab in routine practice is characterized by similar effectiveness and safety compared to the reference clinical trial population.[9] A precise description of clinical outcomes among patients receiving this combination in real-world settings is currently lacking, particularly in Western populations, as the few published real-world studies have primarily been conducted in Asia.

Specifically, whether STRIDE has preserved efficacy in patients with Vp4 portal vein invasion, or those with liver dysfunction is unknown and this is being tested in an ongoing phase IIIb trial including patients with either Eastern Cooperative Oncology Group - performance status (ECOG-PS) 2 or Child-Pugh class B or Vp4.[10] In addition, the impact of dual checkpoint inhibitor therapy on the risk of hepatic decompensation has not been assessed in patients receiving STRIDE. This is particularly relevant given the strong association between hepatic decompensation and mortality in patients treated with atezolizumab plus bevacizumab, both in real-world practice and in a *post hoc* analysis of the IMbrave150 trial.[11,12]

In this international multicentre study, we portrayed clinical outcomes of patients treated with durvalumab with or without tremelimumab outside clinical trials, with particular focus on comparing oncological outcomes depending on whether key eligibility criteria for HIMALAYA were met (no previous systemic therapy, ECOG-PS 0-1, Child-Pugh class A, absence of main portal vein thrombosis) prior to treatment initiation.

Patients and methods

Study design and population

Within a prospectively maintained database including 1,423 patients with unresectable HCC treated with immunotherapy in 35 tertiary referral centres across Europe, the USA and Asia,[13] we analysed patients who started durvalumab with or without tremelimumab and received at least one treatment dose from January 2022 to February 2025. Patients treated before FDA approval (October 2022) received treatment off-label based on the results of the phase I/II study.[14] Data were retrieved through electronic medical record review by trained physicians at each participating centre and entered into a dedicated Redcap platform.

All patients received either durvalumab 1,500 mg plus a single priming dose of tremelimumab 300 mg followed by durvalumab 1,500 mg intravenously every 4 weeks (STRIDE regimen), or durvalumab monotherapy at 1,500 mg intravenously every 4 weeks until disease progression, loss of clinical benefit, deterioration of ECOG-PS, unacceptable toxicity, patient-requested discontinuation, loss to follow-up, or death, in accordance with routine clinical practice. Dose modifications or interruptions of either drug followed the summary of product characteristics.

The primary inclusion criteria were: (1) histologically or radiologically confirmed diagnosis of HCC according to AASLD or EASL guidelines;[7,15] (2) unresectable or metastatic disease; (3) Child-Pugh score <10 points; (4) ECOG-PS 0-2. We collected data on all patients who received at least one dose of durvalumab with or without tremelimumab, regardless of whether they met the original HIMALAYA trial eligibility criteria.[4]

To compare outcomes based on adherence to key HIMALAYA trial eligibility, we categorized patients into two cohorts: (1) those who met key HIMALAYA trial eligibility criteria (no previous systemic therapy for HCC, Child-Pugh A, ECOG-PS 0-1, absence of main portal vein thrombosis [Vp4]), defined collectively as HIMALAYA-IN; and (2) those who failed to meet at least one of these criteria, defined as HIMALAYA-OUT.

Data collection

We collected the following baseline variables: age, sex, BMI, ECOG-PS, presence of type 2 diabetes, presence of cirrhosis, history of portal hypertensive bleeding, presence of gastroesophageal varices, ascites or hepatic encephalopathy, platelet count, albumin and bilirubin levels, international normalized ratio, Child-Pugh score, albumin-bilirubin (ALBI) grade, aetiology of chronic liver disease (viral *vs.* non-viral), Barcelona Clinic Liver Cancer (BCLC) stage, presence of neoplastic macrovascular invasion and its severity, presence of extrahepatic spread, alpha-fetoprotein (AFP) levels (<400 ng/ml *vs.* ≥400 ng/ml).

The diagnosis of MASLD (metabolic dysfunction-associated steatotic liver disease) was made in patients with steatotic liver disease associated with the presence of at least one cardiometabolic risk factor and self-reported alcohol intake of <20 g/day for women or <30 g/day for men.[16] Diagnosis of alcohol-associated liver disease was based on a history of self-reported regular alcohol intake of ≥20 g/day for women and ≥30 g/day for men, according to international guidelines.[17]

Response and outcome assessment

OS was defined as the time from treatment initiation to death from any cause. Progression-free survival (PFS) was defined as the time from treatment initiation to radiological progression or death from any cause. Patients who did not experience an event were censored at the date of last follow-up.

Radiological assessments were performed according to local standard of care, typically every 8-12 weeks. Response was evaluated using RECIST version 1.1, as assessed by local radiologists or investigators. Best overall response was classified as complete response (CR), partial response (PR), stable

disease (SD), or progressive disease (PD). Overall response rate (ORR) was defined as the proportion of patients achieving CR or PR as best response. Disease control rate (DCR) was defined as the proportion of patients achieving CR, PR, or SD.

Hepatic decompensation was defined according to international guidelines as the occurrence of new ascites, variceal bleeding, hepatic encephalopathy or jaundice[18] and it was assessed in patients without ascites or hepatic encephalopathy before treatment start. The occurrence of hepatic decompensation was verified by each investigator every 4 weeks through the review of medical history and physical examination.

Treatment duration was defined as the time from treatment initiation to the last dose. Treatment-related adverse events (TRAEs) were graded according to CTCAE version 5.0 by treating physicians. Treatment discontinuation for toxicity and the number of patients requiring high-dose systemic corticosteroid treatment for TRAEs (defined as a dose of prednisone or equivalents higher than 40 mg/daily) were also recorded.

Statistical analysis

Descriptive statistics were used to summarise baseline characteristics, treatment patterns, and safety data. Continuous variables were expressed as mean and standard deviation or median (IQR), and categorical variables were expressed as frequencies and percentages.

OS and PFS were estimated using the Kaplan-Meier method, with the log-rank test used to compare differences between groups.

The cumulative incidence function of hepatic decompensation was computed by competing risks analysis[19] in patients without ascites and/or encephalopathy before treatment start, with HCC progression and death in the absence of progression or decompensation as competing events, as previously reported.[11,12]

All outcomes of interest were compared between patients who met key HIMALAYA trial eligibility criteria (HIMALAYA-IN) and those who did not (HIMALAYA-OUT) and they were assessed in HIMALAYA-IN patients treated with STRIDE. As subgroup analyses, we compared OS in first-line treatment patients stratified by liver function (Child-Pugh A *vs.* B) and by the presence or absence of Vp4. Additionally, we conducted a comparative analysis of OS between patients treated with the STRIDE regimen *vs.* those who received durvalumab monotherapy.

In the overall population, in HIMALAYA-IN patients and in HIMALAYA-IN patients treated with STRIDE, multivariable Cox proportional hazards regression models were used to identify prognostic factors for OS and PFS. A multivariable Fine-Gray subdistribution hazard model was fitted to identify baseline predictors of hepatic decompensation in the whole cohort.[20] Multivariable logistic regression models were used to identify predictors of ORR and grade 3-4 TRAEs.

Variables with p values <0.10 in univariable analyses were included in the multivariable model. In the model for OS, we also assessed HCC radiological progression and hepatic decompensation occurring during treatment as time-dependent covariates in a Cox time-dependent model.[21] In the time-dependent analysis, patients with pre-treatment ascites and/or encephalopathy were considered as having hepatic decompensation at baseline.

As a supplementary analysis, we constructed directed acyclic graphs to assess whether this variable selection approach yielded results consistent with our primary analysis for OS.

OS analysis accounting for the time-dependent effect of radiological response was conducted using the Mantel-Byar method and Simon-Makuch plots.

A sensitivity analysis was conducted using the missRanger algorithm with predictive mean matching for missing data imputation of baseline variables.[22]

All statistical analyses were performed using the R-studio software, R Core Team (2021). R: A language and environment for statistical computing. R Foundation for Statistical Computing, Vienna, Austria. A two-sided p value <0.05 was considered statistically significant.

Ethical considerations

Ethical approval was granted by the Imperial College Tissue Bank (Reference Number R16008) and by local institutional review boards at each participating institution. The study was conducted in accordance with the Declaration of Helsinki and the International Conference on Harmonization Good Clinical Practice guidelines.

Results

Baseline characteristics

At the time of data cut-off (March 15, 2025), 237 patients from 16 centres were included in the dataset. After removing four patients not meeting the inclusion criteria, 233 patients were retained for analyses (Fig. S1).

Baseline characteristics of our cohort are summarised in Table 1. In brief, mean age was 69.4 years (±9.8) and most patients were male (n = 195, 84%) and had underlying cirrhosis (n = 179, 77%). The most common aetiologies of liver disease were alcohol-associated (n = 54, 23%) and HCV infection (n = 53, 23%), followed by MASLD (n = 49, 21%) and HBV (n = 32, 14%). While 163 (70%) patients had Child-Pugh class A liver disease, only 81 (35%) had ALBI grade 1. Gastroesophageal varices were present in 56 out of 126 patients (44%) with available upper gastrointestinal endoscopy data, with most of them having small varices (33/56, 59%). Median time from last endoscopy and the start of systemic treatment was 1.9 months (95% CI 1.4-2.9). ECOG-PS was 0 (n = 116, 50%), 1 (n = 63, 27%), or 2 (n = 54, 23%). Overall, the majority of patients had BCLC stage C HCC (n = 154, 66%), and 85 patients (36%) had baseline AFP ≥400 ng/ml.

Criteria for HIMALAYA eligibility (HIMALAYA-IN) were met in 123 (53%) patients, while at least one of these criteria was not met (HIMALAYA-OUT) in 110 (47%) patients. Most HIMALAYA-IN patients (n = 117/123, 95%) were treated with STRIDE. The most common reasons for exceeding trial eligibility criteria in HIMALAYA-OUT patients were Child-Pugh class B status (n = 70, 64%), ECOG-PS 2 (n = 54, 49%), treatment administration in second- or further-line (n = 47, 43%), and Vp4 portal invasion (n = 22, 20%). Among HIMALAYA-OUT, the four exclusion criteria were present in 6 patients (5.5%), while 19 (17%) and 27 patients (24.5%) scored positively for three and two exclusion criteria, respectively. The most common combinations were Child-Pugh B with ECOG-

Table 1. Description of baseline characteristics in the whole cohort and stratified by HIMALAYA eligibility.

	Overall (N = 233)	HIMALAYA-IN (n = 123, 52.8%)	HIMALAYA-OUT (n = 110, 47.2%)	*p* value
Age (years)	69.4 (9.8)	71.4 (8.5)	67.5 (10.7)	0.003
Male sex	195 (83.7)	105 (85.4)	90 (81.8)	0.579
BMI (kg/m^2)	25.8 (4.67)	26.2 (4.4)	25.4 (4.9)	0.238
Type 2 diabetes	51 (21.9)	31 (25.2)	20 (18.2)	0.612
Cirrhosis	179 (76.8)	82 (66.7)	97 (88.2)	<0.001
Aetiology				0.022
Alcohol	54 (23.2)	29 (23.6)	25 (22.7)	
HCV infection	53 (22.7)	35 (28.5)	18 (16.4)	
MASLD/MASH	49 (21.0)	22 (17.9)	27 (24.5)	
HBV infection	32 (13.7)	15 (12.2)	17 (15.5)	
Cryptogenic	18 (7.7)	14 (11.4)	4 (3.6)	
HCV-alcohol	18 (7.7)	5 (4.1)	13 (11.8)	
HCV-MASLD	5 (2.1)	2 (1.6)	3 (2.7)	
Others	4 (1.7)	2 (1.6)	2 (1.8)	
Previous portal hypertensive bleeding	12 (5.1)	4 (3.3)	8 (7.3)	0.113
Gastroesophageal varices	56 (44.4)	23 (31.9)	33 (61.1)	0.001
Not available	107 (45.9)	51 (41.5)	56 (50.9)	
Ascites	46 (19.7)	8 (6.5)	38 (34.5)	<0.001
Hepatic encephalopathy	11 (4.7)	0 (0)	11 (10)	<0.001
Platelet count (*10^9/L)	173 (98)	184 (96)	157 (100)	0.053
Albumin (g/dl)	3.7 (0.6)	3.9 (0.5)	3.4 (0.6)	<0.001
Bilirubin (mg/dl)	1.1 (1.3)	0.9 (0.5)	1.5 (1.9)	0.001
INR	1.2 (0.3)	1.1 (0.3)	1.2 (0.3)	0.003
Child-Pugh class A	163 (69.9)	123 (100)	40 (36.3)	<0.001
Child-Pugh score	6.1 (1.4)	5.3 (0.5)	7.0 (1.5)	<0.001
ALBI grade				<0.001
1	81 (34.8)	66 (53.6)	15 (13.6)	
2	139 (59.6)	56 (45.6)	83 (75.5)	
3	13 (5.6)	1 (0.8)	12 (10.9)	
ALBI score	-2.36 (0.63)	-2.60 (0.49)	-2.03 (0.65)	<0.001
ECOG-PS				<0.001
0	116 (49.8)	77 (62.6)	39 (35.4)	
1	63 (27.0)	46 (37.4)	17 (15.5)	
2	54 (23.2)	0 (0)	54 (49.1)	
AFP ≥400 ng/ml	85 (36.4)	41 (33.3)	44 (40.0)	0.432
Size of major nodule (cm)	6.2 (5.9)	6.6 (7.1)	5.8 (4.4)	0.336
Number of nodules				0.636
Single	54 (23.2)	27 (21.9)	27 (24.5)	
2-3	43 (18.5)	18 (14.6)	25 (22.7)	
>3	136 (58.3)	78 (63.4)	58 (52.7)	
Extrahepatic spread	71 (30.4)	37 (30.0)	34 (30.9)	0.365
Macrovascular invasion	72 (30.9)	31 (25.2)	41 (37.3)	0.071
Vp4 portal invasion	22 (9.4)	0 (0)	22 (20)	<0.001
BCLC stage				0.005
B	79 (33.9)	53 (43.1)	26 (23.6)	
C	154 (66.1)	70 (56.9)	84 (76.4)	
STRIDE	192 (82.4)	117 (95.1)	75 (68.2)	<0.001
Durvalumab monotherapy	41 (17.6)	6 (4.9)	35 (31.8)	
First-line treatment	186 (79.8)	123 (100)	63 (57.3)	

AFP, alpha-fetoprotein; ALBI, albumin-bilirubin; BCLC, Barcelona Clinic Liver Cancer; ECOG-PS, Eastern Cooperative Oncology Group performance status; INR, international normalised ratio; MASH, metabolic dysfunction-associated steatohepatitis; MASLD, metabolic dysfunction-associated steatotic liver disease.
Continuous variables are reported as mean values ± standard deviations. Categorical variables are reported as absolute numbers (percentage). Means were compared using Student's *t* test, and categorical variables were compared using the chi-square test.

PS 2 (41, 37%), Child-Pugh B with second- or further-line treatment (24, 22%) and Child-Pugh B with Vp4 (15, 14%). Among HIMALAYA-OUT patients, 75 patients (68%) received the STRIDE regimen, while 35 patients (32%) received durvalumab monotherapy.

Compared to HIMALAYA-OUT, HIMALAYA-IN patients were significantly older (71.4 *vs.* 67.5, p = 0.003), had a significantly lower prevalence of cirrhosis (67% *vs.* 88%, p <0.001) and gastroesophageal varices (32% *vs.* 61%, p = 0.001), and a significantly higher prevalence of HCV aetiology (28.5% *vs.* 16%, p = 0.022), ALBI grade 1 (54% *vs.* 17%, p <0.001) and BCLC stage B (43% *vs.* 24%, p = 0.005).

Proportions of missing variables are reported in Table S1, and they were below 10% for all variables.

Overall survival

After a median follow-up of 6.0 months (IQR 2.8-12.0), 82 patients (35.2%) were still receiving treatment at the time of database lock (March 15, 2025), 70 (30.0%) patients had discontinued treatment, and 81 (34.8%) had died.

Median OS was 20.4 months (95% CI 11.7–not reached [NR]) in the overall population. When comparing OS between HIMALAYA-IN and HIMALAYA-OUT patients, median OS was

23.0 months (95% CI 15.2–NR) in HIMALAYA-IN patients *vs.* 12.2 months (95% CI 8.0–20.4) in HIMALAYA-OUT patients (hazard ratio [HR] 0.61; 95% CI 0.39-0.96; p = 0.03) (Fig. 1A). 12-, 24- and 36-month OS rates in HIMALAYA-IN patients were 62.5% (95% CI 53.6-71.3%), 46.2% (95% CI 28.8-63.7%) and 33.0% (95% CI 3.4-62.6%).

In patients treated in the first-line setting (n = 186), median OS was 23.0 months (95% CI 15.2–NR) in Child-Pugh A patients and 11.1 months (95% CI 4.3–NR) in Child-Pugh B patients (HR for Child-Pugh B *vs.* A: 2.03, 95% CI 1.15-3.59, p = 0.02). In patients treated in the first-line setting, median OS was 22.8 months (95% CI 11.5-NR) in patients without Vp4 and was not reached (95% CI 5.36–NR) in patients with Vp4 (HR for Vp4 *vs.* no Vp4: 1.11, 95% CI 0.44–2.79, p = 0.82).

When analysing OS in HIMALAYA-OUT patients stratified by individual exclusion criteria, patients with Child-Pugh class B demonstrated significantly worse outcomes, with a median OS of 11.1 months (95% CI 4.3-20.4), while it was NR (95% CI NR–NR) in Child-Pugh class A patients (HR 2.29, 95% CI 1.23–4.25, p = 0.01). Patients with ECOG-PS >1 had a median

A

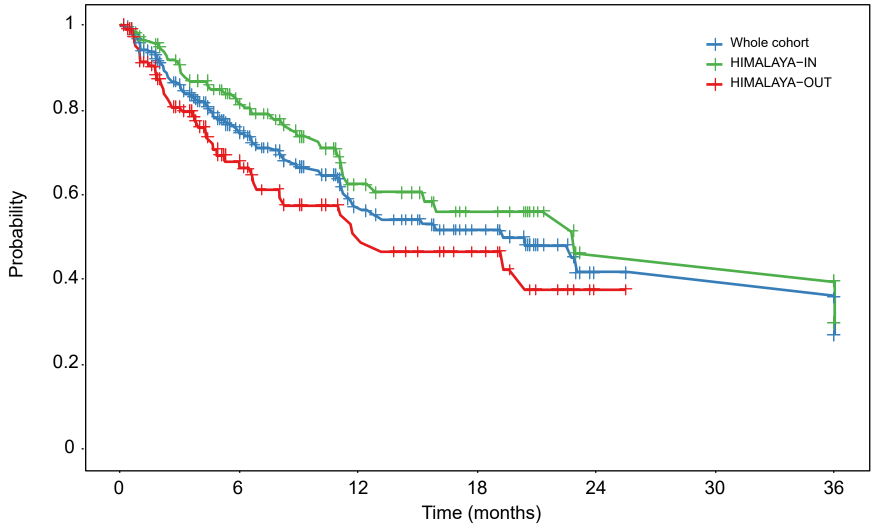

N° at risk							
Whole cohort	233	114	60	36	9	8	7
HIMALAYA−IN	123	71	36	22	8	8	7
HIMALAYA−OUT	110	44	24	15	2	0	0

B

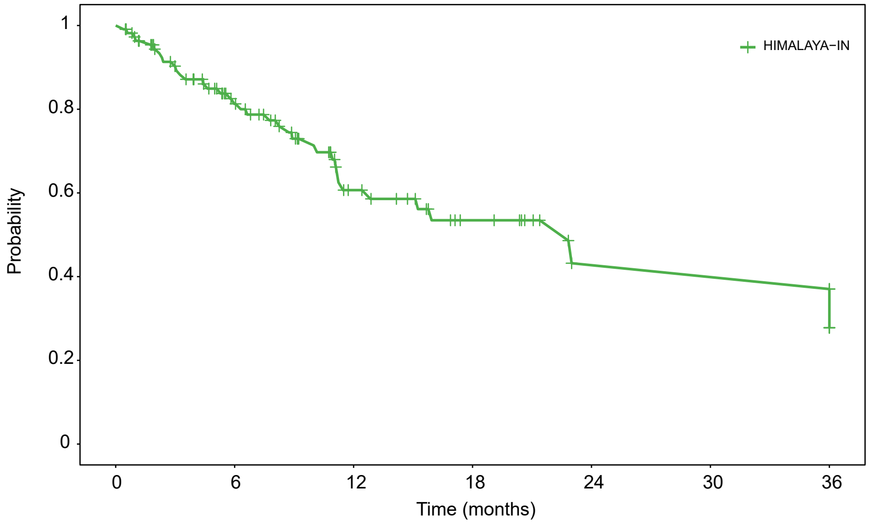

N° at risk							
HIMALAYA−IN	117	66	32	18	9	9	7

Fig. 1. Kaplan-Meier curves for overall survival. Kaplan-Meier curves for overall survival in the whole cohort and stratified by HIMALAYA eligibility (A) and in HIMALAYA-IN patients treated with STRIDE (B). Median overall survival was calculated by the Kaplan-Meier method. Median overall survival was 23.0 months (95% CI 15.2-NR) in HIMALAYA-IN patients *vs.* 12.2 months (95% CI 8.0-20.4) in HIMALAYA-OUT patients. The hazard ratio for HIMALAYA-IN *vs.* HIMALAYA-OUT was 0.61 (95% CI 0.39-0.96; p = 0.03) based on a Cox model. Median overall survival was 22.8 months (95% CI 11.5-NR) in HIMALAYA-IN patients treated with STRIDE. NR, not reached.

OS of 11.1 months (95% CI 4.9–20.4) *vs*. 13.2 months (95% CI 6.9–19.3) in those with ECOG-PS 0-1 (HR 1.33, 95% CI 0.71–2.50, p = 0.38). Patients with Vp4 showed a median OS of 12.2 months (95% CI 5.4–20.4) compared to 13.2 months (95% CI 6.7–19.3) in those without Vp4 (HR 1.01, 95% CI 0.49–2.13, p = 0.96). Finally, patients receiving second- or further-line treatment had a median OS of 19.3 months (95% CI 6.9-20.4) *vs*. 12.1 months (95% CI 4.9-12.1) in first-line patients (HR 0.78, 95% CI 0.42-1.44, p = 0.43).

Overall, median OS was 15.9 months (95% CI 11.5–NR) in patients treated with STRIDE and was not reached (95% CI 12.2–NR) in patients treated with durvalumab monotherapy (HR for STRIDE *vs.* durvalumab monotherapy: 1.15, 95% CI 0.61–2.16, p = 0.66). In HIMALAYA-IN patients treated with STRIDE, median OS was 22.8 months (95% CI 11.5–NR) (Fig. 1B) and was NR (95% CI NR–NR) in patients treated with durvalumab monotherapy (HR for STRIDE *vs.* durvalumab monotherapy: 0.49, 95% CI 0.14–1.61, p = 0.24). In HIMALAYA-OUT patients, median OS was 11.6 months (95% CI 6.7–19.3) in patients treated with STRIDE and 20.4 months (95% CI 4.7–20.4) in patients treated with durvalumab monotherapy (HR for STRIDE *vs*. durvalumab monotherapy: 1.05, 95% CI 0.54–2.03, p = 0.88).

In the whole cohort, median OS was not significantly different between patients with viral aetiology (15.2 months, 95% CI 11.1–20.4) and those with non-viral aetiology (22.8 months, 95% CI 11.2-NR) (HR 1.01, 95% CI 0.65–1.57, p = 0.96). Similarly, in HIMALAYA-IN patients, median OS was NR (95% CI NR–NR) in those with viral aetiology and 22.8 months (95% CI 11.1–NR) in those with non-viral aetiology (HR 0.77.95% CI 0.41-1.46, p = 0.43). In HIMALAYA-OUT patients, median OS was 11.1 months (95% CI 6.0–20.4) in those with viral aetiology and 19.3 months (95% CI 6.7–19.3) in those with non-viral aetiology (HR 1.33, 95% CI 0.72–2.45, p = 0.35).

Univariate Cox regression analyses are shown in Tables S2 and S3. As reported in Table 2, macrovascular invasion (HR 1.71, 95% CI 1.01–2.92, p = 0.048) and hepatic decompensation (HR 3.00, 95% CI 1.65–5.44, p <0.001) were independent prognostic factors for OS in the multivariate model. Similar results were obtained after imputation of missing data (Table S4).

In HIMALAYA-IN patients treated with STRIDE, age (HR 1.08, 95% CI 1.04–1.14, p <0.001), and Vp1-3 macrovascular invasion (HR 2.48, 95% CI 1.22-5.02, p = 0.01) were independent risk factors for mortality, while higher BMI was independently associated with a reduced risk of death (HR 0.93, 95% CI 0.87-0.99, p = 0.03) (Table 2). Directed acyclic graphs provided results that were consistent with our primary analysis both in the whole cohort (Fig. S2) and in HIMALAYA-IN patients (Fig. S3).

Progression-free survival

At the time of data cut-off, 132 (56.6%) patients had experienced progression or death. Median PFS was 6.0 months (95% CI 4.6–7.4) in the overall population. When comparing PFS between HIMALAYA-IN and HIMALAYA-OUT patients, median PFS was 6.6 months (95% CI 6.0–11.2) in HIMALAYA-IN patients *vs.* 3.9 months (95% CI 3.1–5.2) in HIMALAYA-OUT patients (HR 0.72; 95% CI 0.40–1.28; p = 0.26) (Fig. 2A).

PFS of HIMALAYA-OUT patients stratified according to exclusion criteria are reported in Table S5.

Table 2. Predictors of overall survival and progression-free survival by multivariable analysis in the whole cohort and in HIMALAYA-IN patients.

Overall survival											
**Whole cohort**				**HIMALAYA-IN patients**				**HIMALAYA-IN patients treated with STRIDE**			
**Covariates**	**Hazard ratio**	**95% CI**	***p* value**	**Covariates**	**Hazard ratio**	**95% CI**	***p* value**	**Covariates**	**Hazard ratio**	**95% CI**	***p* value**
**Overall survival**											
ALBI score	1.09	0.64 - 1.86	0.741	Age (years)	1.09	1.03 - 1.15	0.001	Age (years)	1.08	1.04-1.14	<0.001
Size of major nodule (cm)	1.04	0.99 - 1.09	0.124	BMI (kg/m^2)	0.90	0.84 - 0.97	0.007	BMI (kg/m^2)	0.93	0.87-0.99	0.028
Macrovascular invasion	1.71	1.01 - 2.92	0.048	Macrovascular invasion	2.30	1.09 - 4.88	0.029	Macrovascular invasion	2.48	1.22-5.02	0.012
ECOG-PS 2	1.14	0.59-2.19	0.692	Hepatic decompensation*	2.02	0.84 – 4.84	0.118	Hepatic decompensation*	1.56	0.79-3.08	0.385
Hepatic decompensation*	3.00	1.65 - 5.44	<0.001	-	-	-	-				
**Progression-free survival**											
ALBI score	1.37	0.98 - 1.92	0.062	Age (years)	1.08	1.04 - 1.12	<0.001	Age (years)**	1.08	1.04-1.12	<0.001
Number of nodules >3	1.39	0.96 - 2.02	0.084	BMI (kg/m^2)	0.94	0.89 - 0.99	0.037	-	-	-	-
Macrovascular invasion	1.27	0.86 - 1.89	0.236	-	-	-	-	-	-	-	-

ALBI, albumin-bilirubin; ECOG-PS, Eastern Cooperative Oncology Group performance status.
Hazard ratios and 95% CIs for overall survival and progression-free survival were calculated using Cox proportional hazards regression models.
*Considered as time-dependent covariate.
**Age was the only covariate significantly associated with progression-free survival in HIMALAYA-IN patients treated with STRIDE.

A

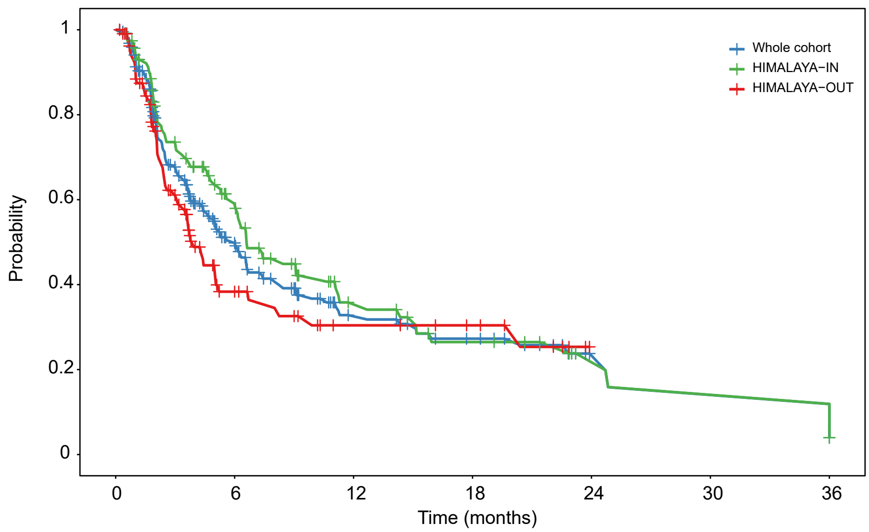

N° at risk							
Whole cohort	233	75	33	22	7	5	4
HIMALAYA−IN	123	53	22	14	7	5	4
HIMALAYA−OUT	110	23	12	9	0	0	0

B

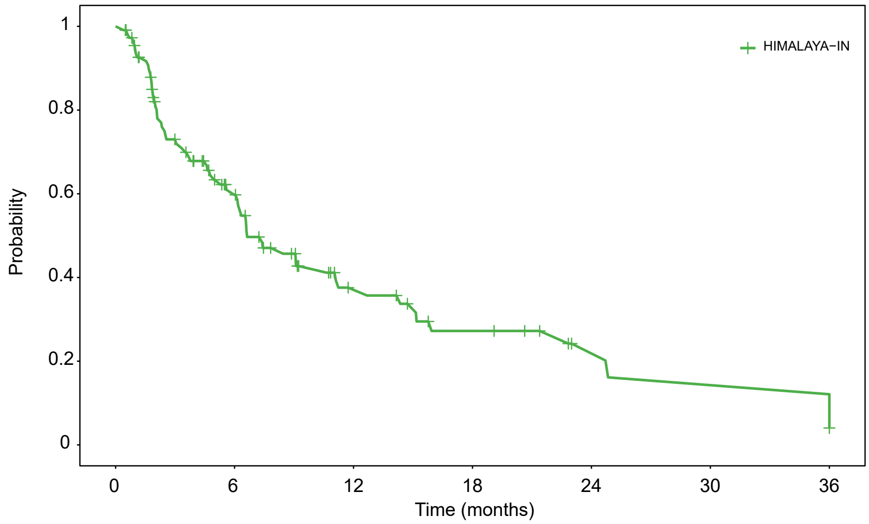

N° at risk							
HIMALAYA−IN	117	50	21	13	7	5	4

Fig. 2. Kaplan-Meier curves for progression-free survival. Kaplan-Meier curves for progression-free survival in the whole cohort and stratified by HIMALAYA eligibility (A) and in HIMALAYA-IN patients treated with STRIDE (B). Median progression-free survival was calculated by the Kaplan-Meier method. Median progression-free survival was 6.6 months (95% CI 6.0-11.2) in HIMALAYA-IN patients *vs.* 3.9 months (95% CI 3.1-5.2) in HIMALAYA-OUT patients. The hazard ratio for HIMALAYA-IN *vs.* HIMALAYA-OUT was 0.72 (95% CI 0.40-1.28; p = 0.26) based on a Cox model. Median progression-free survival was 6.6 months (95% CI 6.0-11.1) in HIMALAYA-IN patients treated with STRIDE.

Overall, median PFS was 6.3 months (95% CI 4.6–8.2) in patients treated with STRIDE and 4.4 (95% CI 2.5–11.3) in patients treated with durvalumab monotherapy (HR for STRIDE *vs.* durvalumab monotherapy: 0.88, 95% CI 0.56-1.41, p = 0.60). PFS for STRIDE and durvalumab monotherapy in HIMALAYA-IN and -OUT patients is reported in Table S6. In HIMALAYA-IN patients treated with STRIDE, median PFS was 6.6 months (95% CI 6.0–11.1) (Fig. 2B).

PFS stratified according to aetiology of liver disease is reported in Table S7.

Univariate Cox regression analyses are shown in Tables S8 and S9. As reported in Table 2, having more than 3 nodules (HR 1.39, 95% CI 0.96-2.02, p = 0.08) and ALBI score (considered as a continuous variable) (HR 1.37, 95% CI 0.98-1.92, p = 0.06) were associated with PFS in the multivariable model in the whole cohort; age (HR 1.08, 95% CI 1.04-1.12, p <0.001) and BMI (HR 0.94, 95% CI 0.89-0.99, p = 0.04) were independent prognostic factors for PFS in HIMALAYA-IN patients; and only age (HR 1.08, 95% CI 1.04-1.12, p <0.001) was significantly associated with PFS in HIMALAYA-IN patients treated with STRIDE.

Hepatic decompensation

At the time of data cut-off, 24 patients out of 185 patients without ascites and/or encephalopathy before treatment start

had experienced incident hepatic decompensation (13.0%), with ascites as the most common event (n = 19). Among 24 patients developing hepatic decompensation, 8 patients (8/24, 33.3%) developed hepatic decompensation in the absence of HCC progression, 10 patients (10/24, 41.7%) developed hepatic decompensation after HCC radiological progression (median time between progression and decompensation 1.1 months, range 0-8.8 months) and 6 patients (6/24, 25%) developed hepatic decompensation before HCC radiological progression (median time between decompensation and progression 1.1 months, range 0-5.3 months).

Rates of hepatic decompensation were 10.5% (95% CI 6.3-15.8%) at 6 and 12 months by competing risks analysis (Fig. S4).

The cumulative incidence of hepatic deceompensation for HIMALAYA-IN patients was 10.1% (95% CI 5.1-17.0%) at both 6 and 12 months. For HIMALAYA-OUT, the incidence was 11.5% (95%CI 4.9-21.3%) at 6 and 12 months. Gray's test showed no significant difference between the groups (p = 0.79) (Fig. 3).

Competing risks analysis of predictors of hepatic decompensation in the whole cohort is shown in Table S10. In univariate analysis, ALBI score, the presence of more than 3 nodules, and major nodule size were associated with increased risk of hepatic decompensation; however, none of these variables remained significantly associated with hepatic decompensation in multivariate analysis.

Objective response

At the time of data cut-off, 187 (80.3%) patients were evaluable for radiologic response. According to RECIST v1.1, 40 patients (21.4%) achieved ORR, with 9 patients (4.8%) experiencing CR and 31 (16.6%) patients experiencing PR. A total of 71 (38.0%) patients achieved SD, resulting in a DCR of 59.4%.

In HIMALAYA-IN patients, the ORR was 23.7% (27/114 patients), with 6.1% achieving CR and 17.5% achieving PR; 41.2% achieved SD, resulting in a DCR of 64.9%. In HIMALAYA-OUT patients, the ORR was 17.8% (13/73 patients), with 2.7% achieving CR and 15.1% achieving PR; 32.9% achieved SD, resulting in a DCR of 50.7%.

ORRs of HIMALAYA-OUT patients stratified according to exclusion criteria are reported in Table S11. ORRs for STRIDE and durvalumab monotherapy are reported in Table S12. In HIMALAYA-IN patients treated with STRIDE, ORR was 24.8% (27/109). ORR stratified according to aetiology of liver disease is reported in Table S13.

Univariate and multivariable analyses of predictors of ORR are shown in Tables S14 and S15. Only AFP >400 ng/ml emerged as a significant predictor of ORR in the whole cohort, age was independently associated with lower probability of ORR in HIMALAYA-IN patients, by multivariable analysis.

Patients with disease control (CR, PR or SD) demonstrated 24-month OS rates of 51.3%, 58.2%, 58.3% and 44.8% in the whole cohort, HIMALAYA-IN, HIMALAYA-IN treated with STRIDE and HIMALAYA-OUT cohort, respectively, while patients without disease control showed 24-month OS rates of 31.5%, 39.5%, 33.5% and 37.2%, respectively (Fig. 4). Similar results were obtained when radiological response was modelled as a time-dependent variable: HR of DCR *vs.* PD was 0.54 (95% CI 0.34-0.84, p = 0.006). Simon-Makuch plots are shown in Figs. S5–S8 and Mantel-Byar test was significant (p <0.001) in the whole cohort and HIMALAYA-IN patients, confirming that patients achieving DCR had significantly longer OS.

Kaplan-Meier curves of OS stratified by ORR, SD and PD are shown in Figs. S9–S12.

Treatment-related adverse events

Overall, during a median treatment duration of 6.2 months (IQR 3.0-10.4), 179 patients (76.8%) developed at least one TRAE

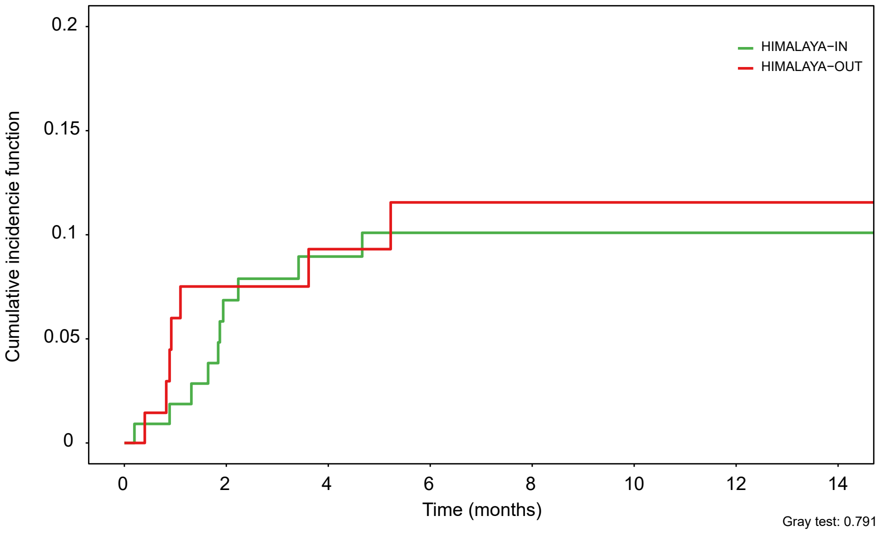

N° at risk	185	130	88	69	53	44	34	32
HIMALAYA−IN	0	7	9	10	10	10	10	10
HIMALAYA−OUT	0	5	6	7	7	7	7	7

Fig. 3. Cumulative incidence function of hepatic decompensation by competing risks analysis, stratified according to HIMALAYA eligibility. Curves were compared using Gray's test for competing risks. Gray's test showed no significant difference between the groups (p = 0.79).

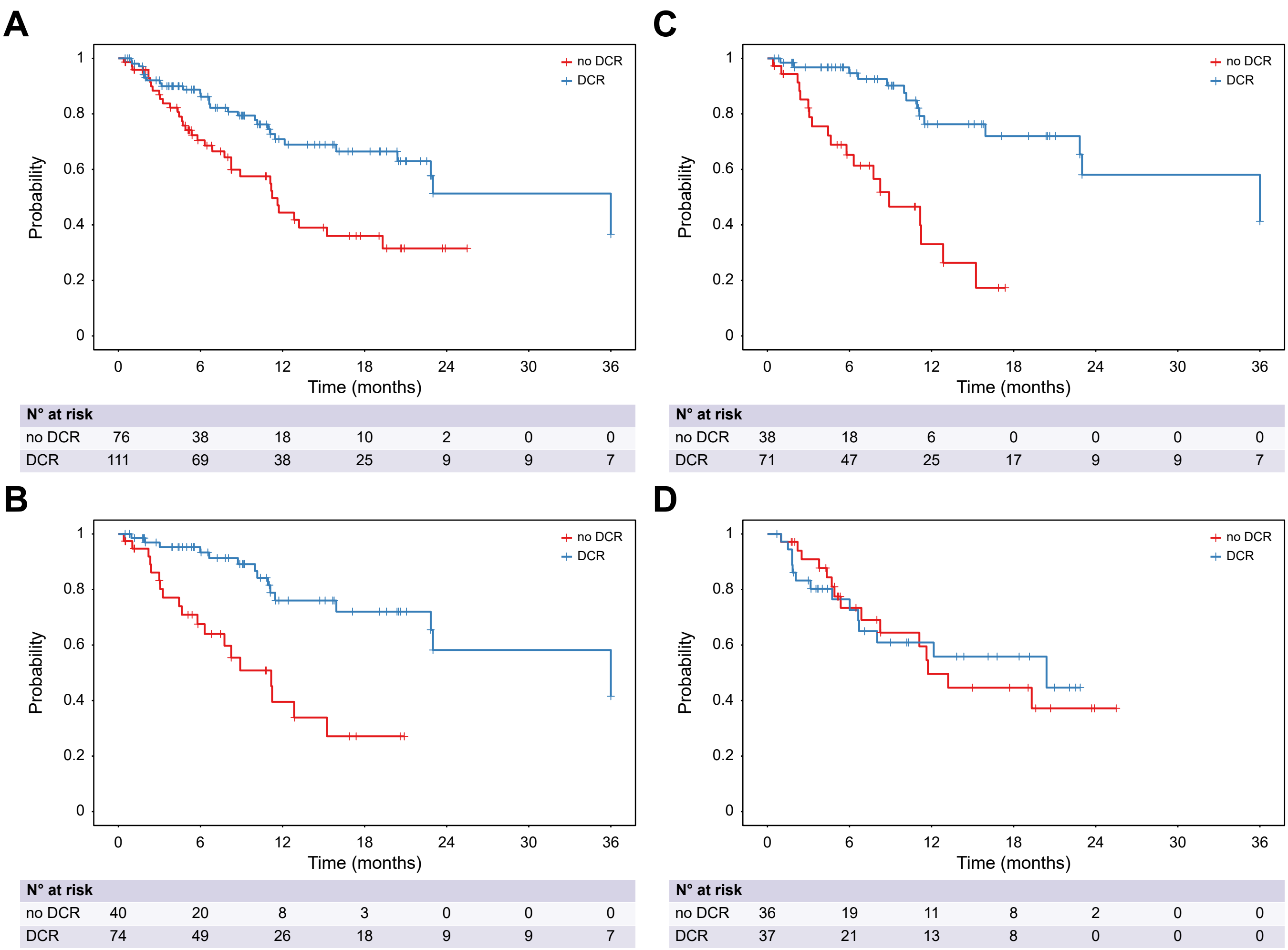

Fig. 4. Overall survival by disease control. Overall survival by disease control (yes/no) in the whole cohort (A), in HIMALAYA-IN (B), in HIMALAYA-IN treated with STRIDE (C) and in HIMALAYA-OUT patients (D). Kaplan-Meier method was used. Hazard ratio of disease control *vs.* no disease control was 0.54 (95% CI 0.34-0.84, p = 0.006), based on a time-dependent Cox model.

as per investigator assessment, with 38 individuals (16.3%) reporting grade 3 or 4 TRAEs. Overall, 12 patients (5.1%) permanently discontinued treatment due to TRAEs and 32 patients (13.7%) received high-dose systemic corticosteroids for treatment of immune-related adverse events.

As reported in Table S16, in the whole cohort, the most common TRAEs of any grade were skin toxicity (n = 35, 15.0%) and diarrhoea/colitis (n = 27, 11.6%), while diarrhoea/colitis and liver toxicity were the most common grade 3-4 AEs (n = 11, 4.7% and n = 9, 3.9%, respectively). Similar results were observed in HIMALAYA-IN patients, with skin toxicity and diarrhoea/colitis being the most common TRAEs of any grade (n = 25, 20.3% and n = 20, 16.3%, respectively), and diarrhoea/colitis and liver toxicity being the most common grade 3-4 AEs (n = 8, 6.5% and n = 5, 4.1%, respectively).

When comparing the safety profile between HIMALAYA-IN and HIMALAYA-OUT patients, the incidence of grade 3-4 TRAEs was 20.3% (n = 25) *vs.* 11.8% (n = 13)(p = 0.11), and the rate of treatment discontinuation due to TRAEs was 8.1% (n = 10) *vs.* 1.8% (n = 2)(p = 0.34), respectively.

Safety results of HIMALAYA-OUT patients stratified according to exclusion criteria are reported in Table S17.

When comparing the safety profile between STRIDE and durvalumab monotherapy in the whole cohort, the incidence of grade 3-4 TRAEs was 16.1% (n = 31) *vs.* 17.1% (n = 7) (p = 1.00), and the rate of treatment discontinuation due to TRAEs was 5.7% (n = 11) *vs.* 2.4% (n = 1) (p = 0.63), respectively. Safety results of STRIDE and durvalumab monotherapy in HIMALAYA-IN and -OUT patients are reported in Table S18. In HIMALAYA-IN patients treated with STRIDE, grade 3-4 TRAEs occurred in 19.7% (23/117).

Safety results stratified according to aetiology of liver disease are reported in Table S19.

Univariate and multivariable analyses of predictors of grade 3-4 TRAEs are shown in Tables S20 and S21. Child-Pugh class B was the only significant predictor of grade 3-4 TRAEs in the whole cohort (OR 0.38, 95% CI 0.15-0.96, p = 0.04). However, the significance of the association was not confirmed after adjusting for treatment duration (OR 0.48, 95% CI 0.19-1.22, p = 0.12). Age (OR 0.94, 0.89-0.99, p = 0.03) was the only significant predictor in HIMALAYA-IN patients.

Discussion

Combined inhibition of the CTLA-4 and PD-1/PD-L1 axes represents one of the first examples of a synergistic combination of immunotherapy agents in solid tumours.[23] Evolving experience with these agents suggests that they can induce a durable survival benefit in a subset of patients. Long-term follow-up of phase III studies in metastatic malignant melanoma suggests that more than 40% of patients are alive 10 years after dual checkpoint inhibition.[23]

The efficacy of dual CTLA-4/PD-L1 inhibition in unresectable/advanced HCC was first proven by the HIMALAYA study; however, durvalumab with or without tremelimumab has not been assessed outside clinical studies: a point of greater consequence given the relatively restrictive inclusion criteria of HIMALAYA compared to IMBrave150.

Our findings indicate that while the STRIDE regimen shows efficacy across the broader patient population, outcomes differ significantly between HIMALAYA-eligible and -ineligible patients, reflecting the importance of patient selection in treatment outcomes. While our overall median OS of 20.4 months appears favourable compared to the HIMALAYA trial, this comparison should be interpreted with caution as our cohort includes both HIMALAYA-eligible and ineligible patients, and treatment allocation was not randomised. The apparently favourable outcome may reflect patient selection or other unmeasured confounding factors, that we explored through several subgroup analyses.

The median OS of 23 months observed in our HIMALAYA-IN cohort compares favourably with the 16.4 months reported in the pivotal trial (4), suggesting robust real-world efficacy and external validity of the trial results. This improvement likely reflects factors related to patient selection and disease characteristics. Our HIMALAYA-IN cohort exhibited more favourable baseline tumour characteristics compared to the original trial population, including higher proportion of BCLC stage B disease (43.1% *vs*. 19.6% in the HIMALAYA trial) and lower prevalence of extrahepatic spread (30.0% *vs*. 53.2% in the HIMALAYA trial), indicating less advanced disease burden at treatment initiation. We believe these differences in baseline tumour burden may have influenced the outcomes observed in our study, as the remaining baseline characteristics of our patients were largely comparable to those reported in the HIMALAYA trial.

Notably, the 36-month OS rate of 33% that we report in the HIMALAYA-IN subset of patients indicates promising long-term survival benefit, and is similar to that observed in the HIMALAYA trial (30.7%).[6,24] However, this estimate is based on very limited follow-up (median, 6.0 months) and should be considered highly speculative, pending updated results with longer follow-up from our cohort.

Conversely, the significantly shorter OS of about 12 months in HIMALAYA-OUT patients underscores the impact of patient selection criteria in determining treatment outcomes. It was not surprising that baseline characteristics according to HIMALAYA trial eligibility were significantly different, with HIMALAYA-OUT patients being disfavoured in terms of severity of liver dysfunction and higher tumour burden.

Notably, our study represents a first analysis of this international database depicting the initial clinical uptake of durvalumab plus tremelimumab following regulatory approval. The fact that nearly half (47%) of our real-world cohort received treatment despite not meeting HIMALAYA trial eligibility criteria could reflect several factors. This off-label use likely stems from multidisciplinary tumour board discussions weighing the limited therapeutic alternatives for these patients, physician enthusiasm for a promising new therapy and different reimbursement frameworks across different healthcare systems that may not strictly enforce trial-based eligibility. Additionally, the unmet clinical need for effective systemic therapies in advanced HCC associated with Child-Pugh class B or after the failure of a first-line systemic treatment could have driven treatment decisions beyond the boundaries of the HIMALAYA trial.

Our multivariable analysis identified prognostic factors for OS that could be useful for patient stratification. In the overall cohort, macrovascular invasion and hepatic decompensation emerged as the strongest negative prognostic factors, and macrovascular invasion was confirmed as an independent risk factor for death in HIMALAYA-IN patients treated with STRIDE, together with age and BMI.

While we have demonstrated that macrovascular invasion was an independent predictor of worse outcomes both in the whole cohort and in the HIMALAYA-IN subgroup, we have also observed that OS was not significantly different between patients with and without Vp4 invasion. These findings suggest that although macrovascular invasion generally worsens prognosis, patients with Vp4 portal invasion may benefit from STRIDE therapy to the same extent as those with less severe grades of portal invasion. Noting that Vp4 invasion can induce portal hypertension and ensuing risk of varices and gastrointestinal bleeding, our data support that the STRIDE regimen is an appropriate choice of therapy for the Vp4 context, without the attendant bleeding risk conferred by other regimens with an anti-angiogenic component. However, this finding should be interpreted with extreme caution given the small sample size (n = 22) of patients with Vp4 and requires further confirmation from dedicated ongoing trials.[10]

The association between higher BMI and improved outcomes in patients with HCC treated with STRIDE may potentially relate to the so-called "obesity paradox," whereby factors potentially opposing the catabolic effects of cancer cachexia might lead to improved immune responsiveness.[25–27]

Our analysis did not reveal a significant impact of aetiology of underlying liver disease on OS, with efficacy appearing similar across different aetiologies. This finding aligns with different HIMALAYA sub-analyses,[28,29] supporting the broad applicability of STRIDE across different populations.

Alongside key host and tumour determinants of survival, the occurrence of hepatic decompensation had a strong impact on survival, highlighting its crucial prognostic significance. Decompensation represents the natural evolution of the disease in up to 10% of patients, a finding that aligns with previous observations in patients treated with atezolizumab plus bevacizumab, where hepatic decompensation was identified as a major determinant of mortality in both real-world and *post hoc* analyses of the IMbrave150 trial.[11,12] Our results extend this observation to durvalumab plus tremelimumab, suggesting that preservation of liver function during systemic therapy is a universal determinant of survival outcomes across different immunotherapy regimens. Accordingly, exploratory analyses from the HIMALAYA trial demonstrated that the benefit of

STRIDE over sorafenib was maintained regardless of ALBI grade, although patients with ALBI grade 1 had superior OS compared to those with ALBI grade 2/3.[30,31]

A finding that requires careful interpretation is the comparison between STRIDE and durvalumab monotherapy within patient subgroups. While no significant difference in OS was observed between the two regimens in HIMALAYA-IN patients, it should be noted that only a small proportion of patients (5%) received durvalumab monotherapy in this subgroup. Although OS was numerically longer in HIMALAYA-OUT patients treated with durvalumab monotherapy, the difference was not statistically significant. This observation should be interpreted with extreme caution due to the high potential for selection bias, given that physicians may have preferentially chosen monotherapy for patients perceived as having higher risk for toxicity from dual checkpoint inhibition, such as those with more advanced liver dysfunction or poorer performance status. Moreover, the sample size for monotherapy in HIMALAYA-OUT (n = 35) is too small to draw definitive conclusions about comparative effectiveness. Overall, we believe this finding is hypothesis-generating and warrants further investigation in appropriately designed prospective studies with propensity score techniques to account for treatment selection bias.

Although PFS was numerically longer in HIMALAYA-IN *vs.* HIMALAYA-OUT patients (6.6 *vs.* 3.9 months), it was not significantly different between the two groups. This finding suggests that while baseline characteristics impact OS substantially, their influence on disease progression may be less pronounced, potentially indicating that tumour biology rather than patient characteristics might be the primary driver of progression. Moreover, the role of PFS as a surrogate endpoint for OS remains questionable in patients treated with dual immunotherapy.[32] The decoupling of PFS from OS highlights the importance of evaluating long-term survival endpoints when assessing the efficacy of immunotherapy combinations in HCC[6] and emphasizes the need for more reliable intermediate endpoints that better capture the unique response patterns of immune checkpoint inhibitors in this disease.

The ORR of 23% in HIMALAYA-IN patients is similar to the 20.1% reported in the HIMALAYA trial. AFP ≥400 ng/ml emerged as a significant predictor of response in the overall cohort, suggesting that tumours with higher AFP expression might be more immunogenic and thus more responsive to immune checkpoint inhibition. Notably, disease control (CR, PR, or SD) was strongly associated with long-term survival outcomes across all patient groups. These findings are in line with those observed in the updated survival analysis of the HIMALAYA trial[6] and underscore the critical importance of achieving disease control as a predictor of long-term benefit.

The safety profile observed in our real-world cohort was generally consistent with that reported in the HIMALAYA trial and in exploratory analysis,[33] with skin toxicity and diarrhoea/colitis being the most common TRAEs. The incidence of grade 3-4 TRAEs (16.3% overall) was lower than the 25.4% reported in the HIMALAYA trial (4), potentially reflecting differences in adverse event reporting between clinical trials and real-world practice.

Whilst DT-real provides important confirmatory evidence of the efficacy and safety outcomes of STRIDE in patients with unresectable/advanced HCC, several limitations should be acknowledged. First, while our study represents a collaborative effort across 16 international centres, the final number of patients meeting HIMALAYA eligibility criteria was limited (n = 123), with only 6 patients in this subgroup receiving durvalumab monotherapy, precluding meaningful comparisons between STRIDE and monotherapy in this population. Second, the HIMALAYA-OUT category encompasses a heterogeneous group of patients with varying exclusion criteria. However, to address this heterogeneity, we have performed stratified outcome analyses based on the specific reason for HIMALAYA exclusion, which showed that only Child-Pugh class B was significantly associated with worse survival. Overall, all these subgroup analyses should be considered exploratory and require prospective validation in appropriately designed trials that are currently ongoing,[10] and the substantial heterogeneity within patient subgroups limits our ability to draw definitive conclusions about treatment effectiveness in specific clinical scenarios. Third, despite accurate attribution of decompensation *vs.* progression outcomes by experienced multidisciplinary teams in the context of high-volume referral centres for the care of HCC, decompensation and tumour progression often coexist and may be causally interlinked. Therefore, we cannot exclude that progression could have been the main determinant of decompensation in some patients or that radiological schedule of follow-up could have hampered the documentation of progression before the occurrence of decompensation. However, the inclusion of competing risk analyses for hepatic decompensation represents a methodological strength, accounting for the competing risk of tumour progression, as previously reported (11, 12). Additionally, the lack of systematic data on etiological treatment of cirrhosis and detailed tumour progression patterns precluded assessment of their potential prognostic impact on OS and hepatic decompensation. Fourth, the retrospective nature of our study introduces the potential for selection and reporting bias, as well as the potential for guarantee time bias. The relatively favourable outcomes observed even in HIMALAYA-OUT patients may reflect unmeasured selection factors, including referral patterns to tertiary centres, physician discretion in selecting patients perceived as having better outcomes, or other confounding variables not captured in our analysis. Finally, a major limitation of our study is the relatively short median follow-up of 6.0 months, which significantly limits the robustness of our OS analyses and long-term outcome assessments. While our 36-month OS rate of 33% in HIMALAYA-IN patients appears promising, this estimate is based on a limited number of patients at risk and should be interpreted with significant caution.

In conclusion, this international multicentre study demonstrates that STRIDE is an efficacious and tolerable treatment option in patients with unresectable/advanced HCC in routine clinical practice, with reproducible outcomes compared to reference level I evidence in those patients who would have met the HIMALAYA trial eligibility criteria. The significant disparity in outcomes between HIMALAYA-IN and HIMALAYA-OUT patients underscores the importance of careful patient selection and highlights the need for alternative strategies for patients with more advanced liver dysfunction or poorer performance status. Future prospective studies should focus on developing more refined predictive models to guide patient selection and on exploring strategies to extend the benefits of dual checkpoint inhibition to broader patient populations, while mitigating the risk of hepatic decompensation.

Affiliations

[1]Department of Surgery & Cancer, Imperial College London, Hammersmith Hospital, London, UK; [2]Gastroenterology and Hepatology Unit, Department of Health Promotion, Mother & Child Care, Internal Medicine & Medical Specialties, University of Palermo, Palermo, Italy; [3]Medical Oncology and Hematology Unit, Humanitas Cancer Center, IRCCS Humanitas Research Hospital, Rozzano, Milan, Italy; [4]Department of Gastroenterology and Hepatology, Kindai University, Osaka, Japan; [5]Department of Gastrointestinal Medical Oncology, M.D. Anderson Cancer Center, The University of Texas, Houston, TX, USA; [6]Division of Digestive and Liver Diseases, University of Texas Southwestern, Dallas, Texas, USA; [7]Division of Hematology/Oncology Helen Diller Family Comprehensive Cancer Center University of California, San Francisco San Francisco, California, USA; [8]Barcelona Clinic Liver Cancer (BCLC) Group, Institut d'Investigacions Biomèdiques August Pi i Sunyer (IDIBAPS), 08036 Barcelona, Spain; [9]Liver Oncology Unit, Liver Unit, Hospital Clinic de Barcelona, Barcelona, Spain; [10]Centro de Investigación Biomédica en Red en Enfermedades Hepáticas y Digestivas (CIBEREHD), Madrid, Spain; [11]Department of Medicine II, University Hospital, LMU Munich, Munich, Germany; [12]Division of Gastroenterology and Hepatology, Foundation IRCCS Ca' Granda Ospedale Maggiore Policlinico, Milan, Italy; [13]CRC "A. M. and A. Migliavacca" Center for Liver Disease, Department of Pathophysiology and Transplantation, University of Milan, Milan, Italy; [14]Department of Gastroenterology and Hepatology, Ogaki Municipal Hospital, Gifu, Japan; [15]Department of Medical Oncology, IRCCS Istituto Romagnolo per lo Studio dei Tumori (IRST) "Dino Amadori", Meldola, Italy; [16]Department of Oncology, Vita-Salute San Raffaele University, IRCCS San Raffaele Scientific Institute Hospital, Milan, Italy; [17]Unit of Medical Oncology 2, Azienda Ospedaliero- Universitaria Pisana, Pisa, Italy; [18]Stephenson Cancer Center, University of Oklahoma/ Oklahoma City, Oklahoma, USA; [19]Division of Gastroenterology and Hepatology, Department of Medicine III, Medical University of Vienna, Vienna, Austria; [20]Department of Oncology-Hematology, Azienda USL of Piacenza, Piacenza, Italy; [21]Department of Translational Medicine, University of Piemonte Orientale, Novara, Italy; [22]Phase 1 Unit, Fondazione Policlinico Universitario A. Gemelli, IRCCS, Università Cattolica del Sacro Cuore, Rome, Italy; [23]Department of Medical and Surgical Sciences, University of Bologna, Bologna, Italy; [24]Liver Unit, Department of Medical and Surgical Sciences, University of Foggia, Italy; [25]Liver Unit, CEMAD - Centro Malattie dell'Apparato Digerente, Medicina Interna e Gastroenterologia, Fondazione Policlinico Universitario Gemelli IRCCS, Rome, Italy; [26]Dipartimento di Medicina e Chirurgia Traslazionale, Università Cattolica del Sacro Cuore, Rome, Italy; [27]Medical Oncology Unit, AUSL della Romagna, Rimini Hospital, Rimini, Italy; [28]Medical Oncology Unit, National Institute of Gastroenterology, IRCCS "S. de Bellis" Research Hospital, Castellana Grotte, Italy; [29]Barcelona University, Barcelona, Spain; [30]Department of Biomedical Sciences, Humanitas University, Pieve Emanuele, Milan, Italy

Abbreviations

ALBI, albumin-bilirubin score; AFP, alpha-fetoprotein; BCLC, Barcelona Clinic Liver Cancer; CR, complete response; DCR, disease control rate; ECOG-PS, Eastern Cooperative Oncology Group – performance status; HCC, hepatocellular carcinoma; HR, hazard ratio; NR, not reached; OS, overall survival; ORR, overall response rate; PD, progressive disease; PR, partial response; PFS, progression-free survival; STRIDE, single tremelimumab regular interval durvalumab regimen; TRAEs, treatment-related adverse events; Vp4, main portal vein trunk invasion.

Financial support

Antonio D'Alessio is supported by the National Institute for Health Research (NIHR) Imperial BRC, by grant funding from the European Association for the Study of the Liver (Andrew Burroughs Fellowship) and from Cancer Research UK (RCCPDB-Nov21/100008). Amit G. Singal's research is supported in part by NIH R01 MD012565. Pasquale Lombardi is supported by European Society for Medical Oncology (ESMO) Translational Fellowship. Maria Reig is supported by Instituto de Salud Carlos III (PI18/0358 and PI22/01427), from Centro de Investigación Biomédica en Red CIBER (Immune4All, S2300092_3) and from the Spanish Association Against Cancer (AECC, PRYCO234831). Marta Fortuny is supported by Centro de Investigación Biomédica en Red CIBER (Immune4All) and from the Spanish Association Against Cancer (AECC, PRYCO234831). Calogero Cammà has received funding from the European Union - NextGenerationEU through the Italian Ministry of University and Research under PNRR M4C2I1.3 project PE_00000019 "HEAL ITALIA" CUPB73C22001250006. David J Pinato is supported by grant funding from the Cancer Treatment and Research Trust (CTRT), the Foundation for Liver Research and infrastructural support by the Imperial Experimental Cancer Medicine Centre and the NIHR Imperial Biomedical Research Centre. The views expressed are those of the authors and not necessarily those of the NHS, the NIHR, or the Department of Health and Social Care.

Conflicts of interest

CiC received speaker fees and advisory board honoraria from AstraZeneca, Eisai, Merck Sharp & Dohme, Ipsen and travel support from Roche. GC has served as a consultant or on advisory boards for Bayer, Eisai, Ipsen, MSD, AstraZeneca, Roche. AD received educational support for congress attendance from Roche and consultancy fees from Roche, AstraZeneca, Eisai, and Chugai. ACG reports consulting fees from AstraZeneca, Bayer, BMS, Eisai, Incyte, Ipsen, IQVIA, MSD, Roche, Servier; lecture fees from AstraZeneca, Bayer, BMS, Eisai, Incyte, Ipsen, Roche, Servier; travel expenses from AstraZeneca; research grants (to Institution) from AstraZeneca, Eisai. AGS has served as a consultant or on advisory boards for Genentech, AstraZeneca, Bayer, Eisai, Exelixis, Boston Scientific, Sirtex, FujiFilm Medical Sciences, Exact Sciences, Helio Genomics, Glycotest, DELFI, Freenome, ImCare, Curve Bio, Universal Dx. MF: Speaker fees from AstraZeneca. LR received consulting fees from AbbVie, AstraZeneca, Basilea, Bayer, BMS, Eisai, Elevar Therapeutics, Exelixis, Genenta, Hengrui, Incyte, Ipsen, Jazz Pharmaceuticals, MSD, Nerviano Medical Sciences, Roche, Servier, Taiho Oncology, Zymeworks; lecture fees from AstraZeneca, Bayer, BMS, Eisai, Guerbet, Incyte, Ipsen, Roche, Servier; travel expenses from AstraZeneca, Servier; and institutional research funding from AbbVie, AstraZeneca, BeiGene, Exelixis, Fibrogen, Incyte, Ipsen, Jazz Pharmaceuticals, MSD, Nerviano Medical Sciences, Roche, Servier, Taiho Oncology, ThansThera Sciences, Zymeworks. MI received speaker fees and advisory board honoraria from AstraZeneca, Roche, Roche Diagnostics, Eisai, Ipsen, MSD, Gilead. MP received speaker honoraria from AstraZeneca, Bayer, BMS, Eisai, Ipsen, Lilly, MSD, and Roche; he is a consultant/advisory board member for AstraZeneca, Bayer, BMS, Eisai, Ipsen, Lilly, MSD, and Roche; he received grants from AstraZeneca, Bayer, BMS, Eisai, and Roche; he received travel support from Bayer, BMS, Ipsen, and Roche. BS received travel support from Gilead, Ipsen and AbbVie. FRP received speaker fees, advisory board fees and travel grants from Bayer, MSD, Roche, Eisai, Ipsen, Astra-Zeneca, Gilead, Abbvie. CaC has served as a consultant or on advisory boards for Bayer, Eisai, Ipsen, MSD, AstraZeneca, Roche. MR: Consultant or Advisory Role: AstraZeneca, Bayer, BMS, Eli Lilly, Geneos, Ipsen, Merck, Roche, Universal DX, Boston Scientific, Engitix Therapeutics, Parabilis Medicines Inc; Speaking: AstraZeneca, Bayer, BMS, Eli Lilly, Gilead, Roche, Biotoscana Farma.; Travel support: Astrazeneca, Roche, Bayer, BMS, Lilly, Ipsen; Principal or sub-Investigator of drug under development: Abbvie, BMS, Adaptimmune, Nerviano, Medivir, Bayer, Ipsen, Astrazeneca, Terumo, Incyte, Roche, Boston Scientific, Medivir; Grant Research Support (to the institution): Bayer, Ipsen; Educational Support (to the institution): Bayer, Astrazeneca, Eisai-Merck MSD, Roche, Ipsen, Lilly, Terumo, BMS, Next, Boston Scientific, Ciscar Medical, Eventy C3 LLC (Egypt). RKK has served as a consultant or on advisory boards with funding to institution from Agios, Astra Zeneca, and Merck and to self from Compass, Elevar, GSK, Jazz, Moderna, Regeneron, Tyra Biosciences. She has received institutional research funding from Agios, Astra Zeneca, Bayer, BMS, Compass Therapeutics, Elevar, Eli Lilly, Exelixis, Genentech/Roche, Merck, Partner Therapeutics, QED, Relay Therapeutics, Servier, Taiho. DJP received lecture fees from ViiV Healthcare, Bayer Healthcare, BMS, Roche, Eisai, Falk Foundation, travel expenses from BMS and Bayer Healthcare; consulting fees for Mina Therapeutics, EISAI, Roche, DaVolterra, Mursla, Exact Sciences and Astra Zeneca; research funding (to institution) from MSD and BMS. All remaining authors have declared no conflicts of interest. The authors have no other relevant affiliations or financial involvement with any organization or entity with a financial interest in or financial conflict with the subject matter or materials discussed in the manuscript apart from those disclosed. No writing assistance was utilized in the production of this manuscript.
Please refer to the accompanying ICMJE disclosure forms for further details.

Authors' contributions

Study concept and design: CiC, DJP. Analysis and interpretation of data: CiC, GDM, MV. Statistical analyses: GDM, MV. Supervision: DJP. Drafting of the

manuscript: CiC, DJP. All the authors were involved in data acquisition and critical revision of the manuscript and they all approved the final version of the manuscript.

Data availability

Data, analytic methods and study materials will be made available to other researchers upon reasonable request.

Supplementary data

Supplementary data to this article can be found online at https://doi.org/10.1016/j.jhepr.2025.101685.

References

[1] Rimassa L, Finn RS, Sangro B. Combination immunotherapy for hepatocellular carcinoma. J Hepatol 2023;79:506–515.
[2] Finn RS, Qin S, Ikeda M, et al. Atezolizumab plus bevacizumab in unresectable hepatocellular carcinoma. N Engl J Med 2020;382:1894–1905.
[3] Cheng AL, Qin S, Ikeda M, et al. Updated efficacy and safety data from IMbrave150: atezolizumab plus bevacizumab *vs.* sorafenib for unresectable hepatocellular carcinoma. J Hepatol 2022;76:862–873.
[4] Abou-Alfa GK, Lau G, Kudo M, et al. Tremelimumab plus durvalumab in unresectable hepatocellular carcinoma. NEJM Evid 2022;1. EVIDoa2100070.
[5] Yau T, Galle P, Decaens T, et al. Nivolumab plus ipilimumab *vs.* lenvatinib or sorafenib as first-line treatment for unresectable hepatocellular carcinoma (CheckMate 9DW): a randomised, open-label, phase 3 trial. Lancet 2025 May 24;405(10492):1851–1864.
[6] Rimassa L, Chan SL, Sangro B, et al. Five-year overall survival update from the HIMALAYA study of tremelimumab plus durvalumab in unresectable HCC. J Hepatol 2025 Oct;83(4):899–908.
[7] European Association for the Study of the Liver. EASL Clinical Practice Guidelines on the management of hepatocellular carcinoma. J Hepatol 2025;82:315–374.
[8] Cabibbo G, Celsa C, Rimassa L, et al. Navigating the landscape of liver cancer management: study designs in clinical trials and clinical practice. J Hepatol 2024;80:957–966.
[9] Shimose S, Saeki I, Tomonari T, et al. Initial clinical experience with durvalumab plus tremelimumab in patients with unresectable hepatocellular carcinoma in real-world practice. Oncol Lett 2024;28:397.
[10] Chan S, Sangro B, Kudo M, et al. 206TiP SIERRA: a phase IIIb, single-arm, multicentre study of tremelimumab plus durvalumab for first-line treatment of advanced unresectable hepatocellular carcinoma. Ann Oncol 2023;34:S1554.
[11] Celsa C, Cabibbo G, Fulgenzi CAM, et al. Hepatic decompensation is the major driver of mortality in patients with HCC treated with atezolizumab plus bevacizumab: the impact of successful antiviral treatment. Hepatology 2025;81:837–852.
[12] Cabibbo G, Celsa C, Battaglia S, et al. Early hepatic decompensation identifies patients with hepatocellular carcinoma treated with atezolizumab plus bevacizumab or sorafenib at highest risk of death. Clin Cancer Res 2025;31:543–550.
[13] Fulgenzi CAM, Cheon J, D'Alessio A, et al. Reproducible safety and efficacy of atezolizumab plus bevacizumab for HCC in clinical practice: results of the AB-real study. Eur J Cancer 2022;175:204–213.
[14] Kelley RK, Sangro B, Harris W, et al. Safety, efficacy, and pharmacodynamics of tremelimumab plus durvalumab for patients with unresectable hepatocellular carcinoma: randomized expansion of a phase I/II study. J Clin Oncol 2021;39:2991–3001.
[15] European Association for the Study of the Liver. EASL clinical practice guidelines: management of hepatocellular carcinoma. J Hepatol 2018;69:182–236.
[16] Rinella ME, Lazarus JV, Ratziu V, et al. A multisociety Delphi consensus statement on new fatty liver disease nomenclature. J Hepatol 2023;79:1542–1556.
[17] European Association for the Study of the Liver. EASL clinical practice guidelines: management of alcohol-related liver disease. J Hepatol 2018;69:154–181.
[18] European Association for the Study of the Liver. EASL Clinical Practice Guidelines for the management of patients with decompensated cirrhosis. J Hepatol 2018;69:406–460.
[19] Putter H, Fiocco M, Geskus RB. Tutorial in biostatistics: competing risks and multi-state models. Stat Med 2007;26:2389–2430.
[20] Fine JP, Gray RJ. A proportional hazards model for the subdistribution of a competing risk. J Am Stat Assoc 1999;94:496–509.
[21] Klein JP, Moeschberger ML, SpringerLink. Survival analysis : techniques for censored and truncated data. New York, NY: Springer New York; 1997. Imprint: Springer.
[22] Mayer M, Mayer MM. Package 'missRanger'. R Package 2019;820.
[23] Wolchok JD, Chiarion-Sileni V, Rutkowski P, et al. Final, 10-year outcomes with nivolumab plus ipilimumab in advanced melanoma. N Engl J Med 2025;392:11–22.
[24] Sangro B, Chan SL, Kelley RK, et al. Four-year overall survival update from the phase III HIMALAYA study of tremelimumab plus durvalumab in unresectable hepatocellular carcinoma. Ann Oncol 2024;35:448–457.
[25] McQuade JL, Daniel CR, Hess KR, et al. Association of body-mass index and outcomes in patients with metastatic melanoma treated with targeted therapy, immunotherapy, or chemotherapy: a retrospective, multicohort analysis. Lancet Oncol 2018;19:310–322.
[26] Petrelli F, Cortellini A, Indini A, et al. Association of obesity with survival outcomes in patients with cancer: a systematic review and meta-analysis. JAMA Netw Open 2021;4:e213520.
[27] Cortellini A, Bersanelli M, Buti S, et al. A multicenter study of body mass index in cancer patients treated with anti-PD-1/PD-L1 immune checkpoint inhibitors: when overweight becomes favorable. J Immunother Cancer 2019;7:57.
[28] Reig M, Tam V, Sangro B, et al. 167P Non-viral aetiology subgroup of the phase III HIMALAYA study of tremelimumab (T) plus durvalumab (D) in unresectable hepatocellular carcinoma (uHCC). Ann Oncol 2024;35:S75–S76.
[29] Chan L, Kudo M, Sangro B, et al. 714P Impact of viral aetiology in the phase III HIMALAYA study of tremelimumab (T) plus durvalumab (D) in unresectable hepatocellular carcinoma (uHCC). Ann Oncol 2022;33:S869–S870.
[30] Kudo M, Rimassa L, Chan S, et al. 127O Five-year overall survival (OS) and OS by baseline liver function from the phase III HIMALAYA study of tremelimumab (T) plus durvalumab (D) in unresectable hepatocellular carcinoma (uHCC). Ann Oncol 2024;35:S1451–S1452.
[31] Vogel A, Chan S, Furuse J, et al. O-5 Outcomes by baseline liver function in patients with unresectable hepatocellular carcinoma treated with tremelimumab and durvalumab in the phase 3 HIMALAYA study. Ann Oncol 2022;33:S380–S381.
[32] Cabibbo G, Celsa C, Enea M, et al. Progression-free survival early assessment is a robust surrogate endpoint of overall survival in immunotherapy trials of hepatocellular carcinoma. Cancers (Basel) 2020;13.
[33] Lau G, Sangro B, Cheng AL, et al. Immune-mediated adverse events and overall survival with tremelimumab plus durvalumab and durvalumab monotherapy in unresectable hepatocellular carcinoma: HIMALAYA phase 3 randomized clinical trial. Hepatology 2025 May 16. https://doi.org/10.1097/HEP.0000000000001385.

Keywords: Hepatocellular carcinoma; Immunotherapy; Durvalumab; Tremelimumab; Real-world evidence.
Received 4 July 2025; received in revised form 5 November 2025; accepted 17 November 2025; Available online 20 November 2025

Journal of Hepatology, Volume *8*

Supplemental information

Reproducible safety and efficacy of durvalumab with or without tremelimumab for hepatocellular carcinoma in clinical practice: Results of the DT-real study

Ciro Celsa, Tiziana Pressiani, Naoshi Nishida, Shadi Mohamad Chamseddine, Ashwini Arvind, Michael Li, Marta Fortuny, Najib Ben Khaled, Massimo Iavarone, Hidenori Toyoda, Ilario Giovanni Rapposelli, Andrea Casadei-Gardini, Caterina Vivaldi, Susanna Ulahannan, Haripriya Andanamala, Bernhard Scheiner, Matthias Pinter, Elena Orlandi, Claudia A.M. Fulgenzi, Giulia F. Manfredi, Pasquale Lombardi, Antonio D'Alessio, Bernardo Stefanini, Rosanna Villani, Francesca Romana Ponziani, Leonardo Stella, Ornella Carminati, Angela Dalia Ricci, Melina Gonzalez, Alba Sparacino, Gabriele Di Maria, Marco Vaccaro, Giuseppe Cabibbo, Calogero Cammà, Maria Reig, Robin K. Kelley, Amit G. Singal, Ahmed O. Kaseb, Masatoshi Kudo, Lorenza Rimassa, and David James Pinato

Reproducible safety and efficacy of durvalumab with or without tremelimumab for hepatocellular carcinoma in clinical practice: Results of the DT-real study

Ciro Celsa, Tiziana Pressiani, Naoshi Nishida, Shadi Mohamad Chamseddine, Ashwini Arvind , Michael Li, Marta Fortuny-, Najib Ben Khaled, Massimo Iavarone, Hidenori Toyoda, Ilario Giovanni Rapposelli, Andrea Casadei-Gardini, Caterina Vivaldi, Susanna Ulahannan, Haripriya Andanamala, Bernhard Scheiner, Matthias Pinter, Elena Orlandi, Claudia A. M. Fulgenzi, Giulia F. Manfredi, Pasquale Lombardi, Antonio D'Alessio, Bernardo Stefanini, Rosanna Villani, Francesca Romana Ponziani, Leonardo Stella, Ornella Carminati, Angela Dalia Ricci, Melina Gonzalez, Alba Sparacino, Gabriele Di Maria, Marco Vaccaro, Giuseppe Cabibbo, Calogero Cammà, Maria Reig-, Robin K. Kelley, Amit G. Singal, Ahmed O. Kaseb, Masatoshi Kudo, Lorenza Rimassa, David James Pinato

Table of contents

Fig. S1 3
Fig. S2 4
Fig. S3 4
Fig. S4 5
Fig. S5 6
Fig. S6 7
Fig. S7 8
Fig. S8 9

Fig. S9 10
Fig. S10 11
Fig. S11 12
Fig. S12 13
Table S1 14
Table S2 15
Table S3 16
Table S4 17
Table S5 18
Table S6 18
Table S7 18
Table S8 19
Table S9 20
Table S10 21
Table S11 22
Table S12 22
Table S13 22
Table S14 23
Table S15 24
Table S16 25
Table S17 26
Table S18 26
Table S19 26
Table S20 27
Table S21 28

Fig. S1. Study flow-chart.

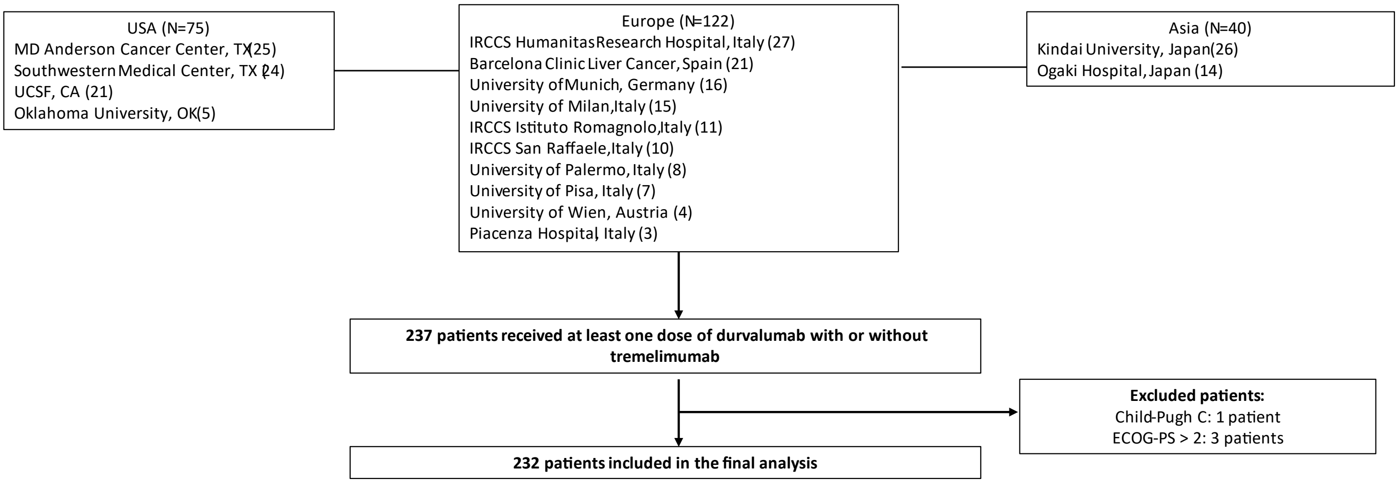

Fig. S2. Directed acyclic graph for overall survival in the whole cohort.

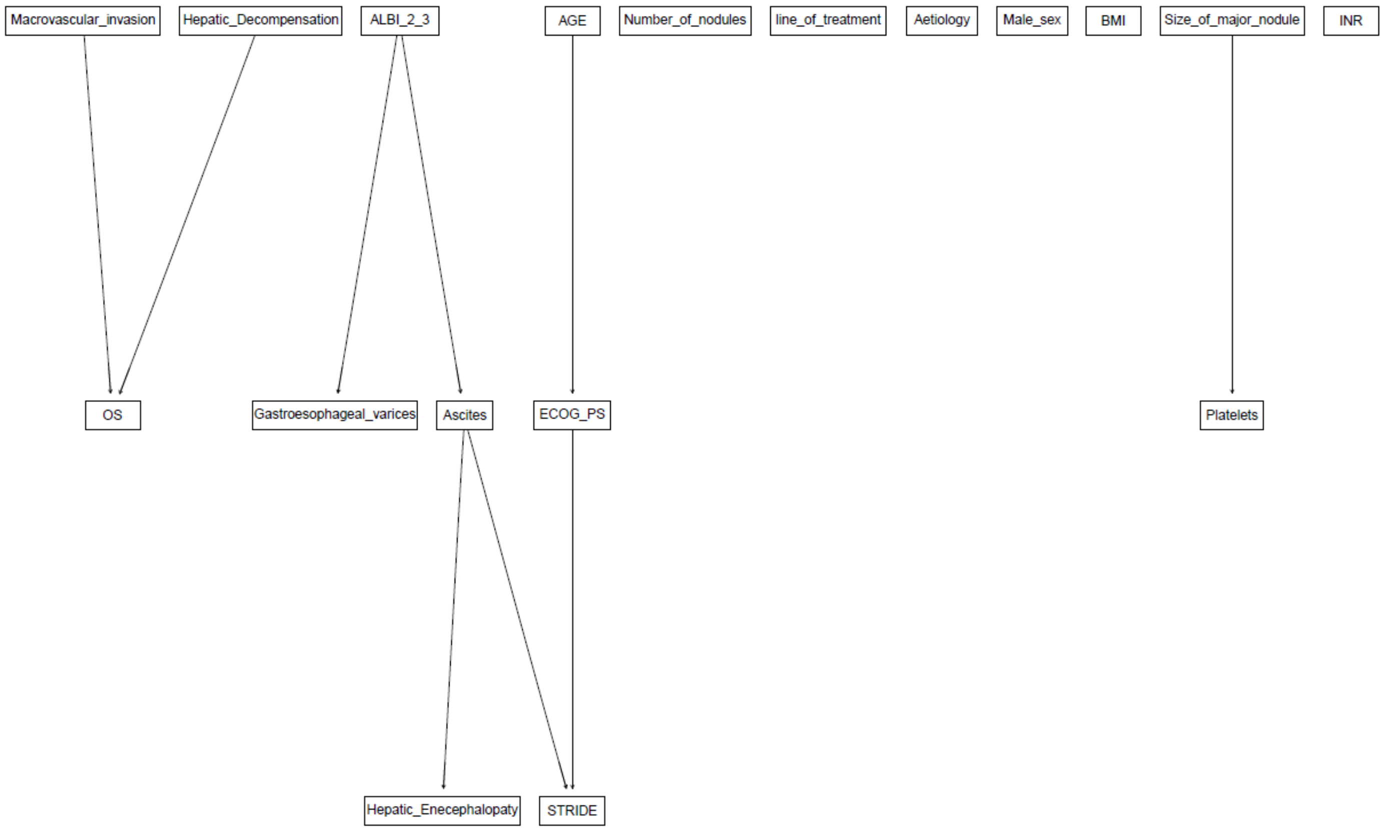

Fig. S3. Directed acyclic graph for overall survival in HIMALAYA-IN patients.

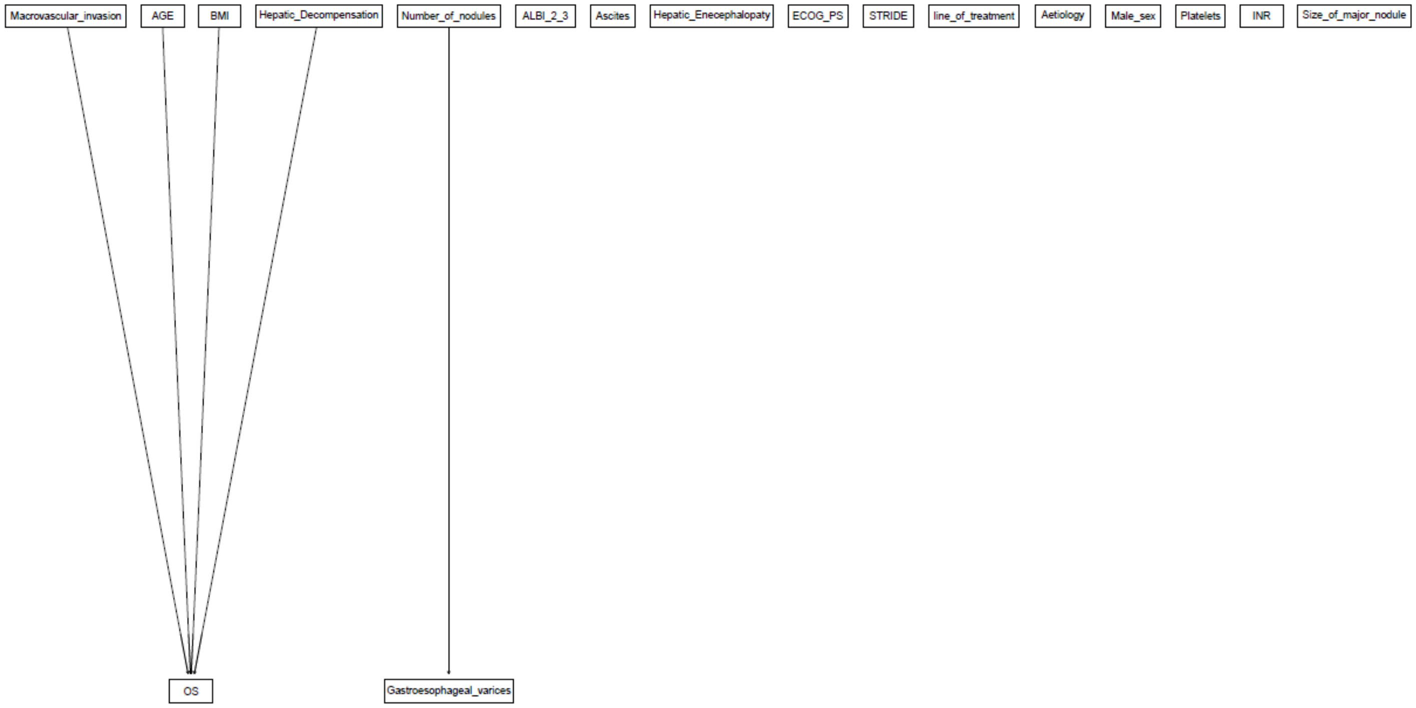

Fig. S4. Cumulative incidence function of hepatic decompensation in the whole cohort.

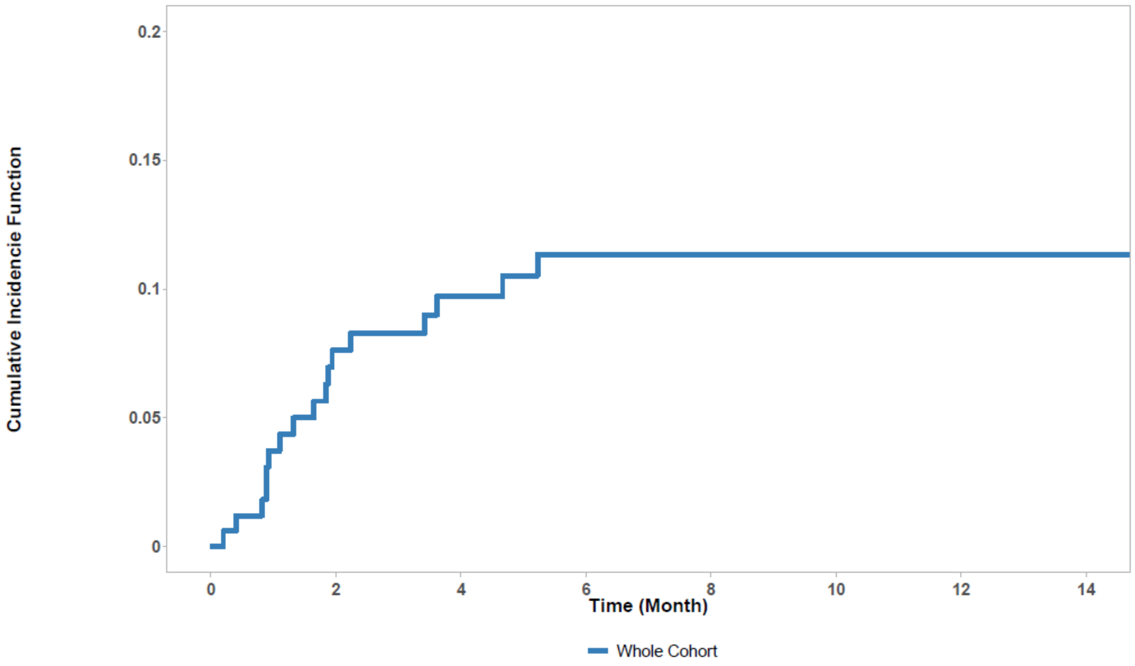

**N patients at risk**	185	133	92	73	58	49	40	39
**Whole Cohort**	0	12	15	17	17	17	17	17

Fig. S5. Simon-Makuch plot of overall survival by best radiological response (ORR vs SD vs PD) in the whole cohort.

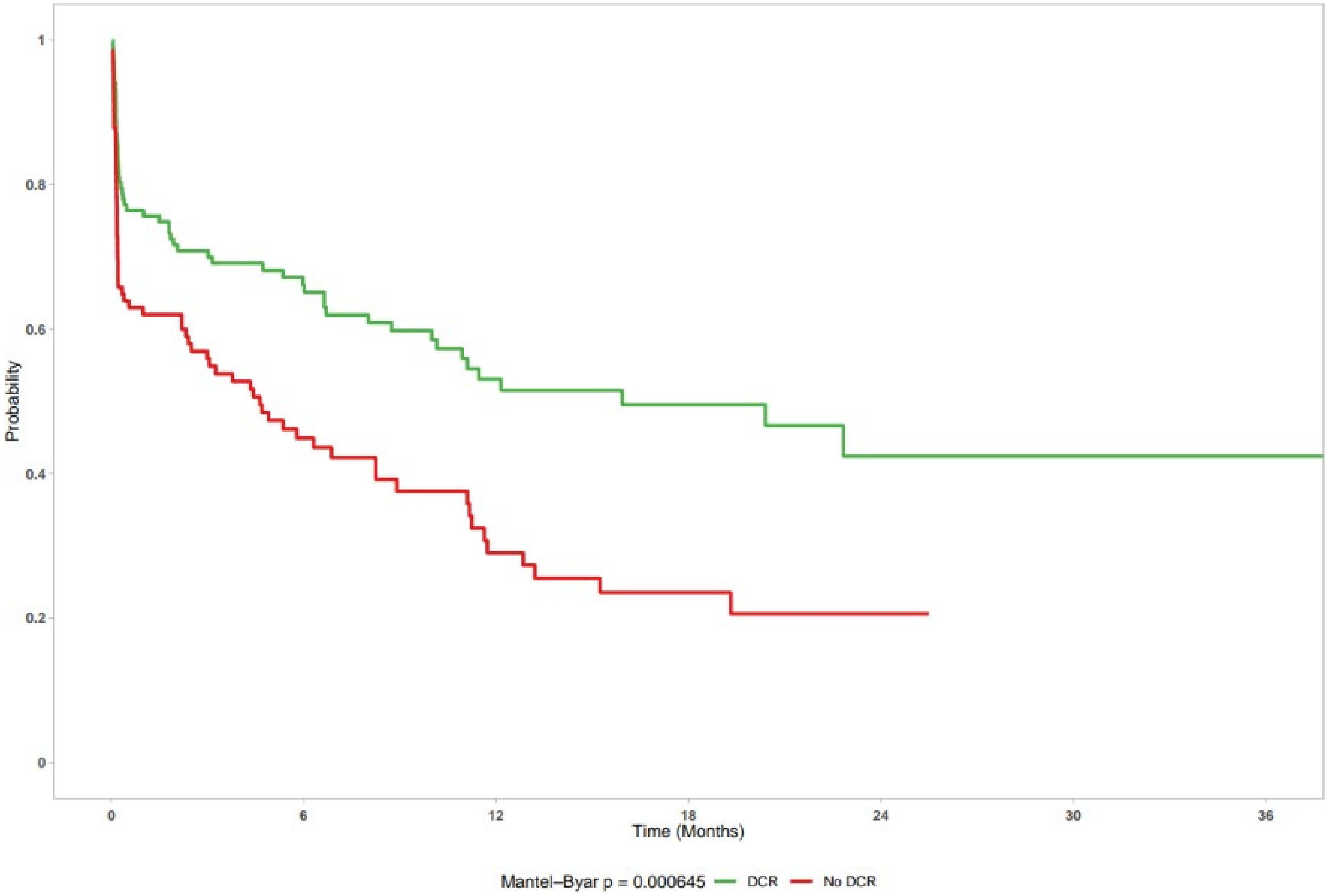

Fig. S6. Simon-Makuch plot of overall survival by best radiological response (ORR vs SD vs PD) in HIMALAYA-IN patients.

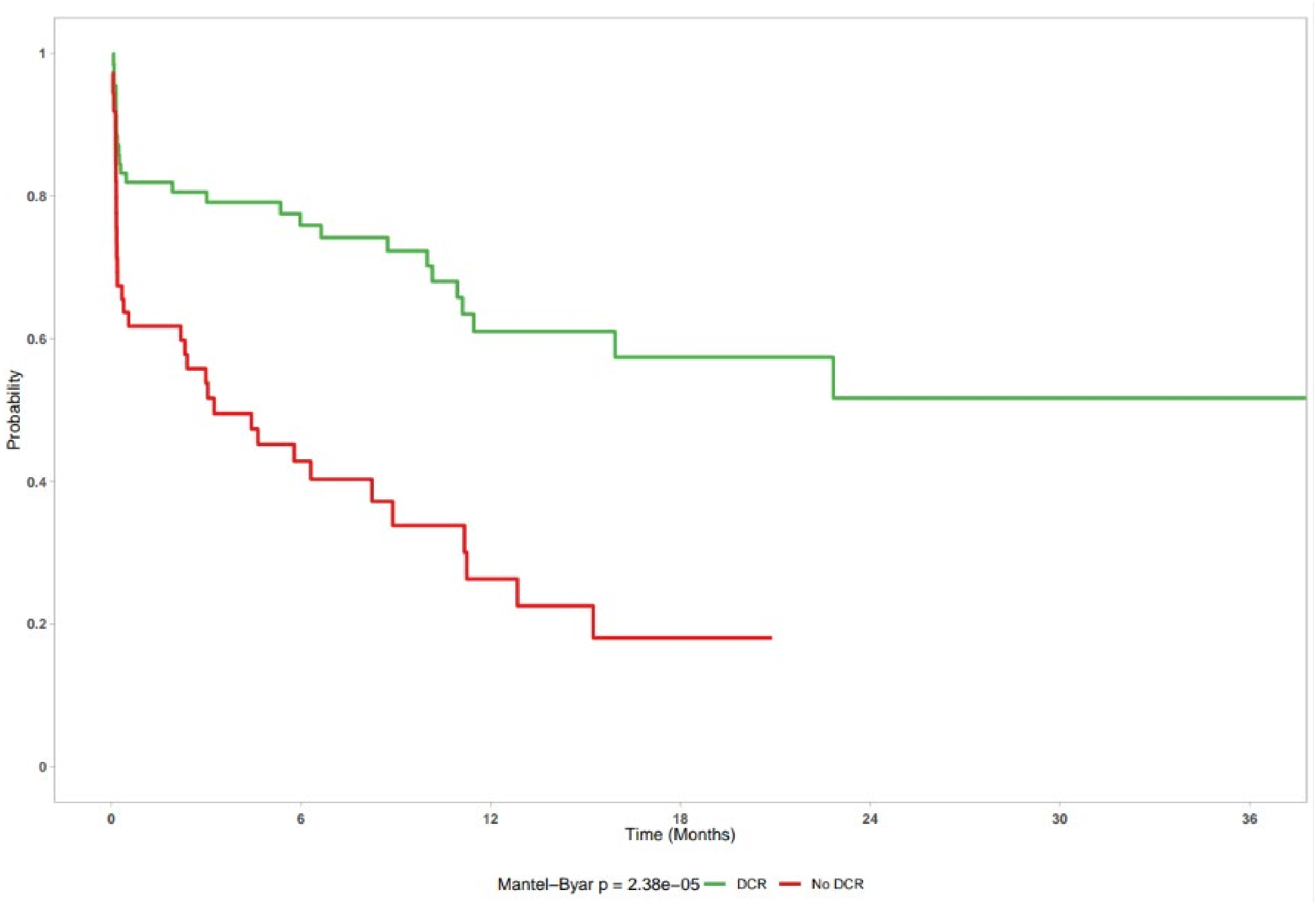

Fig. S7. Simon-Makuch plot of overall survival by best radiological response (ORR vs SD vs PD) in HIMALAYA-IN patients treated with STRIDE.

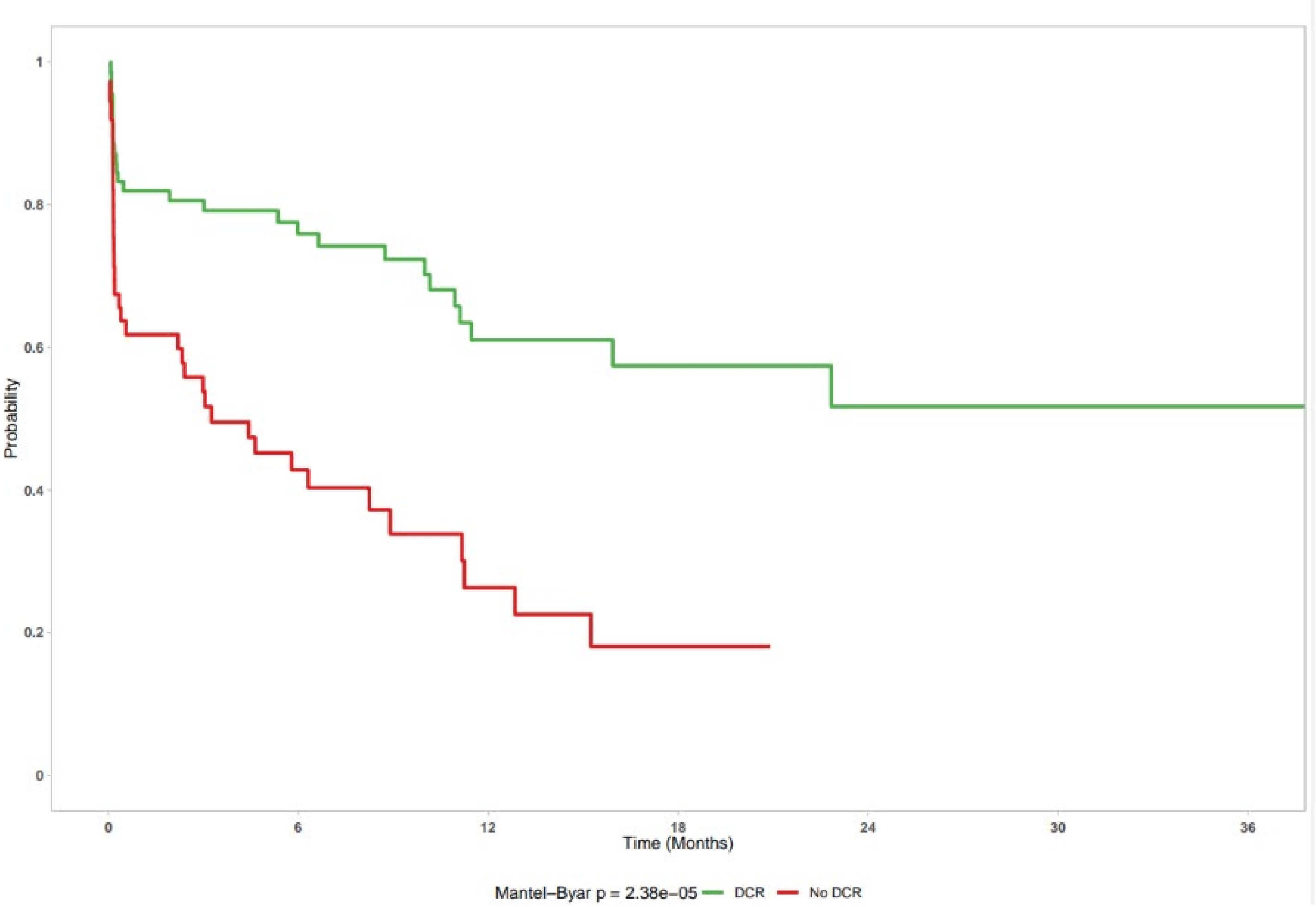

Fig. S8. Simon-Makuch plot of overall survival by best radiological response (ORR vs SD vs PD) in HIMALAYA-OUT patients.

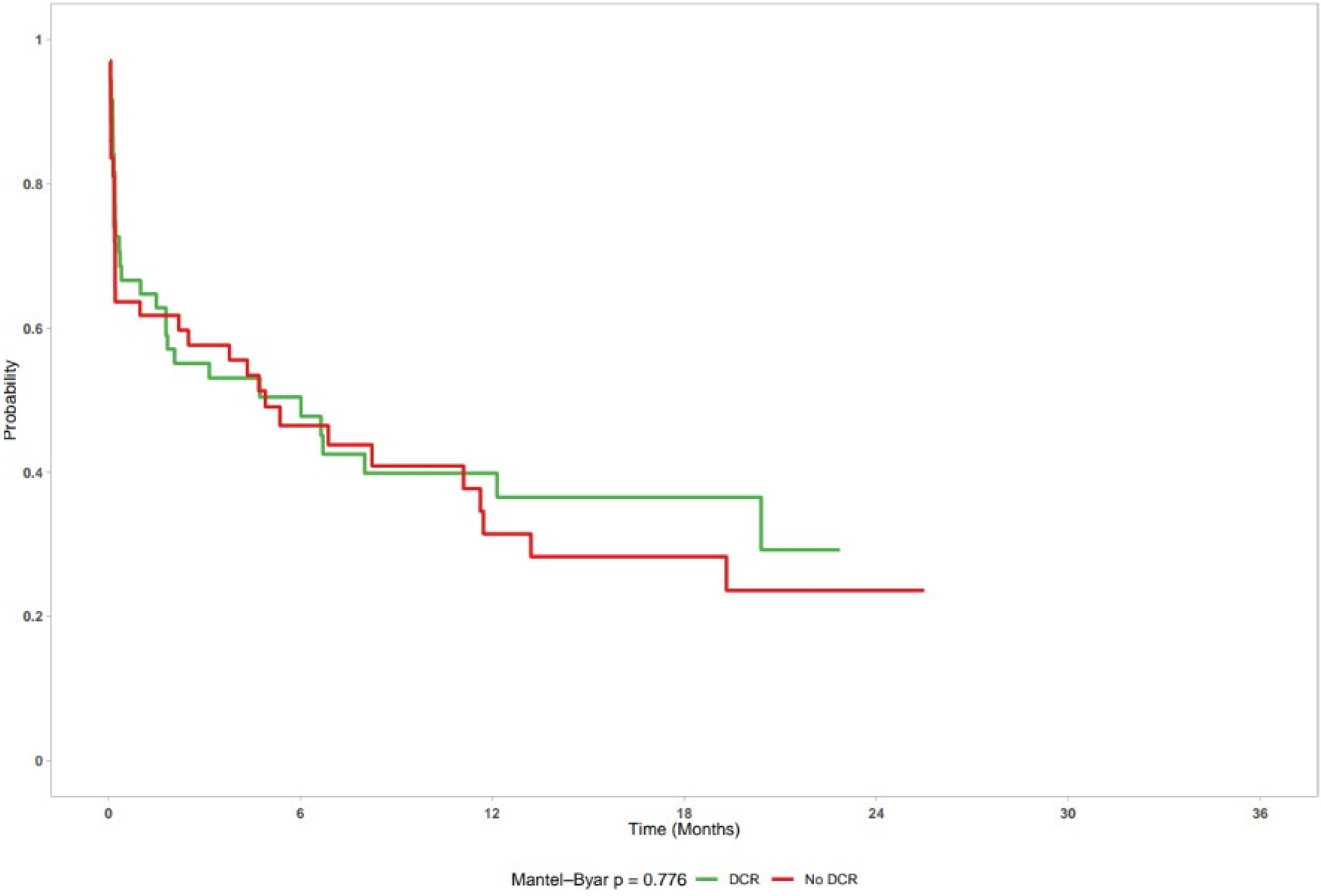

Fig. S9. Overall survival by best radiological response (ORR vs SD vs PD) in the whole cohort.

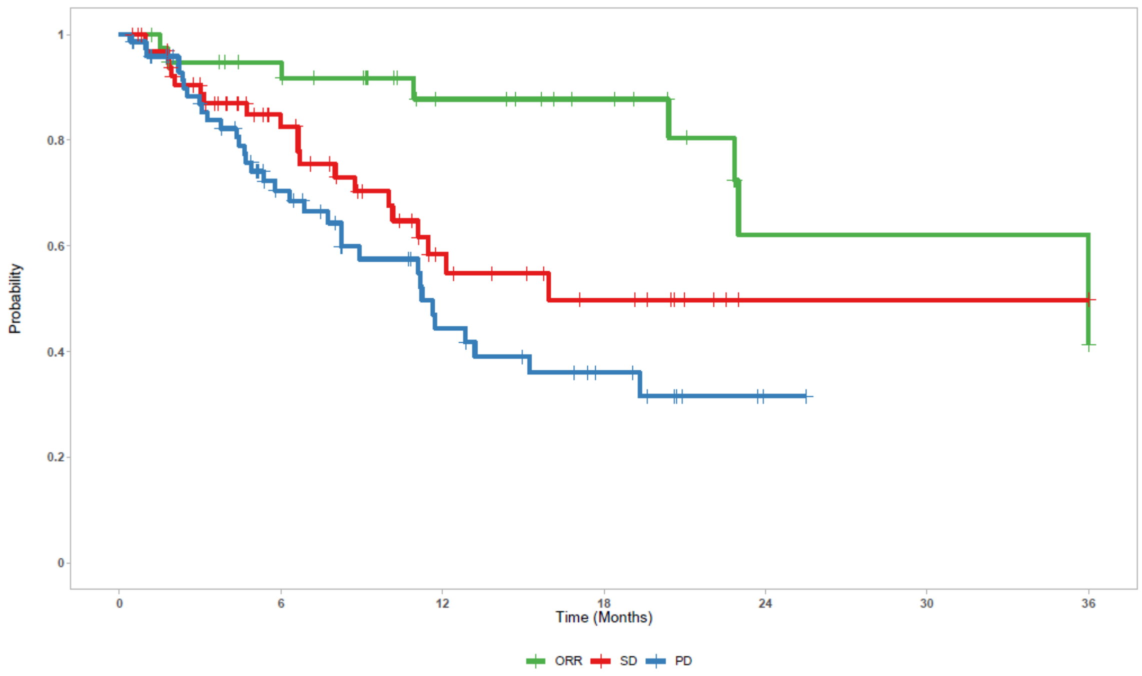

ORR	40	33	21	16	7	7	6
SD	71	37	17	10	2	2	1
PD	76	38	18	10	2	0	0

Fig. S10. Overall survival by best radiological response (ORR vs SD vs PD) in HIMALAYA IN patients.

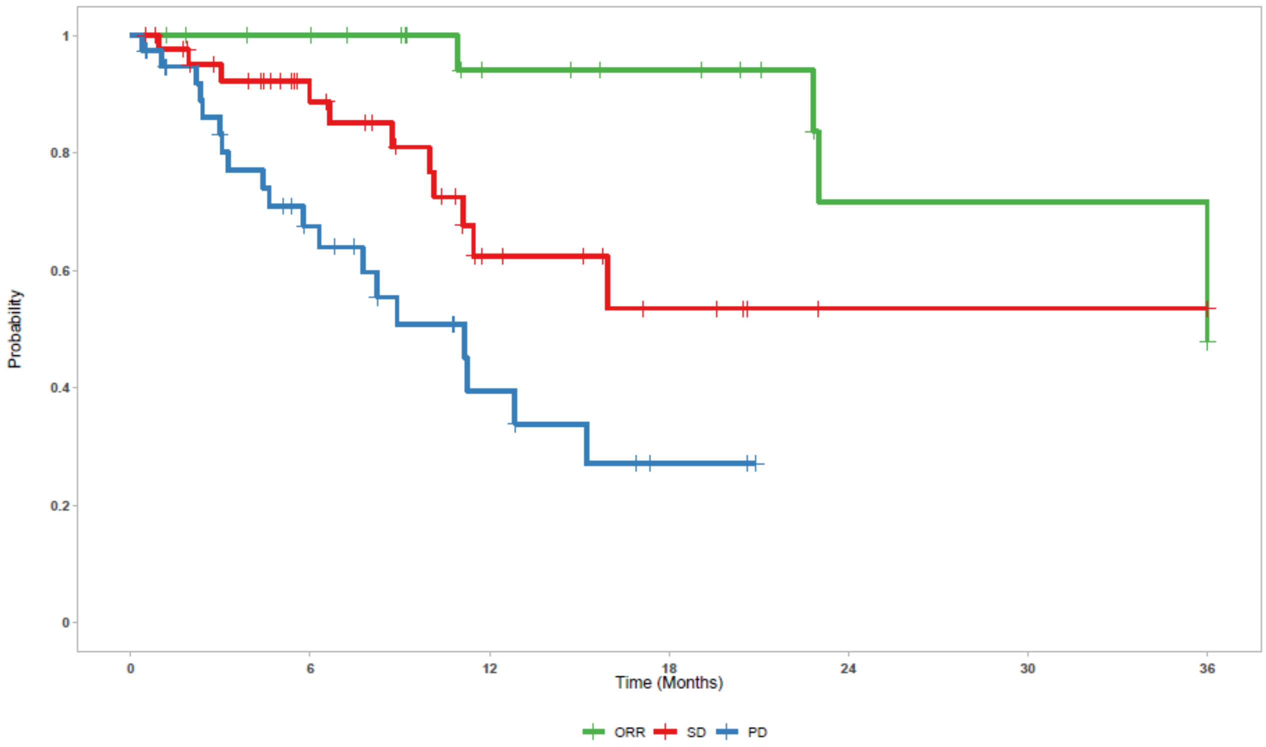

ORR	27	24	15	13	7	7	6
SD	47	26	11	6	2	2	1
PD	40	20	8	3	0	0	0

Fig. S11. Overall survival by best radiological response (ORR vs SD vs PD) in HIMALAYA IN patients treated with STRIDE.

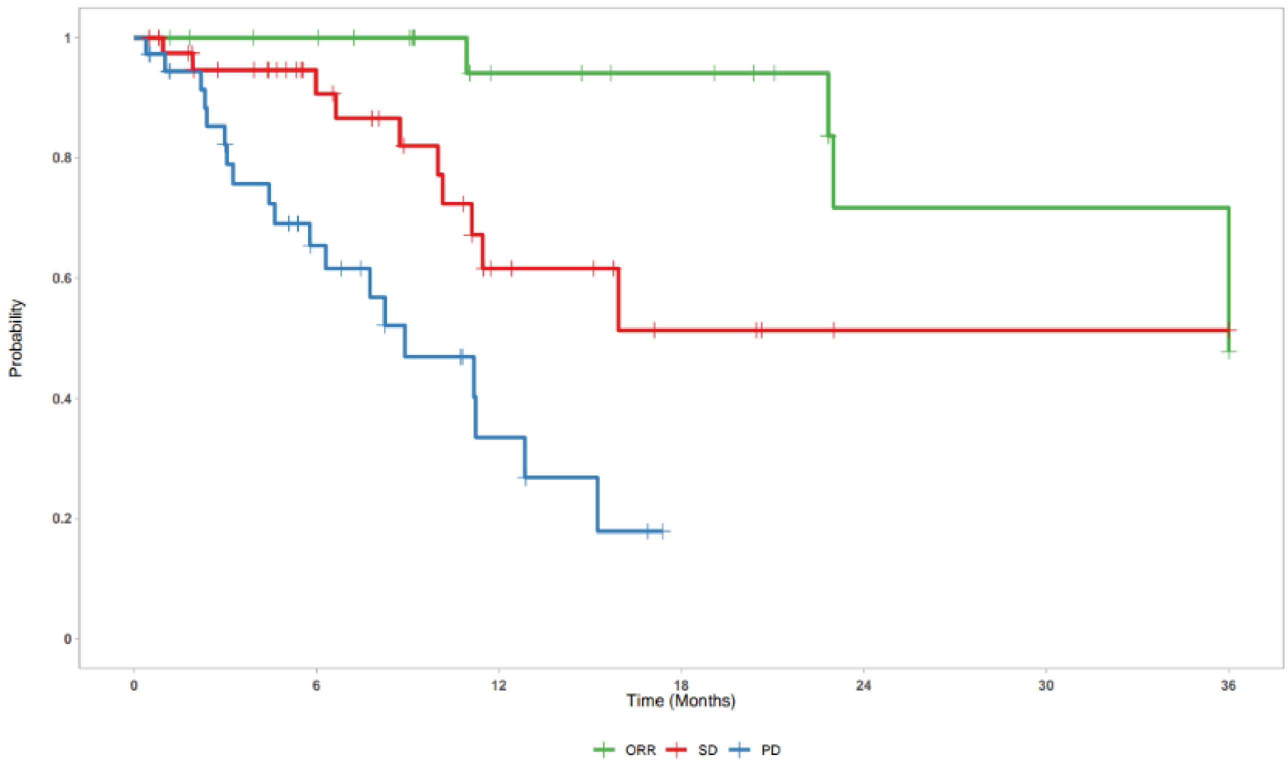

ORR	27	24	15	13	7	7	6
SD	44	24	10	5	2	2	1
PD	38	18	6	0	0	0	0

Fig. S12. Overall survival by best radiological response (ORR vs SD vs PD) in HIMALAYA OUT patients.

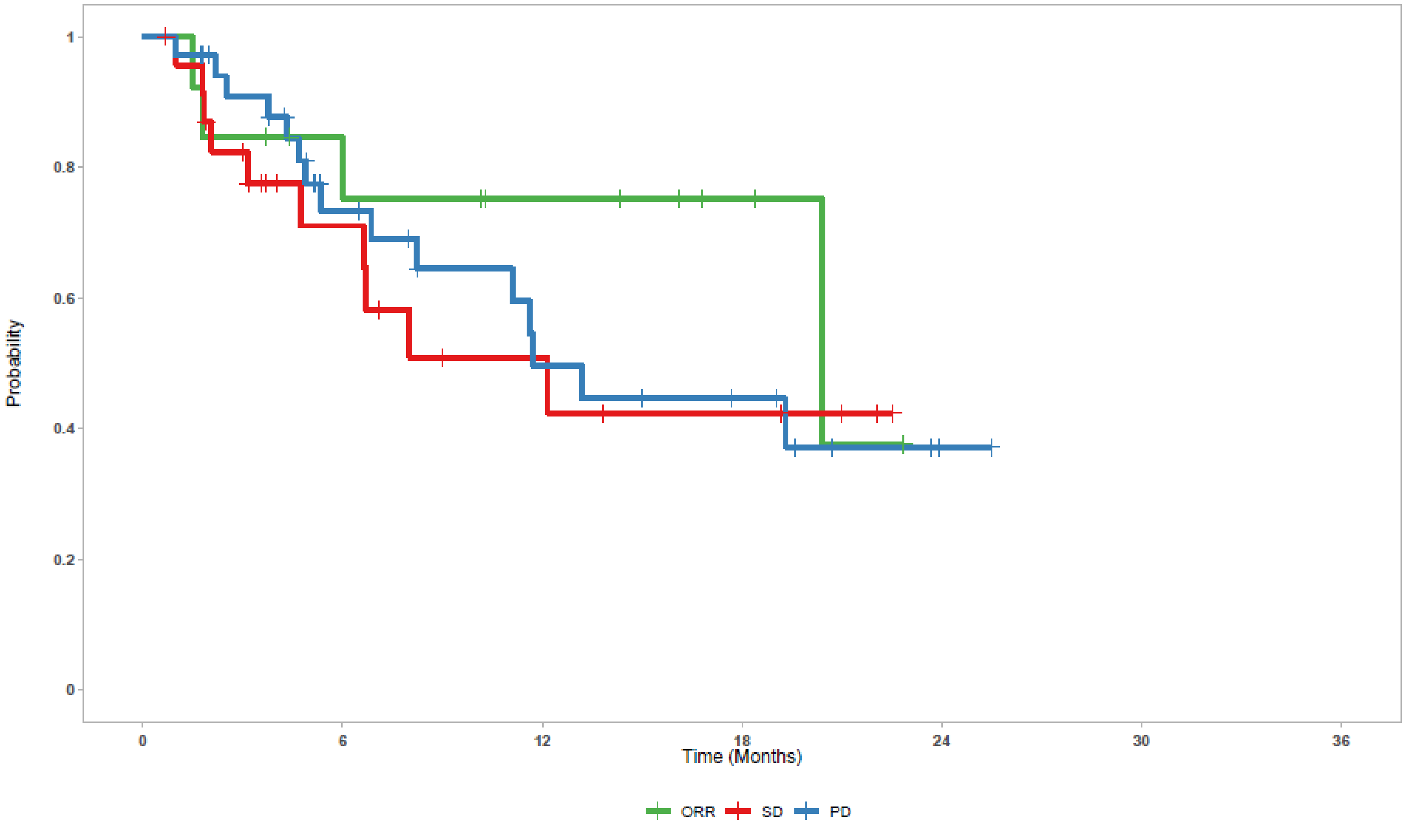

ORR	13	10	7	4	0	0	0
SD	24	12	7	5	0	0	0
PD	36	19	11	8	2	0	0

Table S1. Proportions of missing variables.

	**N (%)**
Age (years)	0 (0.0)
Sex	0 (0.0)
BMI (kg/m^2)	5 (2.1)
Type 2 diabetes	21 (9.0)
Cirrhosis	2 (0.9)
Aetiology	4 (1.7)
Previous portal hypertensive bleeding	20 (8.6)
Ascites	1 (0.4)
Hepatic encephalopathy	14 (6.0)
Platelet count (*10^9/L)	20 (8.6)
Albumin (g/dL)	21 (9.0)
Bilirubin (mg/dL)	20 (8.6)
INR	22 (9.4)
Child-Pugh class	0 (0.0)
ALBI score	21 (9.0)
ECOG-PS	0 (0)
AFP	5 (2.1)
Size of major nodule (cm)	22 (9.4)
Number of nodules > 3	20 (8.6)
Extrahepatic spread	22 (9.4)
Macrovascular invasion	1 (0.4)
BCLC stage C	23 (9.9)
STRIDE	0 (0.0)
First-line treatment	0 (0.0)

Table S2. Univariate analysis for overall survival in the whole cohort.

	**Hazard ratio**	**95% Confidence interval**	**p-value**
Age (years)	1.01	0.99 – 1.04	0.332
Male sex	1.26	0.69 – 2.32	0.454
BMI (kg/m^2)	0.96	0.91 – 1.01	0.111
Type 2 diabetes	1.17	0.65 – 2.08	0.598
Cirrhosis	1.24	0.71 – 2.18	0.447
Viral aetiology	1.01	0.65 – 1.56	0.963
Previous portal hypertensive bleeding	0.87	0.19 – 4.05	0.865
Gastroesophageal varices	1.30	0.78 – 2.16	0.313
Ascites	1.41	0.81 – 2.47	0.226
Hepatic encephalopathy	1.17	0.38 – 3.53	0.786
Platelet count (*10^9/L)	1.00	1.00 – 1.00	0.622
Albumin (g/dL)	0.60	0.41 – 0.88	0.001
Bilirubin (mg/dL)	1.20	0.94 – 1.53	0.148
INR	0.84	0.36 – 1.93	0.679
Child-Pugh class B	1.75	1.10 – 2.81	0.019
Child-Pugh score	1.26	1.08 – 1.46	0.003
ALBI grade 2/3	1.33	0.83 – 2.16	0.238
ALBI score	1.65	1.13 – 2.43	0.010
ECOG-PS (2 vs 0-1)	1.74	1.01-2.98	0.044
AFP $\geq$ 400 ng/mL	1.27	0.81 – 1.99	0.290
Size of major nodule (cm)	1.03	1.00 – 1.06	0.071
Number of nodules > 3	1.11	0.68 – 1.81	0.670
Extrahepatic spread	1.17	0.73 – 1.87	0.507
Macrovascular invasion	2.56	1.65 – 3.97	0.000
Vp4 portal invasion	1.17	0.59 – 2.34	0.651
BCLC stage C	1.97	1.17 – 3.29	0.010
STRIDE	1.15	0.61 – 2.16	0.658
First-line treatment	0.99	0.60 – 1.63	0.958
HIMALAYA-IN	0.61	0.39 – 0.96	0.032
HCC progression*	1.07	0.77 – 1.47	0.696
Hepatic decompensation	3.58	2.23-5.76	<0.001

*Considered as time-dependent covariates

Table S3. Univariate analysis for overall survival in HIMALAYA-IN patients.

	**Hazard ratio**	**95% Confidence interval**	**p-value**
Age (years)	1.07	1.03 – 1.11	0.001
Male sex	0.84	0.37 – 1.90	0.684
BMI (kg/m^2)	0.89	0.84 – 0.95	0.000
Type 2 diabetes	1.26	0.57 – 2.79	0.560
Cirrhosis	1.13	0.57 – 2.22	0.722
Viral aetiology	0.86	0.45 – 1.62	0.638
Previous portal hypertensive bleeding	2.17	0.32 – 14.74	0.426
Gastroesophageal varices	1.11	0.46 – 2.65	0.820
Ascites	0.46	0.09 – 2.27	0.338
Platelet count (*10^9/L)	1.00	1.00 – 1.00	0.142
Albumin (g/dL)	0.80	0.41 – 1.58	0.524
Bilirubin (mg/dL)	0.89	0.51 – 1.54	0.678
INR	0.65	0.13 – 3.25	0.602
Child-Pugh score 6	1.07	0.53 – 2.18	0.84
ALBI grade 2/3	1.00	0.54 – 1.88	0.989
ALBI score	1.26	0.69 – 2.32	0.452
ECOG-PS 1	0.79	0.40 – 1.55	0.502
AFP ≥ 400 ng/mL	0.79	0.40 – 1.54	0.485
Size of major nodule (cm)	1.01	0.97 – 1.05	0.482
Number of nodules > 3	1.21	0.60 – 2.45	0.600
Extrahepatic spread	0.79	0.39 – 1.61	0.512
Macrovascular invasion	3.02	1.60 – 5.73	0.001
BCLC stage C	1.48	0.76 – 2.87	0.25
STRIDE	5.25	0.67 – 41.14	0.114
HCC progression*	1.00	0.67 – 1.48	0.993
Hepatic decompensation*	3.29	1.46 – 7.39	<0.001

*Considered as time-dependent covariates

Table S4. Univariate and multivariate analysis for overall survival after imputation of missing data.

	Univariate analysis			Multivariate analysis		
	**Hazard ratio**	**95% Confidence interval**	**p-value**	**Hazard ratio**	**95% Confidence interval**	**p-value**
Age (years)	1.01	0.99 – 1.04	0.332			
Male sex	1.26	0.69 – 2.32	0.454			
BMI (kg/m^2)	1.05	0.95-1.15	0.376			
Type 2 diabetes	1.01	0.63-1.61	0.975			
Cirrhosis	1.39	0.81-2.39	0.233			
Viral aetiology	1.03	0.67-1.59	0.894			
Previous portal hypertensive bleeding	0.62	0.15-2.51	0.499			
Ascites	1.50	0.89-2.52	0.125			
Hepatic encephalopathy	0.94	0.31-2.90	0.919			
Platelet count ($*10^9/L$)	1.00	0.99-1.01	0.731			
INR	1.14	0.49-2.64	0.759			
Child-Pugh class B	1.75	1.10 – 2.81	0.019			
Child-Pugh score	1.26	1.08 – 1.46	0.003			
ALBI grade 2/3	1.41	0.87-2.27	0.159			
ALBI score	1.67	1.11-2.50	0.013	1.42	0.93-2.14	0.097
ECOG-PS (2 vs 0-1)	1.74	1.01-2.98	0.044	1.23	0.71-2.10	0.457
AFP $\geq$ 400 ng/mL	1.26	0.81-1.96	0.298			
Size of major nodule (cm)	1.04	0.96-1.13	0.316			
Number of nodules > 3	1.21	0.79-1.86	0.376			
Extrahepatic spread	1.22	0.78-1.93	0.383			
Macrovascular invasion	2.41	1.55-3.73	<0.001	2.13	1.35-3.35	0.001
Vp4 portal invasion	1.17	0.59 – 2.34	0.651			
BCLC stage C	1.92	1.18-3.13	0.009			
STRIDE	1.15	0.61 – 2.16	0.658			
First-line treatment	0.99	0.60 – 1.63	0.958			
HIMALAYA-IN	0.61	0.39 – 0.96	0.032			
HCC progression*	1.07	0.77 – 1.47	0.696			
Hepatic decompensation	3.58	2.23-5.76	<0.001	3.54	1.89-6.63	<0.001

Table S5. Progression-free survival of HIMALAYA-OUT patients stratified according to exclusion criteria.

	Child-Pugh B	Child-Pugh A	HR (95%CI)	ECOG-PS > 1	ECOG-PS 0-1	HR (95%CI)	Vp4	No Vp4	HR (95%CI)	>1° Line	1° Line	HR (95%CI)
Median progression-free survival (months)(95% CI)	3.8 (2.5-5.2)	5.0 (2.5-9.9)	1.18, (0.70-1.98, p=0.53)	4.4 (3.5-20.4)	3.6 (2.4-5.0)	0.69, (0.41-1.15, p=0.16)	5.2 (2.1-20.4)	3.7 (2.6-5.0)	0.77, (0.42-1.43, p=0.41)	4.3 (2.4-6.7)	3.7 (2.5-9.9)	0.92, (0.55-1.54, p=0.77)

Table S6. Progression-free survival of STRIDE and Durvalumab monotherapy in HIMALAYA-IN and HIMALAYA-OUT patients.

	HIMALAYA-IN			HIMALAYA-OUT		
	STRIDE	Durvalumab monotherapy	HR (95%CI, p-value)	STRIDE	Durvalumab monotherapy	HR (95%CI, p-value)
Median progression-free survival (months)(95%CI)	6.6 (6.0-11.1)	5.3 (1.9-11.3)	0.75 (0.27-2.08, p=0.58)	3.9 (2.9-6.7)	3.7 (2.5-20.4)	0.92 (0.54-1.59, p=0.77)

Table S7. Progression-free survival according to aetiology of liver disease in the whole cohort, in HIMALAYA-IN and HIMALAYA-OUT patients.

	Whole cohort			HIMALAYA-IN			HIMALAYA-OUT		
	Viral	Non-viral	HR (95%CI, p-value)	Viral	Non-viral	HR (95%CI, p-value)	Viral (n=32)	Non-viral (n=41)	HR (95%CI, p-value)
Median progression-free survival (months)(95%CI)	6.3 (4.3-9.1)	5.3 (3.7-6.6)	0.93 (0.66-1.32, p=0.70)	9.1 (6.0-15.2)	6.2 (3.3-8.4)	0.82. (0.51-1.32, p=0.41)	3.7 (2.5-5.2)	4.4 (2.4-9.9)	1.15, (0.69-1.92, p=0.59)

Table S8. Univariate analysis for progression-free survival in the whole cohort.

	**Hazard ratio**	**95% Confidence interval**	**p-value**
Age (years)	1.02	1.00 – 1.04	0.109
Male sex	1.00	0.64 – 1.54	0.985
BMI (kg/m^2)	0.97	0.93 – 1.01	0.115
Type 2 diabetes	1.01	0.64 – 1.60	0.958
Cirrhosis	1.17	0.78 – 1.76	0.449
Viral aetiology	0.93	0.66 – 1.32	0.702
Previous portal hypertensive bleeding	0.81	0.30 – 2.22	0.690
Gastroesophageal varices	1.36	0.93 – 2.00	0.114
Ascites	1.26	0.81 – 1.97	0.305
Hepatic encephalopathy	0.68	0.25 – 1.85	0.454
Platelet count ($*10^9/L$)	1.00	1.00 – 1.00	0.531
Albumin (g/dL)	0.73	0.53 - 1.00	0.051
Bilirubin (mg/dL)	1.24	1.01 - 1.52	0.035
INR	0.94	0.46 - 1.95	0.877
Child-Pugh class B	1.44	0.98 - 2.12	0.066
Child-Pugh score	1.14	1.00 - 1.31	0.047
ALBI grade 2/3	1.05	0.73 - 1.50	0.797
ALBI score	1.34	0.98 - 1.82	0.062
ECOG-PS (2 vs 0-1)	0.97	0.61 - 1.52	0.885
AFP $\geq$ 400 ng/mL	0.98	0.69 - 1.40	0.908
Size of major nodule (cm)	1.01	0.98 - 1.04	0.488
Number of nodules > 3	1.38	0.95 - 2.01	0.091
Extrahepatic spread	1.25	0.87 - 1.81	0.226
Macrovascular invasion	1.42	0.99 - 2.04	0.060
Vp4 portal invasion	0.91	0.49 - 1.70	0.776
BCLC stage C	1.41	0.96 - 2.09	0.082
STRIDE	0.88	0.57 - 1.39	0.603
First-line treatment	0.78	0.51 - 1.18	0.243
HIMALAYA-IN	0.74	0.52 - 1.05	0.091

Table S9. Univariate analysis for progression-free survival in HIMALAYA-IN patients.

	**Hazard ratio**	**95% Confidence interval**	**p-value**
Age (years)	1.07	1.03 - 1.10	<0.001
Male sex	0.86	0.48 - 1.53	0.607
BMI (kg/m^2)	0.95	0.89 - 1.00	0.064
Type 2 diabetes	1.15	0.63 - 2.09	0.646
Cirrhosis	1.06	0.64 - 1.74	0.821
Viral aetiology	0.82	0.51 - 1.32	0.413
Previous portal hypertensive bleeding	1.33	0.32 - 5.52	0.689
Gastroesophageal varices	1.41	0.79 - 2.51	0.246
Ascites	0.89	0.32 - 2.45	0.822
Platelet count (*10^9/L)	1.00	1.00 - 1.00	0.155
Albumin (g/dL)	1.18	0.72 - 1.95	0.512
Bilirubin (mg/dL)	0.95	0.58 - 1.57	0.856
INR	1.19	0.41 - 3.46	0.748
Child-Pugh score	0.79	0.46 - 1.38	0.415
ALBI grade 2/3	0.79	0.50 - 1.26	0.325
ALBI score	0.83	0.51 - 1.36	0.467
ECOG-PS 1	1.14	0.70 - 1.85	0.591
AFP $\geq$ 400 ng/mL	0.66	0.40 - 1.09	0.107
Size of major nodule (cm)	1.00	0.96 - 1.05	0.853
Number of nodules > 3	1.06	0.64 - 1.78	0.813
Extrahepatic spread	1.17	0.72 - 1.92	0.526
Macrovascular invasion	1.52	0.89 - 2.58	0.123
BCLC stage C	1.35	0.82 - 2.22	0.240
STRIDE	0.77	0.31 - 1.93	0.585

Table S10. Univariate competing risks analysis for hepatic decompensation in the whole cohort.

	Univariate analysis			Multivariable analysis		
	**Subdistribution Hazard ratio**	**95% Confidence interval**	**p-value**	**Subdistribution Hazard ratio**	**95% Confidence interval**	**p-value**
Age (years)	0.98	0.95 - 1.02	0.319			
Male sex	1.22	0.32 - 4.62	0.767			
BMI (kg/m^2)	1.01	0.93 - 1.10	0.775			
Type 2 diabetes	0.68	0.16 - 2.96	0.609			
Cirrhosis	1.66	0.50 - 5.52	0.406			
Viral aetiology	1.58	0.61 - 4.09	0.347			
Previous portal hypertensive bleeding	2.09	0.61 - 4.09	0.392			
Gastroesophageal varices	0.53	0.15 - 1.83	0.318			
Platelet count (*10^9/L)	0.99	0.98 - 1.01	0.655			
Albumin (g/dL)	0.62	0.30 - 1.25	0.180			
Bilirubin (mg/dL)	1.34	0.90 - 1.99	0.145			
INR	0.73	0.15 - 3.34	0.684			
Child-Pugh score	1.37	0.93 - 2.02	0.110			
ALBI grade 2/3	1.39	0.49 - 3.89	0.559			
ALBI score	1.83	0.95 - 3.54	0.072	1.67	0.85-3.28	0.135
ECOG-PS 2 (vs 0-1)	1.84	0.58 - 5.80	0.296			
AFP $\geq$ 400 ng/mL	0.99	0.98 - 1.01	0.611			
Size of major nodule (cm)	1.04	0.99 - 1.08	0.056	1.03	0.99-1.08	0.111
Number of nodules > 3	2.66	0.89 - 7.97	0.080	2.50	0.83-7.51	0.102
Extrahepatic spread	1.52	0.53 - 4.32	0.435			
Macrovascular invasion	1.77	0.69 - 4.54	0.237			
BCLC stage C	2.20	0.64 - 7.61	0.213			
STRIDE	0.97	0.37 - 2.53	0.949			
First-line treatment	1.58	0.36-6.94	0.541			

Table S11. Objective response rate of HIMALAYA-OUT patients stratified according to exclusion criteria.

	Child-Pugh B (n=38)	Child-Pugh A (n=35)	p-value	ECOG-PS > 1 (n=22)	ECOG-PS 0-1 (n=51)	p-value	Vp4 (n=14)	No Vp4 (n=59)	p-value	>1° Line (n=31)	1° Line (n=42)	p-value
CR	1 (2.6)	1 (2.9)	0.994	0 (0.0)	2 (3.9)	0.468	0 (0.0)	2 (3.4)	0.272	1 (3.2)	1 (2.4)	0.373
PR	6 (15.8)	5 (14.3)		5 (22.7)	6 (11.8)		5 (35.7)	6 (10.2)		3 (9.7)	8 (19.0)	
SD	12 (31.6)	12 (34.3)		8 (36.4)	16 (31.4)		3 (31.4)	21 (35.6)		10 (32.3)	14 (33.3)	
PD	19 (50.0)	17 (48.6)		9 (40.9)	27 (52.9)		6 (42.9)	30 (50.8)		17 (54.8)	19 (45.2)	
ORR	7 (18.4)	6 (17.1)	0.887	5 (22.7)	8 (15.7)	0.474	5 (35.7)	8 (13.5)	0.053	4 (12.9)	9 (21.4)	0.350
DCR	19 (50.0)	18 (51.4)	0.904	13 (59.1)	24 (47.1)	0.349	8 (57.1)	29 (49.1)	0.593	14 (45.2)	25 (59.5)	0.421

Table S12. Objective response rate of STRIDE and Durvalumab monotherapy in HIMALAYA-IN and HIMALAYA-OUT patients.

	Whole cohort (n=187)			HIMALAYA-IN (n=114)			HIMALAYA-OUT (n=73)		
	STRIDE (n=155)	Durvalumab monotherapy (n=32)	p-value	STRIDE (n=109)	Durvalumab monotherapy (n=5)	p-value	STRIDE (n=46)	Durvalumab monotherapy (n=27)	p-value
CR	9 (5.8)	0 (0.0)	0.226	7 (6.4)	0 (0.0)	0.365	2 (4.3)	0 (0.0)	0.945
PR	26 (16.8)	5 (15.6)		20 (18.3)	0 (0.0)		6 (13.0)	5 (18.5)	
SD	59 (38.1)	12 (37.5)		44 (40.4)	3 (60.0)		15 (32.6)	9 (33.3)	
PD	61 (39.4)	15 (46.9)		38 (34.9)	2 (40.0)		23 (50.0)	13 (48.1)	
ORR	35 (22.6)	5 (15.6)	0.383	27 (24.8)	0 (0.0)	0.205	8 (17.4)	5 (18.5)	0.904
DCR	94 (60.6)	17 (53.1)	0.432	71 (65.1)	3 (60.0)	0.815	23 (50.0)	14 (51.9)	0.879

Table S13. Objective response rate according to aetiology of liver disease in the whole cohort, in HIMALAYA-IN and HIMALAYA-OUT patients.

	Whole cohort (n=187)			HIMALAYA-IN (n=114)			HIMALAYA-OUT (n=73)		
	Viral (n=84)	Non-viral (n=103)	p-value	Viral (n=52)	Non-viral (n=62)	p-value	Viral (n=32)	Non-viral (n=41)	p-value
CR	3 (3.6)	6 (5.8)	0.625	2 (3.8)	5 (8.1)	0.786	1 (3.1)	1 (2.4)	0.605
PR	15 (17.9)	16 (15.5)		11 (21.2)	9 (14.5)		4 (12.5)	7 (17.1)	
SD	30 (35.7)	41 (39.8)		20 (38.5)	27 (43.5)		10 (31.2)	14 (34.1)	
PD	36 (42.9)	40 (38.8)		19 (36.5)	21 (33.9)		17 (53.1)	19 (46.3)	

ORR	18 (21.4)	22 (21.4)	0.991	13 (25.0)	14 (22.6)	0.763	5 (15.6)	8 (19.5)	0.667
DCR	48 (57.1)	63 (61.2)	0.578	33 (63.5)	41 (66.1)	0.767	15 (46.9)	22 (53.7)	0.568

Table S14. Univariate and multivariate logistic regression analysis for objective response rate in the whole cohort.

	**Univariate analysis**			**Multivariable analysis**		
	**Odds ratio**	**95% Confidence interval**	**p-value**	**Odds ratio**	**95% Confidence interval**	**p-value**
Age (years)	0.97	0.94-1.01	0.130			
Male sex	0.58	0.23-1.46	0.248			
BMI (kg/m^2)	1.02	0.94-1.09	0.649			
Type 2 diabetes	1.03	0.44-2.42	0.950			
Cirrhosis	0.78	0.34-1.80	0.568			
Viral aetiology	1.02	0.50-2.08	0.955			
Gastroesophageal varices	0.87	0.39-1.96	0.746			
Ascites	1.78	0.71.4.48	0.222			
Hepatic encephalopathy	0.51	0.06-4.35	0.545			
Platelet count (*10^9/L)	1.00	0.99-1.01	0.884			
Albumin (g/dL)	0.53	0.29-0.98	0.042			
Bilirubin (mg/dL)	0.99	0.591-67	0.975			
INR	2.20	0.68-7.14	0.189			
Child-Pugh class B	0.86	0.34-2.15	0.746			
Child-Pugh score	1.04	0.77-1.40	0.816			
ALBI grade 2/3	1.85	0.85-4.00	0.121			
ALBI score	1.73	0.95-3.14	0.073	1.59	0.86-3.00	0.145
ECOG-PS 2 (vs 0-1)	1.12	0.38-3.28	0.834			
AFP $\geq$ 400 ng/mL	2.36	1.14-4.89	0.021	2.17	1.01-4.64	0.047
Size of major nodule (cm)	0.99	0.94-1.06	0.937			
Number of nodules > 3	0.57	0.26-1.23	0.153			
Extrahepatic spread	1.32	0.60-2.89	0.486			
Macrovascular invasion	2.08	0.97-4.43	0.059	1.45	0.65-3.23	0.364
Vp4 portal invasion	2.11	0.66-6.70	0.206			
BCLC stage C	2.24	0.97-5.16	0.058			
STRIDE	1.58	0.56-4.43	0.385			
First-line treatment	2.13	0.70-6.50	0.185			
HIMALAYA-IN	1.41	0.67-2.97	0.370			

Table S15. Univariate and multivariable logistic regression analysis for objective response rate in HIMALAYA-IN patients.

	Univariate analysis			Multivariable analysis		
	**Odds ratio**	**95% Confidence interval**	**p-value**	**Odds ratio**	**95% Confidence interval**	**p-value**
Age (years)	0.94	0.89-1.00	0.051	0.93	0.85-0.99	0.029
Male sex	0.36	0.11-1.17	0.091	0.87	0.09-2.81	0.440
BMI (kg/m^2)	1.02	0.92-1.13	0.740			
Type 2 diabetes	1.13	0.40-3.18	0.811			
Cirrhosis	1.17	0.45-30.03	0.750			
Viral aetiology	1.19	0.49-2.88	0.700			
Gastroesophageal varices	0.68	0.21-2.25	0.533			
Ascites	1.67	0.29-9.73	0.566			
Platelet count (*10^9/L)	1.00	0.99-1.01	0.292			
Albumin (g/dL)	0.30	0.12-0.78	0.013			
Bilirubin (mg/dL)	0.72	0.26-1.99	0.528			
INR	0.19	0.01-8.98	0.396			
Child-Pugh score	1.42	0.55-3.65	0.467			
ALBI grade 2/3	2.21	0.89-5.47	0.085			
ALBI score	2.88	1.07-7.72	0.035	3.24	0.96-10.86	0.058
AFP $\geq$ 400 ng/mL	2.67	1.06-6.68	0.036	2.00	0.66-6.15	0.221
Size of major nodule (cm)	0.99	0.92-1.06	0.767			
Number of nodules > 3	0.55	0.21-1.42	0.216			
Extrahepatic spread	1.46	0.56-3.78	0.439			
Macrovascular invasion	1.65	0.61-4.43	0.323			
BCLC stage C	2.31	0.86-6.18	0.096	2.63	0.80-8.72	0.113

Table S16. Treatment-related adverse events in the whole cohort and in HIMALAYA-IN patients.

	Whole cohort		HIMALAYA-IN patients	
**Toxicities**	**Any-grade TRAEs**	**Grade 3-4 TRAEs**	**Any-grade TRAEs**	**Grade 3-4 TRAEs**
Skin	35 (15.0%)	4 (1.7%)	25 (20.3%)	4 (3.2%)
Diarrhoea/Colitis	27 (11.6%)	11 (4.7%)	20 (16.3%)	8 (6.5%)
Fatigue	23 (9.9%)	0 (0.0%)	12 (9.7%)	0 (0.0%)
Thyroid toxicity	15 (6.4%)	3 (1.3%)	14 (11.4%)	3 (2.4%)
Hepatotoxicity	10 (4.3%)	9 (3.9%)	5 (4.1%)	5 (4.1%)
Pneumonitis	6 (2.6%)	5 (2.1%)	4 (3.2%)	3 (2.4%)
Pituitary	5 (2.1%)	1 (0.4%)	2 (1.6%)	0 (0.0%)
Rheumatological Toxicities	3 (3.1%)	2 (0.8%)	3 (2.4%)	2 (1.6%)
Others	16 (6.9%)	5 (2.1%)	14 (11.4%)	4 (3.2%)

Table S17. Safety results of HIMALAYA-OUT patients stratified according to exclusion criteria.

	Child-Pugh B (n=70)	Child-Pugh A (n=40)	p-value	ECOG-PS > 1 (n=54)	ECOG-PS 0-1 (n=56)	p-value	Vp4 (n=22)	No Vp4 (n=88)	p-value	>1° Line (n=47)	1° Line (n=63)	p-value
Grade 3-4 TRAEs	6 (8.6)	7 (17.5)	0.165	5 (9.3)	8 (14.3)	0.416	6 (27.3)	7 (8.0)	0.013	9 (19.1)	4 (6.3)	0.041

Table S18. Safety results of STRIDE and durvalumab monotherapy in HIMALAYA-IN and -OUT patients.

	HIMALAYA-IN			HIMALAYA-OUT		
	STRIDE (n=117)	Durvalumab monotherapy (n=6)	p-value	STRIDE (n=75)	Durvalumab monotherapy (n=35)	p-value
Grade 3-4 TRAEs	23 (19.7)	2 (33.3)	0.419	8 (10.7)	5 (14.3)	0.059

Table S19. Safety according to aetiology of liver disease in the whole cohort, in HIMALAYA-IN and HIMALAYA-OUT patients.

	Whole cohort			HIMALAYA-IN			HIMALAYA-OUT		
	Viral (n=108)	Non-viral (n=125)	p-value	Viral (n=57)	Non-viral (n=66)	p-value	Viral (n=51)	Non-viral (n=59)	p-value
Grade 3-4 TRAEs	22 (20.4)	16 (12.8)	0.120	12 (21.1)	13 (19.7)	0.853	10 (19.6)	3 (5.1)	0.019

Table S20. Univariate logistic regression analysis for grade 3-4 treatment-related adverse events (TRAEs) in the whole cohort.

	Univariate analysis		
	**Odds ratio**	**95% Confidence interval**	**p-value**
Age (years)	0.98	0.94 - 1.01	0.180
Male sex	1.05	0.40 - 2.71	0.924
BMI (kg/m^2)	1.00	0.93 - 1.08	0.894
Type 2 diabetes	0.74	0.32 - 1.74	0.490
Cirrhosis	0.66	0.30 - 1.45	0.301
Viral aetiology	1.74	0.86 - 3.52	0.122
Previous portal hypertensive bleeding	0.40	0.05 - 3.18	0.384
Gastroesophageal varices	0.53	0.21 - 1.34	0.179
Ascites	0.44	0.15 - 1.32	0.142
Hepatic encephalopathy	0.48	0.06 - 3.85	0.488
Platelet count (*10^9/L)	1.00	1.00 - 1.00	0.755
Albumin (g/dL)	0.92	0.51 - 1.67	0.794
Bilirubin (mg/dL)	0.70	0.39 - 1.27	0.246
INR	0.40	0.05 - 2.84	0.357
Child-Pugh class B	0.38	0.15 - 0.96	0.042
Child-Pugh score	0.78	0.58 - 1.05	0.101
ALBI grade 2/3	1.37	0.64 - 2.94	0.412
ALBI score	0.98	0.54 - 1.76	0.937
ECOG-PS 2 (vs 0-1)	0.45	0.17 - 1.22	0.117
AFP $\geq$ 400 ng/mL	0.98	0.47 - 2.01	0.951
Size of major nodule (cm)	1.00	0.94 - 1.06	0.919
Number of nodules > 3	1.03	0.49 - 2.15	0.934
Extrahepatic spread	1.02	0.49 - 2.14	0.954
Macrovascular invasion	1.37	0.66 - 2.83	0.399
Vp4 portal invasion	2.10	0.76 - 5.76	0.151
BCLC stage C	0.98	0.47 - 2.05	0.954
STRIDE	0.93	0.38 - 2.30	0.884
First-line treatment	0.78	0.34 - 1.78	0.556
HIMALAYA-IN	1.90	0.92 - 3.94	0.082

Table S21. Univariate and multivariable logistic regression analysis for grade 3-4 treatment-related adverse events (TRAEs) in HIMALAYA-IN patients.

	**Univariate analysis**		
	**Odds ratio**	**95% Confidence interval**	**p-value**
Age (years)	0.94	0.89 - 0.99	0.026
Male sex	1.32	0.35 - 4.99	0.677
BMI (kg/m^2)	1.03	0.93 - 1.14	0.549
Type 2 diabetes	1.00	0.36 - 2.75	0.996
Cirrhosis	0.81	0.32 - 2.03	0.651
Viral aetiology	1.09	0.45 - 2.62	0.852
Previous portal hypertensive bleeding	1.23	0.12 - 12.40	0.859
Gastroesophageal varices	0.74	0.23 - 2.41	0.620
Ascites	0.56	0.07 - 4.83	0.602
Platelet count (*10^9/L)	1.00	1.00 - 1.00	0.523
Albumin (g/dL)	0.70	0.30 - 1.66	0.420
Bilirubin (mg/dL)	0.49	0.16 - 1.46	0.203
INR	0.65	0.06 - 6.73	0.715
Child-Pugh score	1.24	0.48 - 3.20	0.660
ALBI grade 2/3	2.00	0.82 - 4.89	0.129
ALBI score	1.27	0.51 - 3.15	0.608
ECOG-PS	0.59	0.22 - 1.54	0.280
AFP $\geq$ 400 ng/mL	1.35	0.55 - 3.36	0.512
Size of major nodule (cm)	0.99	0.93 - 1.06	0.877
Number of nodules > 3	0.79	0.31 - 1.99	0.614
Extrahepatic spread	1.07	0.41 - 2.76	0.887
Macrovascular invasion	1.51	0.58 - 3.96	0.398
BCLC stage C	0.92	0.38 - 2.24	0.853
STRIDE	0.49	0.08 - 2.84	0.425
